# No Evidence of Harms of Probiotic *Lactobacillus rhamnosus* GG ATCC 53103 in Healthy Elderly—A Phase I Open Label Study to Assess Safety, Tolerability and Cytokine Responses

**DOI:** 10.1371/journal.pone.0113456

**Published:** 2014-12-01

**Authors:** Patricia L. Hibberd, Lauren Kleimola, Anne-Maria Fiorino, Christine Botelho, Miriam Haverkamp, Irina Andreyeva, Debra Poutsiaka, Claire Fraser, Gloria Solano-Aguilar, David R. Snydman

**Affiliations:** 1 Division of Global Health, Massachusetts General Hospital for Children, Boston, MA, United States of America; 2 Division of Geographic Medicine and Infectious Diseases, Tufts Medical Center, Boston, MA, United States of America; 3 Institute for Genome Sciences, University of Maryland School of Medicine, Baltimore, MD, United States of America; 4 United States Department of Agriculture, Agricultural Research Service, Beltsville Human Nutrition Research Center, Diet, Genomics, and Immunology Laboratory, Beltsville, MD, United States of America; Universidade do Extremo Sul Catarinense, Brazil

## Abstract

**Background:**

Although *Lactobacillus rhamnosus* GG ATCC 53103 (LGG) has been consumed by 2 to 5 million people daily since the mid 1990s, there are few clinical trials describing potential harms of LGG, particularly in the elderly.

**Objectives:**

The primary objective of this open label clinical trial is to assess the safety and tolerability of 1×10^10^ colony forming units (CFU) of LGG administered orally twice daily to elderly volunteers for 28 days. The secondary objectives were to evaluate the effects of LGG on the gastrointestinal microbiome, host immune response and plasma cytokines.

**Methods:**

Fifteen elderly volunteers, aged 66–80 years received LGG capsules containing 1×10^10^ CFU, twice daily for 28 days and were followed through day 56. Volunteers completed a daily diary, a telephone call on study days 3, 7 and 14 and study visits in the Clinical Research Center at baseline, day 28 and day 56 to determine whether adverse events had occurred. Assessments included prompted and open-ended questions.

**Results:**

There were no serious adverse events. The 15 volunteers had a total of 47 events (range 1–7 per volunteer), 39 (83%) of which were rated as mild and 40% of which were considered related to consuming LGG. Thirty-one (70%) of the events were expected, prompted symptoms while 16 were unexpected events. The most common adverse events were gastrointestinal (bloating, gas, and nausea), 27 rated as mild and 3 rated as moderate. In the exploratory analysis, the pro-inflammatory cytokine interleukin 8 decreased during LGG consumption, returning towards baseline one month after discontinuing LGG (p = 0.038) while there was no difference in other pro- or anti-inflammatory plasma cytokines.

**Conclusions:**

*Lactobacillus rhamnosus* GG ATCC 53103 is safe and well tolerated in healthy adults aged 65 years and older.

**Trial Registration:**

ClinicalTrials.gov NCT 01274598

## Introduction

Probiotics are live microorganisms which confer a health benefit on the host when administered in adequate amounts [1]. Until recently, probiotics were mostly found in yogurts and fermented milks, but probiotics are increasingly being found in a wide range of non-dairy drinks, nutrition bars, breakfast cereals, infant formula, relishes, condiments, sweeteners, candy, and pizza crust [2]. Probiotics are also sold as dietary supplements in capsules, tablets and powders and are widely available in drug stores, health food shops, supermarkets and on the Internet. The range of organisms that are sold as probiotics is ever increasing, as are the types of products containing probiotics, the health benefits that they are supposed to provide, and the volume of probiotic products being sold [3].

Probiotics have been receiving more attention from the scientific community, mostly as a result of the Human Microbiome Project that is studying culture independent methods to characterize microbial communities in the oral cavity, skin, vagina, gut and respiratory tract as well as the metabolic profiles produced by these microbial communities [4]–[7]. To date, the Human Microbiome Project has mostly focused on the differences in the microbial communities and metabolic profiles in health and disease states. This analytic approach is of great interest to understanding whether probiotics can or cannot be designed to establish or restore a “healthy” microbiome/metabolome across the lifespan [8]–[11]. Concurrently, the US Food and Drug Administration (FDA) has issued guidance indicating that an investigational new drug (IND) application is needed when live microorganisms are used to treat or prevent disease [12]. Even for probiotics that have been in widespread use as foods or dietary supplements, studies assessing clinical effects must comply with FDA product quality and follow standard drug development procedures. This requirement is supported by the Southern California Evidence-based Practice Center (EPC) report on the safety of probiotics to reduce risk and prevent or treat disease [13] which concluded that there has been a lack of assessment and systematic reporting of adverse events in the published trials and the literature is “not well equipped to answer questions on the safety of probiotic interventions with confidence” [13].

*Lactobacillus rhamnosus* GG ATCC 53103 (LGG) is one of the most studied probiotics. LGG is a commensal bacteria that occurs naturally in the human gastrointestinal tract and was originally isolated by Drs. Gorbach and Goldin in 1983 and patented as a probiotic in 1985, based in part on its ability to resist acid and bile so that it would survive transit through the stomach and the intestines and its ability to attach to the intestinal mucosa [14]. Its precise pharmacologic effects and mechanisms of action are not known but include colonization resistance in the gastrointestinal and respiratory tracts [15], [16]; immune modulation [17]–[19] and direct antimicrobial effects [20]–[23]. LGG is reported to be effective in preventing and treating diarrhea and other conditions [24], [25]. It is also promising for the prevention of respiratory tract infections in adults and young children [26]. LGG, like other lactobacilli, are rarely pathogenic, although there are rare case reports of LGG causing invasive disease, mostly in immunocompromised patients or those with indwelling catheters who were consuming LGG [27]–[33]. However, based on the Southern California EPC's concerns, information on the safety profile of LGG could be improved.

Based on the immunomodulatory properties of LGG, its role as a possible mucosally administered vaccine adjuvant [34]–[36], and suboptimal influenza vaccine efficacy in the elderly [37], we were funded by the National Center for Complementary and Alternative Medicine at NIH in 2006 to conduct a randomized trial of the probiotic LGG to evaluate its effect on the immune response to the inactivated and live attenuated influenza vaccine in healthy elderly volunteers. The FDA Center for Biological Evaluation and Research [FDA/CBER] asked us to complete a Phase I open label study under IND to evaluate the safety and tolerability of the use of LGG in healthy elderly volunteers, without administration of the influenza vaccine, prior to studying the effect of LGG on inactivated and live attenuated influenza vaccine. The development of this probiotic intervention is under FDA oversight that regulates “fit for intended purpose” product labeling and improved quality control. We conducted the FDA/CBER required Phase I open label study in healthy elderly paying particular attention to the CONSORT Statement Extension of better reporting of harms in randomized trials [38] and the recent call for improvements in adverse event reporting [39], [40].

The primary aim of this Phase I open label study was to provide information on adverse events that may occur in healthy elderly volunteers receiving LGG administered twice a day for 28 days. The secondary aims were to evaluate potential mechanisms of action of LGG in the healthy elderly by studying their gastrointestinal microbiome (C Fraser, manuscript in preparation) as well as immunologic responses to consumption of LGG for 28 days (G Solano-Aguilar, manuscript in preparation). This paper describes the detailed adverse event profile of the elderly volunteers and their serum cytokine profiles, particularly changes in pro- and anti-inflammatory cytokines [41].

## Materials and Methods

### Ethics statement

The procedures followed were in accordance with the ethical standards of Massachusetts General Hospital after approval by the Partners Human Research Committee (IRB # 2010P001695/MGH). A study physician explained the study procedures to the potential volunteer in detail, along with known possible harms of participation in the research. Written informed consent was obtained from all volunteers at the start of the screening visit and prior to enrollment in the study. The protocol for this trial and supporting CONSORT checklist are available as supporting information (see Protocol S1 and Checklist S1). The trial was registered at ClinicalTrials.gov (NCT01274598).

### Study volunteers

**Figure 1:**
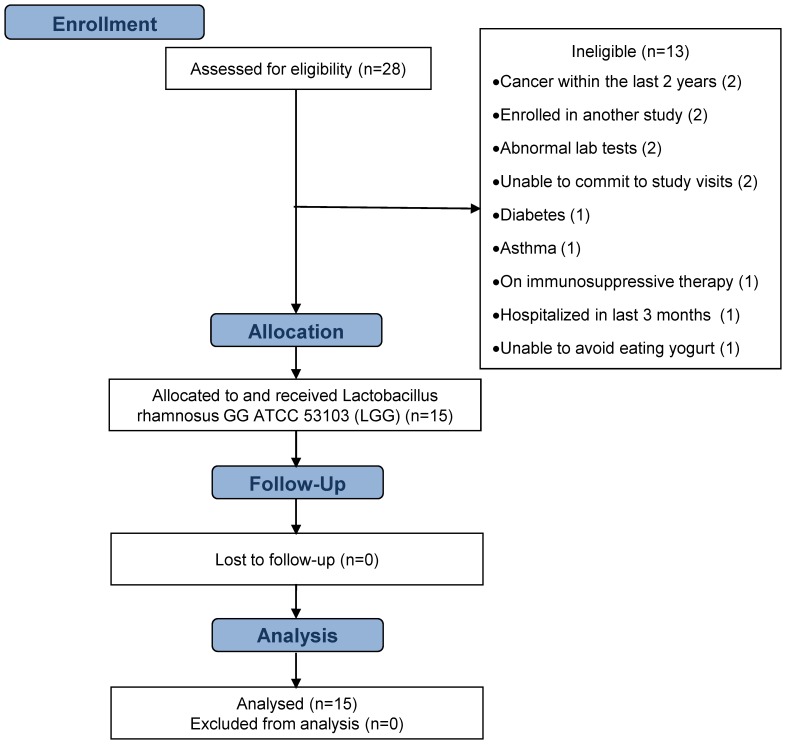
Participant flow diagram.

Twenty-eight volunteers were screened for eligibility (Table 1 shows the complete listing of inclusion and exclusion criteria). Of the 28 screened, 15 met all criteria and were enrolled in the study (Figure 1, participant flow diagram). During screening, we asked volunteers to identify their racial and ethnic category.

**Table 1:** Inclusion and exclusion criteria.

Inclusion Criteria	Exclusion Criteria
1. Adult male and female age 65–80 years	1. Resident of a nursing home or rehabilitation center
2. Has a primary care physician	2. Hospitalization, major surgery or endoscopy within the last 3 months or scheduled hospital admission within 3 months of enrollment
3. In the past two years:	3. Consumption of any probiotic or yogurt that has the “live and active cultures” seal during the 28 days before the baseline visit and unwillingness to forgo these products during the 56 day study period
a. Is community-dwelling	4. Received oral or parenteral antibiotics during the 28 days before the baseline visit
b. Has had a routine physical examination	5. Current or planned oral or parenteral immunosuppressive therapy, chemotherapy or radiotherapy
c. Has no new chronic conditions diagnosed	6. Allergy to probiotics, *Lactobacillus*, all antibiotics that could be used to treat Lactobacillus infection, microcrystaline cellulose or gelatin
4. Has received and is up to date on the following:	7. Diarrhea, constipation or vomiting during the 28 days before the baseline visit
a. Pneumococcal vaccination	8. Serious gastrointestinal illness including chronic liver disease, gastrointestinal surgery, cancer, inflammatory bowel disease, pancreatitis or motility disorder in the last 2 years
b. Mammography (women only)	9. History of drug or alcohol abuse in the previous 12 months
c. Screening colonoscopy	10. History of structural heart disease, endocarditis, valve replacement, Stage IV congestive heart failure, peripheral vascular disease, stroke, chronic obstructive pulmonary disease, asthma, collagen, vascular or autoimmune disease, end stage renal disease, diabetes, thyroid disease or active tuberculosis
5. Willing and able to comply with the protocol and participate for the planned duration of the study	11. Presence of an indwelling catheter, implanted hardware/prosthetic device or feeding tube
6. Completes the informed consent process	12. Any Grade 2 or higher abnormal vital sign or physical examination finding (FDA Toxicity Grading Scale)
	13. Positive HIV antibody, hepatitis B surface antigen or hepatitis C antibody
	14. Positive urine toxicology or positive breathalyzer
	15. Any Grade 2 or higher abnormal screening laboratory test (FDA Toxicity Grading Scale)
	16. Enrolled in another study

### Study design

This Phase I open label clinical trial designed to evaluate the safety and tolerability of LGG in 10–15 elderly volunteers was requested by the FDA. Oversight and monitoring prior to and during the conduct of this study was provided by an independent Data Safety Monitoring Board (DSMB), FDA/CBER, under IND 14377 and NIH/NCCAM Office of Clinical and Regulatory Affairs.

### Intervention

The study drug was supplied in gelatin capsules at a dose of 1×10^10^ colony forming units of *Lactobacillus rhamnosus* GG ATCC 53103 and 250 micrograms of microcrystalline cellulose. The capsules were encapsulated by Garden State Nutritionals, Fairfield, NJ and contained LGG manufactured by Chr. Hansen, Denmark and microcrystaline cellulose (Emcocel) manufactured by JRS Pharma, Rosenberg, Germany. The capsules were provided by Amerifit Brands Inc, Cromwell, Connecticut. Capsules were administered orally twice a day – once in the morning and once in the evening for 28 days. This dose was chosen because LGG can be recovered from stool specimens when administered at this dose [42]. Study drug was dispensed by the MGH Research Pharmacy.

### Study protocol

Study volunteers were seen in the MGH Clinical Research Centers according to the schedule shown in Table 2. On December 1, 2010 we started phone screening for study volunteers. The last volunteer exited the study on August 5, 2011. Prior to the screening visit, all volunteers were informed about the study and, if interested in proceeding, they provided written, informed consent to participate, as well as consent for testing for Human Immunodeficiency Virus (HIV), because of the low, but possible, risk of developing invasive LGG infection in HIV infected individuals. They were also informed that screening laboratory tests included testing for Hepatitis B surface antigen (HbsAg) and Hepatitis C antibody (anti-HCV) as well as for illicit drugs and alcohol. Subjects were informed that positive tests for HIV, HbsAg or anti-HCV and Hepatitis C would be reported to the Massachusetts Department of Public Health. Study physicians (PLH, MH) conducted all study visits.

**Table 2:** Study design.

	SCREENING VISIT	BASELINE VISIT	PHONE CALLS	END OF TREATMENT	END OF STUDY VISIT
STUDY DAY	−30 to −1	0	3, 7, 14	28	56
Review study and complete informed consent process	X				
Demographics	X				
Medical and Diet History	X				
Vital signs and physical exam	X			X	X
Screening laboratory studies^1^	X				
Determine eligibility to proceed	X				
Interval history, yogurt and probiotic consumption, review of medications (prescription and non-prescription), and dietary supplements		X		X	X
Review of symptoms, supplements		X			
Review of symptoms, physical exam		X			
Safety laboratory studies^2^		X		X	X
Breathalyzer for alcohol		X			
Sub-study review and informed consent		X			
Stool studies^3^		X		X	X
Immune response research studies^4^		X		X	X
Dispensing of LGG, observed administration of first dose		X			
Capsule culture^5^		X		X	
Review of symptoms, adverse event monitoring			X	X	X
Compliance			X	X	
Daily completion of symptom diary		X	X	X	X

1 Complete blood count, platelets, basic metabolic panel, liver function tests, HIV antibody (after consent), Hepatitis B surface antigen and Hepatitis C antibody tests, urine toxicology screen and blood alcohol level.

2 Complete blood count, platelets, basic metabolic panel, liver function tests.

3 Microbiome, LGG culture and identification.

4 PAXgene mRNA, PAXgene DNA, cytokines.

5 LGG culture and identification.

#### Screening visit

The procedures for the screening visit are shown in Table 2. At the end of the visit, volunteers were provided with information on foods and probiotic products to avoid prior to the baseline visit and during the study and were provided information on how to collect stool samples using a kit, should they be eligible to participate in the study. Volunteers who were eligible to participate after screening, based on review of all study data and laboratory tests, were informed of their status by the study coordinator within 7 days of the screening visit, and the baseline visit was scheduled (within 30 days of the screening visit). Ineligible volunteers were contacted as soon as possible by a study physician. Volunteers testing positive for HIV were asked to return for an additional visit so that the study physician could explain the test result, provide information and refer them for further counseling and evaluation. All volunteers with abnormal laboratories received a copy of their results so that they could discuss them with their primary care physician.

#### Baseline visit

Procedures for the baseline visit are shown in Table 2 and information on expected adverse events that could occur during the study was obtained using a combination of prompted and then open-ended questions (Materials and Methods S1). We collected stool specimens for microbiome studies prior to LGG administration. Volunteers were given instructions and materials to properly collect a stool specimen, including that the specimen should be collected no more than 24 hours before the appointment, no urine should enter the specimen container, and once the specimen is collected it is to be stored in a provided Sytrofoam box surrounded by frozen gel packs. Stool specimens were refrigerated upon arrival at the Clinical Research Center until they could be processed and aliquotted by the laboratory later on the same day. Fresh stool aliquots were sent for LGG culture and colony counts, and the remaining aliquots were frozen at −80°C for batched LGG PCR and metagenomics and metatranscriptomics prior to LGG administration. Participating volunteers received their first dose of LGG under observation of the study physicians, prior to discharge. They were instructed to take 2 capsules of LGG a day for 28 days. Extra capsules were dispensed to check product quality – one capsule was sent for LGG culture and colony counts. Volunteers were reminded to contact the study physician or study coordinator at any time regarding any study related issues, including any symptoms or adverse events. Volunteers were also asked to complete a daily diary of symptoms that could be associated with LGG and to add other symptoms not on the prompted list (Materials and Methods S1).

#### Telephone follow-up

Volunteers were telephoned on days 3, 7 and 14 to ask if they had any expected (prompted questions) or unexpected (open-ended questions) adverse events and to discuss their compliance with the study drug. We used the combination of prompted and then open-ended questions to obtain information on possible adverse events (Materials and Methods S1).

#### End of treatment (day 28) visit

Volunteers were seen for a follow-up visit coinciding with the end of their study treatment on day 28+/−2 days as shown in Table 2. The daily symptom diary and information from telephone interviews were reviewed by the study physician and additional information was requested as needed to assess the adverse event. Remaining study capsules were returned and sent for LGG culture and colony counts. Stool samples were collected and processed as described for the baseline visit.

#### End of study (day 56) visit

On day 56 (+/−1 week) volunteers had an end-of-study visit. This visit was identical to the day 28 visit, except there was no study drug returned. Stool samples were collected and processed as described for the baseline visit.

### Outcome Measures and Measurement

#### Primary outcome

The occurrence of serious adverse events, defined as any event which resulted in death, was life-threatening, required initial or prolonged hospitalization, resulted in disability, or required intervention to prevent permanent impairment or damage to a volunteer (http://www.fda.gov/medWatch/report/DESK/advevnt.htm), or adverse events (including an abnormal laboratory or physical examination finding, symptom, or disease) defined as a new Grade II–IV toxicity using the FDA's Guidance for Industry: Toxicity Grading Scale for Healthy Adult and Adolescent Volunteers Enrolled in Preventative Vaccine Clinical Trials (Protocol S1), that were possibly/probably/highly probably related to administration of LGG.

### Measurement of the Primary Outcome

All expected and unexpected adverse events from both prompted and open-ended questions were ascertained from review of the daily symptom diary, information provided during the telephone calls, and information provided during study visits (Materials and Methods S1). The study physicians identified each adverse event on each date from any of the 3 sources. If the date that an adverse event occurred was the same in more than one source, then the adverse event was abstracted onto the case report form once. If the dates an event was reported did not match, the event was assigned to all of the days on which it was reported. Study physicians used the FDA Toxicity Grading Scales to grade event severity, but if an event was not described in the tables, severity was defined as follows:

- mild – grade 1 (an event which requires no treatment and does not interfere with the volunteer's daily activities);
- moderate – grade 2 (an event which may cause some interference with the volunteer's daily activity but does not require medical intervention);
- severe – grade 3 (an event which prevents usual daily activity and requires medical intervention); or
- potentially life threatening – grade 4 (an event which results in an ER visit or hospitalization).

For each event, study physicians reported the maximum severity during the course of the event and assessed its relationship to administration of LGG as either not related to LGG administration, remotely related (e.g. similar to events reported to have occurred before the trial), possibly related (temporally related to administration of LGG but possibly related to the volunteer's prior history), probably related (temporally related to administration of LGG and cannot be reasonably explained by the volunteer's prior history) or highly probably related (occurs immediately on ingestion of LGG). We also calculated the number of days on which each adverse event occurred during the 56 day observation period to address timing and duration of events reported once and recurrent events. Adverse events were reviewed by both study physicians during weekly meetings to ensure consistency of identification and assessment of adverse events.

An independent Data and Safety Monitoring Board, consisting of an Infectious Disease physician, a Gastroenterologist, and a Statistician monitored safety throughout the study. The DSMB met prior to subject enrollment and then approximately every 6 months until all volunteers completed the study. Our DSMB charter also indicated that the DSMB would meet more often if necessary. DSMB members were asked to review the study physician assessment of relatedness of any serious or Grade IV adverse event, should they occur.

**Secondary outcomes** included serum cytokines as well as LGG colony counts in the capsules and stool samples.

### Measurement of the secondary outcomes

#### Plasma Cytokines

Blood for cytokines was transported on ice to a centrifuge and spun at 1,200G for 10 minutes at 4°C. Plasma was aliquoted and frozen at −80°C. All specimens for cytokine analysis were placed in the −80°C freezer within 30 minutes of the blood draw. Samples were thawed once for batched analysis using the Human TH1/TH2 10-Plex Ultra-Sensitive Kit (IFN-γ, IL-1β, IL-2, IL-4, IL-5, IL-8, IL-10, IL-12p70, IL-13, and TNF-α) and the IL6 Kit both from Meso Scale Discovery (Rockville, MD) for the plasma cytokines. Cytokines were measured using electrochemiluminescence technology and sandwich immunoassay according to the manufacturer's instructions.

#### Quantification of Lactobacillus GG in stool and capsules

The procedures for obtaining LGG for colony counts in stool and capsules were as follows. 4.5 mL of sterile phosphate buffered saline was added to either 0.5 grams of stool or 0.5 grams of capsule contents and diluted to 10^−6^. 100 µL dilutions of 10^−1^ to 10^−6^ were plated in duplicate onto Lactobacillus Selection (LBS) Agar (BBL Sparks, MD) which is selective for isolation and enumeration of lactobacilli [43]. LBS plates were incubated at 37°C in an anaerobic chamber (5% CO_2_, 10% H_2_, 85% N_2_) for 48 hours. Typical white, creamy LGG colonies with a distinct buttery smell [44] were easily distinguishable from other lactobacilli on LBS agar. These colonies were counted in duplicate and the average result was reported as CFU/g of stool or capsule. Representative colonies were gram stained and LGG was preliminarily identified if there were gram positive rods in a palisade arrangement versus other Lactobacilli such as *L plantarum, L fermentum, L para para caseii* and non-LGG *L rhamnosus*. Isolates were analyzed by APIZYM (Biomerieux, Durham, NC) that distinguishes between *Lactobacillus* species based on enzymatic reactions and API CH-50 (Biomerieux, Durham, NC) that differentiates between the species based on carbohydrate reactions. *Lactobacillus para casei* and non-LGG strains of *L rhamnosus* were distinguished from LGG based on fermentation.

### Statistical considerations

#### Sample size

A sample size of 10–15 subjects was requested by the FDA for this Phase I study. We elected to study 15 subjects to have a 66% probability of detecting at least one serious adverse event and an 88% probability of detecting two or more serious adverse events, based on the binomial distribution.

#### Stopping rule

A priori, we decided that if a serious or Grade IV adverse event occurred that was judged to be probably or definitely related to LGG administration, the study would be immediately suspended pending review of all safety data, all volunteers on study would be contacted and asked to stop taking study drug, and no new volunteers would be enrolled. If this occurred, the DSMB, IRB and FDA were to be informed within 72 hours.

#### Statistical analysis

We provided descriptive statistics for demographics, medical history, prescription and over the counter medications, and dietary supplements, including means and standard deviations for continuous variables, and percentages and ranges for categorical data. We stratified hemoglobin levels by gender to account for differences in laboratory normal ranges.

We reported the number and percent of all volunteers (intent to treat) who had any adverse event (prompted or in response to an open-ended question). We also reported the number and percent of events over time. For the prompted events, we displayed the events reported by each volunteer on each study day, as well as the severity of the event in a supplemental figure (Results S1).

We performed exploratory analyses of changes in plasma cytokines as the study was not powered to detect differences over time. We calculated medians and interquartile ranges of plasma cytokines. To compare cytokines over time, we planned to use repeated measures ANOVA if the data were normally distributed or were normally distributed after a log transformation followed by Fisher's LSD post-hoc test, or a Friedman test of the data if the cytokine levels were not normally distributed before or after log transformation followed by the Wilcoxon-Nemenyi-McDonald-Thompson test for post-hoc pairwise comparisons [46].

We reported the mean and range of LGG colony counts in the capsules and used a paired t-test to evaluate whether there was any change in LGG capsule colony counts between baseline and day 28 while the LGG capsules were in the possession of the study volunteers. We reported the number and percentage of volunteers from which LGG was recovered from stool samples at baseline, day 28 and day 56 as well as the range of colony counts in the stool.

## Results

### Patient recruitment and retention

Between January 19, 2011 and August 5, 2011, 28 volunteers were screened in the Clinical Research Center for this safety and tolerability study, 15 were enrolled and all 15 completed the study (Figure 1). There were no withdrawals from the study and no adjustments to LGG dosage. Baseline characteristics of enrolled volunteers are shown in Table 3. Remote history of cancer was a common diagnosis and included 2 prior melanomas, prior breast cancer and one prior skin cancer. Remote major surgeries were also common and included 2 prior appendectomies, 2 prior cholecystectomies, 1 prior kidney surgery, and 4 prior thoracic surgeries. A total of 4 volunteers started 5 medications during the study period – proton pump inhibitors (2); NSAID (1); antibiotic (ciprofloxacin 1); and blood pressure medication (1).

**Table 3:** Baseline characteristics of enrolled volunteers (N = 15).

Demographics	Mean	SD	Range	
Age (years)	72.9	3.8	66–80	
Body Mass Index (kg/m^2^)	28.3	4.2	24.0–36.2	
	**#**	**%**		
Gender - female	10	67		
White – non Hispanic	15	100		
**Medical History**				
Hypertension	6	40		
Cancer (>2 years ago)	4	27		
Major surgery (>2 years ago)	4	27		
Osteoarthritis	3	20		
GERD 1	3	20		
Seasonal allergies	2	13		
Kidney stones	2	13		
Osteoporosis	1	7		
**Medications** 2				
Calcium, Vitamin D, other osteoporosis	10	67		
Proton Pump Inhibitors	3	20		
Aspirin or NSAIDs^3^	7	47		
Other vitamins and minerals	7	47		
Fish oil	6	40		
Antihypertensives	5	33		
Herbal supplements	5	33		
Statins	4	27		
Allergy medications	1	7		
Other^4^	7	47		
**Vital signs**	**Mean**	**SD**	**Range**	
Respiratory rate (breaths/min)	16	2	(12, 18)	
Blood pressure (mmHg)				
Systolic	125	13	(97, 141)	
Diastolic	67	11	(42, 81)	
Temperature (°C)	36.7	0.3	(36.4, 37.1)	
**Laboratory data**	**Mean**	**SD**	**Range**	**Normal Range**
Hemoglobin (gm/dL) (male)	14.8	0.8	(14.0, 15.7)	12.5–17.5
Hemoglobin (gm/dL) (female)	13.1	0.6	(12.3, 14.0)	12.0–16.0
WBC (x10^3^/µl)	7.4	1.6	(5.2, 10.7)	4.0–11.0
Neut %	58.6	6.7	(45, 70)	40–74
Lymph %	32.0	6.1	(22, 45)	14–46
Mono %	6.2	1.9	(4, 10)	4–13
EOS %	2.4	1.4	(1, 6)	0–8
Baso %	0.8	0.4	(0, 1)	0–3
Platelet count (x10^3^/µl)	244.3	51.0	(162, 346)	140–415
ALT (IU/L)	19.1	7.6	(12, 39)	0–55
AST (IU/L)	22.8	5.2	(16, 34)	0–40
Alkaline phosphatase (IU/L) (male)	65.0	15.7	(39, 80)	25–160
Alkaline phosphatase (IU/L) (female)	67.5	15.3	(45, 93)	30–100
Bilirubin total (mg/dL)	0.4	.1	(0.2, 0.6)	0.0–1.2
BUN (mg/dL)	18.3	4.6	(12, 25)	8–27
Creatinine (mg/dL)	0.9	0.2	(0.69, 1.39)	0.60–1.50
Glucose (mg/dL)	86.2	12.1	(68, 106)	65–110
Sodium (mEq/L)	140.6	1.8	(138, 143)	135–145
Potassium (mEq/L)	4.0	0.5	(3.3^5^, 5.1)	3.4–5.2

1 Gastroesophageal reflux disease.

2 During the study, 1 volunteer started taking a proton pump inhibitor on day 15 of the study, 1 volunteer started taking an antihypertensive on day 48 of the study, 1 volunteer was started on antibiotics on day 37 of the study, and 1 subject started taking an NSAID on day 7 of the study.

3 Non steroidal anti-inflammatory drugs.

4 Antidepressant medications, hormones, medications for benign prostatic hypertrophy, incontinence and sleep.

5 Low potassium, not clinically significant.

### Stability of Administered LGG

We compared colony counts of dispensed LGG capsules from the baseline visit to the colony counts in the extra capsules returned at the day 28 visit in 14 of the 15 volunteers who returned the study drug. There was no difference between the mean levels of LGG in capsules cultured at baseline (mean: 1.4×10^11^, range: 7.4×10^10^–3.4×10^11^) compared with capsules cultured at day 28 (mean: 1.4×10^11^, range: 1.3×10^10^–3.4×10^11^) (p = 0.99).

### Adverse Events

There were no serious adverse events or grade IV adverse events reported during the trial or follow-up period. The 15 study volunteers reported a total of 47 adverse events ranging from 1–7 per volunteer. Table 4 shows the adverse events occurring in the 15 volunteers (the denominator is volunteers) and Table 5 shows details of the 47 adverse events experienced by the 15 volunteers (the denominator is adverse events). All volunteers had at least one adverse event (Table 4). As shown in Table 5, 31 (70%) of the events were expected, prompted symptoms (30 of which were gastrointestinal complaints – predominantly bloating, gas, nausea) while 16 were unexpected and unrelated events. Of the 31 expected events, 28 were rated as mild and 3 as moderate. Thirty-seven (79%) were detected by volunteer self-report either on the symptom diary, telephone interview, or during the study visit. The additional 10 adverse events were 2 abnormal vital signs (a mildly elevated systolic blood pressure of 144 mm Hg and a mildly low systolic and diastolic blood pressure of 94/38 mm Hg close to this volunteer's normal blood pressure) and 8 abnormal laboratory tests in the mild range including 2 hemoglobin levels of 11.7 and 11.8 mg/dL, 2 glucose levels of 68 and 111 mg/dL, 3 blood urea nitrogen levels of 26, 27 and 30 mg/dL and 1 white blood cell count of 11.1×10^3^/µL. There was one severe, unexpected, unrelated event that occurred in one volunteer with a remote history of kidney stones who had a recurrence of kidney stones starting on study day 20. Results S1 shows the timing and duration of the *expected symptoms* only during and after receipt of LGG. The 15 volunteers had a total of 124 days of prompted symptoms during the study, 67 (54%) while consuming LGG and 57 (46%) after LGG consumption, during the follow-up period. Ninety-four percent (116) of symptoms were transient, lasting minutes and required no treatment. There were 97 days (78% of the 124) when prompted gastrointestinal symptoms occurred, 67 during LGG consumption and 30 during the follow-up period. One-third (22) of the 67 days when transient and mild prompted GI symptoms occurred were during the first week of LGG consumption.

**Table 4:** Type and timing of adverse events in enrolled volunteers (N = 15).

Study Day	1–3*		4–7*		8–14*		15–28*		29–64		Any time point	
	n	(%)	n	(%)	n	(%)	n	(%)	n	(%)	n	(%)
Volunteers reporting any AE	5	(33)	3	(20)	3	(20)	10	(67)	6	(40)	15	(100)
Volunteers reporting AEs considered treatment-related by the investigator	4	(27)	1	(7)	1	(7)	3	(20)	1	(7)	6	(40)
Volunteers reporting serious AEs considered treatment-related by the investigator	0	(0)	0	(0)	0	(0)	0	(0)	0	(0)	0	(0)

*Volunteers actively taking LGG.

**Table 5:** Expected and unexpected events during study visits, telephone calls and symptom diaries (N = 47)**.

Study Day	1–3*		4–7*		8–14*		15–28*		29–64		Any time point	
Symptoms –Study Visits, Calls, Diary	n	(%)	n	(%)	n	(%)	n	(%)	n	(%)	n	(%)
**Expected Adverse Events (Prompted)**	**5 (11)**		**4 (9)**		**6 (13)**		**7 (15)**		**9 (19)**		**31 (66)**	
***Gastrointestinal***												
Bloating	0	(0)	1	(2)	1	(2)	0	(0)	0	(0)	2	(4)
Gas	3	(6)	1	(2)	2	(4)	2	(4)	2	(4)	10	(21)
Intestinal rumbling	1	(2)	0	(0)	0	(0)	0	(0)	0	(0)	1	(2)
Diarrhea	0	(0)	0	(0)	1	(2)	1	(2)	1	(2)	3	(6)
Abdominal Cramps or Pain	0	(0)	0	(0)	0	(0)	1	(2)	0	(0)	1	(2)
Nausea	1	(2)	0	(0)	2	(4)	0	(0)	2	(4)	5	(11)
Vomiting	0	(0)	0	(0)	0	(0)	0	(0)	2	(4)	2	(4)
Loss of appetite	0	(0)	0	(0)	0	(0)	1	(2)	0	(0)	1	(2)
Heartburn	0	(0)	1	(2)	0	(0)	1	(2)	1	(2)	3	(6)
Constipation	0	(0)	1	(2)	0	(0)	1	(2)	0	(0)	2	(4)
***Other***												
Skin rash	0	(0)	0	(0)	0	(0)	0	(0)	1	(2)	1	(2)
**Unexpected Adverse Events (Spontaneously Reported)**	**0 (0)**		**1 (2)**		**1 (2)**		**2 (4)**		**2 (4)**		***6 (13)***	
Actinic keratosis	0	(0)	0	(0)	0	(0)	1	(2)	0	(0)	1	(2)
Fever	0	(0)	0	(0)	0	(0)	0	(0)	1	(2)	1	(2)
Headache	0	(0)	0	(0)	1	(2)	0	(0)	0	(0)	1	(2)
Knee pain	0	(0)	1	(2)	0	(0)	0	(0)	1	(2)	2	(4)
Kidney stones	0	(0)	0	(0)	0	(0)	1	(2)	0	(0)	1	(2)
**Study Visits**							**28** *		**56**		**Any time point**	
							**n (%)**		**n (%)**		**n (%)**	
**Unexpected Adverse Events (Physical Exam/Laboratory)**							**7 (15)**		**3 (6)**		**10 (21)**	
***Vital signs***												
High blood pressure (144/69)							0	(0)	1	(2)	1	(2)
Low blood pressure (94/38)							1	(2)	0	(0)	1	(2)
***Laboratory findings***												
Low hemoglobin							2	(4)	0	(0)	2	(4)
High White Blood Count							0	(0)	1	(2)	1	(2)
High Blood Urea Nitrogen							2	(4)	1	(2)	3	(6)
Low glucose							1	(2)	0	(0)	1	(2)
High glucose							1	(2)	0	(0)	1	(2)

*Volunteers actively taking LGG.

**39 (83%) of the 47 adverse events were graded mild, 7 were graded moderate (1 episode each of diarrhea, nausea, vomiting, fever, knee pain; 2 episodes of elevated BUN), and 1 was graded severe (1 episode of kidney stones).

### Compliance and recovery of LGG in stool cultures

For 14 of the 15 volunteers, compliance with LGG based on the number of returned capsules on the day 28 visit was 91% (range 84–100%). One volunteer lost her capsules and could not provide any information on how many capsules she consumed. LGG was recovered in the stool of 11/15 (73%) volunteers at the end of the treatment period (study day 28) although the colony counts in the stool varied widely from 1.4×10^3^ to 1.3×10^8^. No volunteer had LGG cultured from their stool at baseline or on day 56.

### Cytokine analyses

The cytokine results are shown in Table 6. In the exploratory analysis, not adjusted for multiple comparisons, only the pro-inflammatory cytokine interleukin 8 (IL-8) changed over time decreasing in association with LGG consumption (day 28) and returning towards baseline on day 56, using a Friedman test that compared all three timepoints (p = 0.038). The post-hoc Wilcoxon-Nemenyi-McDonald-Thompson test showed that IL-8 was significantly lower on day 28 versus day 56 (p = 0.029), but the differences between baseline and day 28 and baseline and day 56 IL-8 were not significantly different. There was no difference in any other pro- or anti-inflammatory cytokines over time.

**Table 6:** Median and interquartile range of cytokine production (pg/mL).

Pro Inflammatory Cytokine	Baseline	Day 28	Day 56	Friedman Test (global) p-value
IFN-γ	1.00 (0.60, 1.20)	1.00 (0.70, 1.70)	1.10 (0.80, 1.80)	0.264
IL-1β	0.25 (0.25, 0.25)	0.25 (0.25, 0.25)	0.25 (0.25, 0.25)	0.607
IL-2	0.15 (0.15, 0.40)	0.40 (0.15, 0.50)	0.30 (0.15, 0.40)	0.171
IL-5	0.40 (0.30, 0.70)	0.40 (0.30, 0.60)	0.40 (0.30, 0.60)	0.779
IL-8	9.40 (7.70, 11.80)	7.80 (6.60, 10.50)	8.80 (7.50, 12.50)	0.038*
IL-12p70	0.40 (0.15, 0.70)	0.60 (0.30, 1.20)	0.70 (0.50, 1.00)	0.105
TNF-α	4.90 (4.60, 6.30)	5.20 (4.40, 7.00)	5.10 (4.60, 6.50)	0.982
**Anti Inflammatory Cytokine**				
IL-4	0.15 (0.15, 0.15)	0.15 (0.15, 0.30)	0.15 (0.15, 0.15)	0.236
IL-10	2.30 (2.00, 2.80)	2.30 (1.70, 4.00)	2.40 (1.70, 3.50)	0.420
IL-13	1.20 (0.15, 1.70)	1.70 (0.15, 3.20)	1.20 (0.15, 1.60)	0.320

*Significantly changes over time.

### Protocol deviations

There were fourteen protocol deviations during the study period. All were reported to the Partners IRB. The deviations included the following: 4 volunteers did not return the daily symptom diary at a study visit; 3 phone calls were completed outside the windows; 2 phone calls were not completed; 2 laboratory processing errors; 1 volunteer did not return study drug at the day 28 visit; 1 volunteer had their day 56 visit outside specified window; 1 volunteer brought in the baseline stool sample 2 days after the baseline visit so was started on study drug 2 days after baseline visit. None of these minor protocol deviations resulted in withdrawal from the study. All were included in the analysis. There were no protocol deviations relating to the analysis of the cytokines.

## Discussion

Administration of the dose of 1×10^10^ CFU of LGG twice daily was well tolerated by healthy elderly volunteers during 420 patient-days of LGG treatment and 420 patient-days of follow-up without LGG treatment, with no serious adverse events or significant harms. LGG was associated with mild and transient gastrointestinal symptoms in the first week of LGG administration and these symptoms did not interfere with the daily functions of the elderly volunteers involved in this study. Most of the gastrointestinal symptoms after the first week appeared intermittently during LGG treatment and during the follow-up period without any specific pattern relating to LGG consumption. Gas was the most common symptom and was responsible for the most days with symptoms. Mild and transient gas during the first week of LGG treatment was likely related to the administration of LGG, but symptoms of gas after the first 7 days were less likely associated with LGG consumption, particularly for gas occurring after LGG was discontinued. There were no clinically relevant changes on physical examination or laboratory tests. Although safety profiles of probiotics are likely to be strain specific, it is reassuring that these results are similar to those reported by Manglat et al [45] and Oberhelman et al [47] in randomized masked trials of Lactobacillus *reuteri* DSM 17938 in younger adults. This study adds to the literature as there are few reports of use of LGG in individuals aged 65 and older.

LGG was only cultured in stool samples on day 28 (last day of treatment), indicating that colonization with LGG, if it occurs, is likely transient. This result is consistent with prior studies [48], [49] that indicate that within 4 weeks of discontinuing LGG, it can no longer be detected in stool by routine culture or polymerase chain reaction (PCR). It is possible that LGG could have fallen to levels below the limits of detection by day 56. However, there were no harms related to LGG administration between day 28 and 56 during the follow-up period after the completion of LGG.

The effect of probiotics, including LGG, on cytokines has been variable and effects in in vitro systems and animal models may not be relevant to plasma levels in humans. Cytokine responses in human studies may also be influenced by underlying illness and may not be relevant to the study of the effects in healthy individuals. The effects of probiotics are also likely to be strain specific and may vary depending on underlying illnesses or presence of inflammatory stimuli. However, some studies in model systems report either increases of anti-inflammatory cytokines or decreases of pro-inflammatory cytokines [41], [50] in association with exposure to LGG; one study specifically showed down-regulated IL-8 production [51].

Breen et al reported wide variations in cytokine levels using different kits and assay systems [52]. For this reason, great effort was placed on obtaining the plasma, promptly freezing the samples, using a single diagnostic kit for batched analysis and focusing on changes in plasma cytokines, rather than analysis of the absolute levels. It is intriguing that our preliminary study showed a decrease in plasma pro-inflammatory cytokine IL-8 at the end of LGG consumption with IL-8 levels returning to pretreatment levels on study day 56. This result needs evaluation in larger randomized trials. There was no association between any adverse event and subjects who did or did not lower their plasma IL-8 levels after consuming LGG, although the power to detect any difference was limited by the small sample size. Although not statistically significant, there was a similar magnitude of increase in plasma anti-inflammatory cytokine IL-13 on day 28. Future studies are needed to evaluate whether a potential mechanism of action of LGG in healthy adults is a reduction in systemic inflammation by decreasing pro-inflammatory cytokines and or increasing anti-inflammatory cytokines. Studies are also needed to evaluate other mechanisms of action in patients with acute and chronic illnesses.

Our study has several limitations. First, as requested by the FDA, our study used an open label design, so our volunteers knew they were receiving LGG and this may have affected their reporting of adverse events. However, there were no serious adverse events and the majority of events were mild and of short duration, suggesting that LGG is unlikely to cause significant harms in the elderly. Second, although every effort was made to encourage our volunteers to be compliant with the protocol (e.g. taking study drug, avoiding products that contain probiotics, etc.) and to record on the daily diary if study drug was not taken, we do not know whether subjects followed these instructions throughout the entire 28 day study period. However, we did include capsule counts and stool cultures for LGG which provided some evidence of compliance in at least 11 of the 15 study subjects. Third, our cytokine result suggesting that LGG is associated with decreased levels of IL-8 is exploratory and not adjusted for multiple comparisons and needs to be confirmed in additional studies.

In conclusion, this study that follows the CONSORT Statement Extension of better reporting of harms in randomized trials [38] demonstrates that *Lactobacillus rhamnosus* GG ATCC 53103 (LGG) is safe and well tolerated in healthy adults aged 65 years and older.

## Supporting Information

Checklist S1 **CONSORT Checklist.**
(PDF)

Protocol S1 **Trial Protocol.**
(PDF)

Materials and Methods S1 **Detailed materials and methods for eliciting possible harms including forms used to collec9t this information during study visits and telephone calls and the daily symptom diary.**
(PDF)

Results S1 **Timing and duration of expected adverse events.** Legend: Days on which expected (prompted) adverse events only occurred for study volunteers. Events could have occurred once or multiple times in the day and be of any duration. All events were mild, unless at least one event on a day was rated as moderate (M) or severe (S). Definitions of mild, moderate and severe are in the methods section.
(PDF)
