# Supplementary material for: No Evidence of Harms of Probiotic Lactobacillus rhamnosus GG ATCC 53103 in Healthy Elderly—A Phase I Open Label Study to Assess Safety, Tolerability and Cytokine Responses
Source: PLoS One. 2014 Dec 1;9(12):e113456. doi: 10.1371/journal.pone.0113456 (PMC4249962; doi:10.1371/journal.pone.0113456)
Supplement: Protocol S1 — Trial Protocol. (PDF) [file pone.0113456.s002.pdf]

PROBIOTICS AS AN IMMUNE ADJUVANT FOR INFLUENZA VACCINATION IN THE ELDERLY

OPEN LABEL STUDY TO EVALUATE THE SAFETY OF LACTOBACILLUS RHAMNOSUS GG ATCC 53103 (LGG) IN ELDERLY SUBJECTS

Supplier of Study Drug: Amerifit Brands, Inc.

IND Sponsor: Patricia L. Hibberd, MD, PhD

Principal Investigator: Patricia L. Hibberd, MD, PhD

Version Number: 1.1
Serial Number: 003

Date: June 6, 2011

STATEMENT OF COMPLIANCE

The study will be carried out in accordance with Good Clinical Practice (GCP) as required by the following:

- United States (US) Code of Federal Regulations (CFR) applicable to clinical studies (45 CFR Part 46, 21 CFR Part 50, 21 CFR Part 56, and 21 CFR Part 312)
- International Conference on Harmonisation (ICH) E6; 62 Federal Register 25691 (1997)
- National Institutes of Health (NIH) Clinical Terms of Award

All key personnel (all individuals responsible for the design and conduct of this study) have completed Human Subjects Protection Training.

SIGNATURE PAGE

The signature below constitutes the approval of this protocol and the attachments, and provides the necessary assurances that this trial will be conducted according to all stipulations of the protocol, including all statements regarding confidentiality, and according to local legal and regulatory requirements and applicable US federal regulations and ICH guidelines.

Principal Investigator:

Signed: ____ Date: ________________

Name
Title

TABLE OF CONTENTS

STATEMENT OF COMPLIANCE 2
SIGNATURE PAGE 3
TABLE OF CONTENTS 4
LIST OF ABBREVIATIONS 7
PROTOCOL SUMMARY 8
TIME AND EVENT SCHEDULE FOR ELDERLY SUBJECTS 11
1 KEY ROLES 12
2 INTRODUCTION AND SCIENTIFIC RATIONALE 14
2.1 BACKGROUND 14
2.1.1 Importance and Burden of Influenza 14
2.1.2 Current Strategies 14
2.1.3 Influenza Vaccine and Immunity 15
2.1.4 Immune Response to Influenza Vaccine Adjuvants, Alternative Routes and Dosing 17
2.1.5 Clinical Uses and Safety of Probiotics 18
2.1.6 Pharmacology and Toxicology Information 19
2.1.6.1 Pharmacology and mechanisms of action 19
2.1.6.2 Toxicology 21
2.1.6.1.1 Integrated Summary of Toxicological Effects of LGG in Animals and in Vitro 21
2.1.7 Previous Human Experience with Lactobacillus GG 23
2.1.7.1 Controlled Trials 23
2.1.7.2 Other Published Material Relevant to Safety 31
2.1.8 Effect of Probiotics on the Immune System 33
2.1.8.1 Effects of Probiotics on Systemic and Mucosal Humoral Immune System 33
2.1.8.2 Effects of Probiotics on the Innate Immune System 35
2.1.8.3 Effects of Prebiotics and Probiotics on the Immune Response to Influenza Vaccination 36
2.1.9 Effects of Probiotics on the Microbiota and Host Immune Response 36
2.1.10 Summary of the Background and Significance 38
3 OBJECTIVES AND OUTCOMES 39
3.1 STUDY OBJECTIVES 39
3.2 OUTCOME MEASURES 39
3.2.1 Primary Outcome Measure 39
3.2.2 Secondary Outcome Measures 39
3.2.3 Optional Sub-study Outcome Measures 39
4 STUDY DESIGN 40
5 STUDY SCREENING AND ENROLLMENT 41
5.1 SCREENING 41
5.2 SUBJECT INCLUSION CRITERIA 41
5.3 SUBJECT EXCLUSION CRITERIA 42

5.4 ENROLLMENT 44
6 STUDY DRUG 45
6.1 STUDY PRODUCT DESCRIPTION 45
6.1.1 Acquisition 45
6.1.2 Formulation, Packaging, and Labeling 45
6.1.3 Product Storage and Stability 45
6.2 DOSAGE, PREPARATION, AND ADMINISTRATION OF STUDY DRUG 45
6.3 ACCOUNTABILITY PROCEDURES FOR THE STUDY DRUG 46
6.4 ASSESSMENT OF SUBJECT COMPLIANCE WITH STUDY DRUG 46
6.5 CONCOMITANT THERAPY 46
7 STUDY SCHEDULE 48
7.1 SCREENING (VISIT DAY -31 TO DAY -1) 48
7.2 ENROLLMENT (BASELINE VISIT, DAY 0) 48
7.3 FOLLOW-UP (DAY 28 (END OF TREATMENT), DAY 56 (END OF STUDY)) 49
7.4 EARLY TERMINATION VISIT 50
8 STUDY PROCEDURES/EVALUATIONS 51
8.1 CLINICAL EVALUATIONS 51
8.2 LABORATORY EVALUATIONS 51
8.2.1 Clinical Laboratory Evaluations 51
8.2.2 Special Assays or Procedures 51
8.2.2.1 Culture of LGG 51
8.2.2.2 Microbiota 52
8.2.2.3 Immune Response Genes 52
9 ASSESSMENT OF SAFETY 54
9.1 SUBJECT EVALUATIONS 54
9.2 SUBJECT SAFETY INFORMATION 54
9.3 AVAILABILITY OF THE INVESTIGATOR 54
9.4 ADVERSE EVENTS 54
9.4.1 Definitions 54
9.4.2 Recording of Adverse Events 55
9.4.2.1 Adverse Event Severity 55
9.4.2.2 Assessing the Relationship between Study Drug and Adverse Event 56
9.4.3 Reporting of Adverse Events 57
9.5 WITHDRAWAL OF SUBJECTS 57
9.6 RESCUE MEDICATION 57
9.7 SAFETY OVERSIGHT AND STUDY TERMINATION 58
10 CLINICAL MONITORING 60
10.1 STUDY MONITORING PLAN 60
11 STATISTICAL METHODS 61
11.1 SAMPLE SIZE CONSIDERATIONS 61
11.2 INTERIM ANALYSIS 61
11.3 STATISTICAL ANALYSIS 61
11.3.1 Safety 61

11.3.2 Microbiota Richness and Diversity ... 61
11.3.3 Cytokine Production ... 61

12 SOURCE DOCUMENTS AND ACCESS TO SOURCE DATA/DOCUMENTS ... 62

13 QUALITY CONTROL AND QUALITY ASSURANCE ... 63

14 ETHICS/PROTECTION OF HUMAN SUBJECTS ... 64

14.1 ETHICAL STANDARD ... 64
14.2 INSTITUTIONAL REVIEW BOARD ... 64
14.3 INFORMED CONSENT PROCESS ... 64
14.3.1 Informed Consent/Assent Process (in Case of a Minor) ... 65
14.4 EXCLUSION OF WOMEN, MINORITIES, AND CHILDREN (SPECIAL POPULATIONS) ... 65
14.5 SUBJECT CONFIDENTIALITY ... 65

15 DATA HANDLING AND RECORD KEEPING ... 66

15.1 DATA MANAGEMENT RESPONSIBILITIES ... 66
15.2 TYPES OF DATA ... 66
15.3 STUDY RECORDS RETENTION ... 66
15.4 PROTOCOL DEVIATIONS ... 66

16 TRIAL REGISTRATION AND PUBLICATION POLICY ... 68

APPENDIX A: GUIDANCE FOR INDUSTRY ... 82

APPENDIX B: ANTIBIOTIC SUSCEPTIBILITIES ... 89

APPENDIX C: SYMPTOM DIARY ... 90

APPENDIX D: ... 91

AMENDMENT 1 SUMMARY OF CHANGES: 5/17/2010-10/15/2010 ... 91
AMENDMENT 2 SUMMARY OF CHANGES: 10/16/2010- 6/6/2011 ... 95

LIST OF ABBREVIATIONS

AE	Adverse Event
ALT	Alanine Aminotransferase
AST	Aspartate Aminotransferase
BUN	Blood Urea Nitrogen
CBC	Complete Blood Count
CFR	Code of Federal Regulations
CFU	Colony Forming Units
CLIA	Clinical Laboratory Improvement Amendments
CRC	Clinical Research Center
CRF	Case Report Form
CRP	C-reactive protein
DSMB	Data and Safety Monitoring Board
FDA	Food and Drug Administration
GCP	Good Clinical Practice
GI	Gastrointestinal
HAI	Hemagglutinin inhibition
HIPAA	Health Insurance Portability and Accountability Act
ICH	International Conference on Harmonisation
ILI	Influenza like illness
IND	Investigational New Drug Application
IRB	Institutional Review Board
IV	Intravenous
LAIV	Live attenuated influenza vaccine
LDI	Laboratory documented illness
LGG	Lactobacillus rhamnosus GG, ATCC 53103
LLN	Lower Limit of Normal
MN	Microneutralizaton
N	Number (typically refers to subjects)
NIH	National Institutes of Health
PI	Principal Investigator
RBCs	Red Blood Cells
SAE	Serious Adverse Event
SOP	Standard Operating Procedure
TIV	Trivalent Influenza Vaccine
ULN	Upper Limit of Normal
WBC	White Blood Cell

PROTOCOL SUMMARY

**Title:**	Probiotics as an immune adjuvant for influenza vaccination in the elderly<br><br>Stage I - open label study to evaluate the safety of Lactobacillus rhamnosus GG ATCC 53103 (LGG) in elderly subjects
**Phase:**	I
**Population:**	10-15 elderly subjects, ages 65-80 years
**Subject Participation Duration:**	Approximately 3 months (includes screening visit, baseline visit and follow-up through 2 months).
**Description of Agent or Intervention:**	*Lactobacillus rhamnosus* GG ATCC 53103 (LGG) capsules containing 1 x 10^{10} CFU
**Dosage and Administration:**	LGG capsules will be administered orally twice a day for 28 days – total daily dose 2 x 10^{10} CFU
**Objectives:**	
**Primary:**	Assess the safety and tolerability of 2 x 10^{10} CFU LGG administered orally to elderly subjects for 28 days.
**Secondary:**	Evaluate the richness and microbial diversity in nasopharyngeal and stool specimens using pyrosequencing.
**Optional Sub-study**:	Compare cytokine production in response to bacterial stimulation by following the kinetics of mRNA expression of pro and anti-inflammatory genes and different signaling pathways, in relation to changes in stool *Bifidobacterium* and *Lactobacillus spp*.
**Study Design:**	Open label trial in elderly subjects. Eligible subjects will be recruited using IRB-approved procedures and screened as outpatients in the Clinical Research Center (CRC) of Tufts Medical Center or Massachusetts General Hospital. Enrolled subjects will take 1 LGG capsule orally, twice a day, for 28 days, as outpatients. Subjects will have visits in the CRC at baseline, Day 28 and Day 56. During each

	visit, the subject diary, interim history, potential adverse effects and concomitant medications will be reviewed and vital signs and a physical examination will be performed. Routine blood and urine tests will be performed as specified during visits and nasopharyngeal and stool samples will be collected. Subjects will also be contacted by telephone on days 3, 7, and 14, to determine if any adverse events have occurred. Those participating in the sub-study will have extra blood drawn for DNA and RNA extraction.
**Safety Evaluations:**	Safety will be assessed by interim history, review of subject diaries, adverse event questionnaires administered during study visits and on telephone calls, vital signs, physical examinations and laboratory tests.

Description of Study Design:

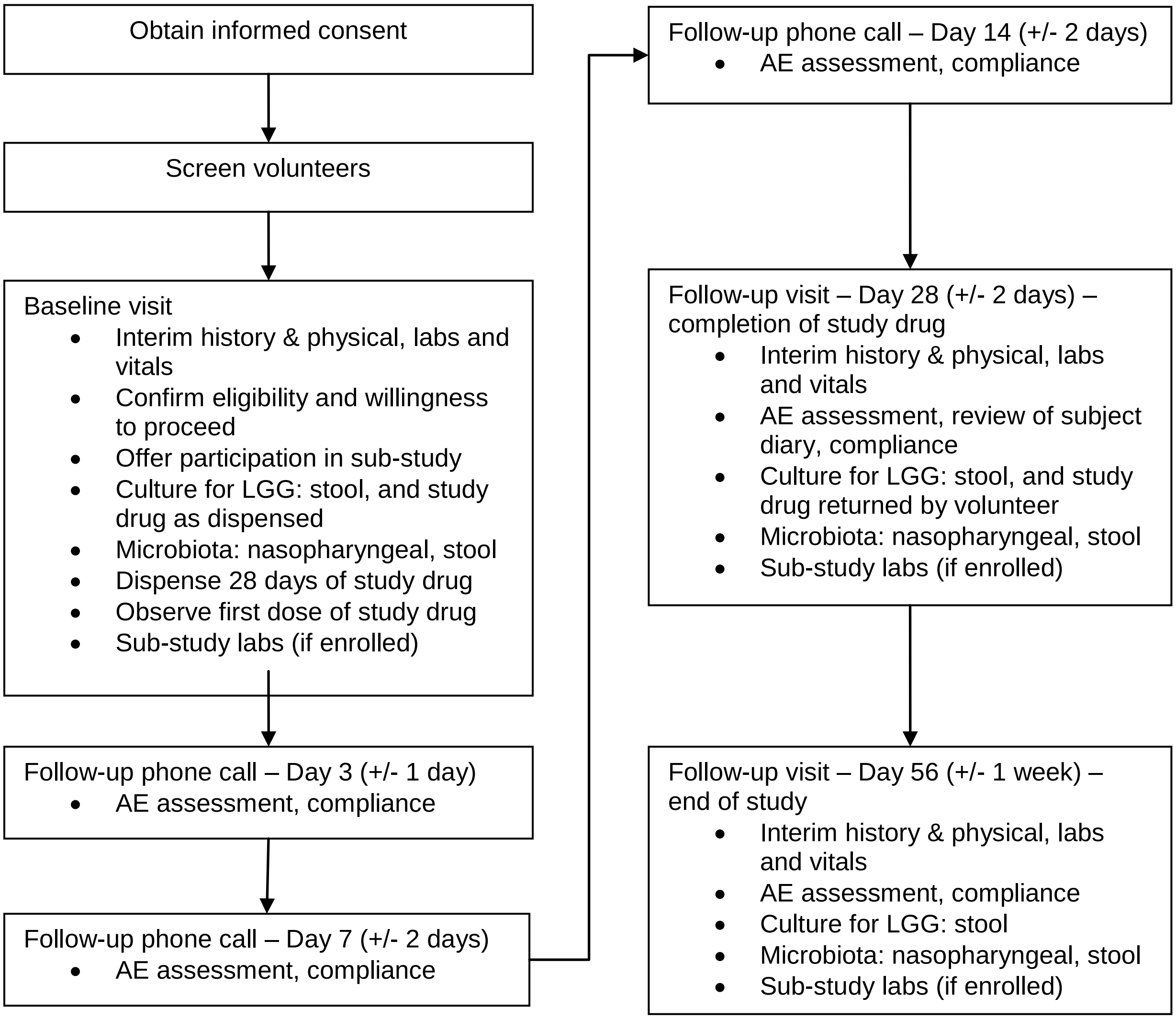

TIME AND EVENT SCHEDULE FOR ELDERLY SUBJECTS

Measurement	Screening Day -31 to Day -1	Baseline Day 0	End of Therapy Day 28	End of Study Day 56
Informed consent / HIPAA authorization	X			
Inclusion/exclusion criteria	X	X		
Demographics	X			
History or interval history (and yogurt consumption)	X	X	X	X
Vital signs, physical examination	X	X	X	X
CBC, serum chemistries, liver function tests	X	X	X	X
Drug and alcohol screening	X	Alcohol		
HIV antibody, HCV antibody Hepatitis B Surface antigen	X			
Concomitant therapy	X	X	X	X
Study treatment		X	Twice daily x 28 days	
Study drug counts			X	
Stool culture for LGG – if positive, PFGE/PCR compared to administered LGG		X	X	X
Stool and nasopharyngeal samples for microbiota		X	X	X
Study drug colony counts		X	X	
Sub-study blood (DNA, RNA, cytokines)		X	X	X
Adverse events – questionnaire, self report and subject diary		X	X	X

1 KEY ROLES

For questions regarding this protocol, contact Patricia L. Hibberd, MD, PhD, Physician, Massachusetts General Hospital; Phone 617-643-8683; Fax 617-726-1886; phibberd@partners.org and Tufts Medical Center; Phone 617-636-5143; Fax 617-636-1580; phibberd@tuftsmedicalcenter.org. Dr Hibberd is Principal Investigator of the study at both institutions.

Individuals:

Principal Investigator:

Patricia L. Hibberd, MD, PhD
Massachusetts General Hospital
50 Staniford Street, Suite 401
Boston MA 02114
Phone : 617-643-8683
Fax : 617-726-1886
Cell : 781-290-7226
Email : phibberd@partners.org

and

Patricia L. Hibberd, MD, PhD
Tufts Medical Center
800 Washington St. Box 041
Boston, MA 02111
Phone: 617-636-5143
Fax: 617-636-1580
Cell : 781-290-7226
Email: phibberd@tuftsmedicalcenter.org

Sub-Investigators:
Lisa Davidson, MD
Tufts Medical Center
800 Washington St, Box 238
Boston, MA 02111
Phone: 617-636-2867
Email: ldavidson1@tuftsmedicalcenter.org

Protocol Manager:
Christine Botelho, MPH
50 Staniford Street, Suite 401
Boston MA 02114
Phone : 617-643-8683

Email : cbotelho@partners.org

Statistician and Data Manager:
Anne-Maria Fiorino, MS
50 Staniford Street, Suite 401
Boston MA 02114
Phone : 617-643-8683
Email: afiorino@partners.org

Study Coordinator:
Irina Andreyeva
50 Staniford Street, Suite 401
Boston MA 02114
Phone : 617-643-8683
Email: iandreyeva@partners.org

Research Coordinator:
Elyse Goveia
Tufts Medical Center
800 Washington St. Box 041
Boston, MA 02111
Phone: 617-636-5143
Email: egoveia@tuftsmedicalcenter.org

2 INTRODUCTION AND SCIENTIFIC RATIONALE

2.1 Background

2.1.1 Importance and Burden of Influenza

Influenza remains a major cause of morbidity and mortality in the United States. Illness occurs in 10-20% of the population each year [1]. Adults aged $\geq$ 65, young children, and people with chronic medical conditions continue to bear the brunt of the disease because they are at higher risk for complications, hospitalizations, and death from influenza [2]. The majority of influenza related deaths ($\geq$90%) occur among the elderly [2, 3] and influenza associated mortality appears to be increasing (estimated 19,000 influenza-associated deaths per influenza season from 1976-1990 and 36,000 deaths per season from 1990-1999) [3, 4]. From 1970-1995, of the estimated 3 million excess hospitalizations associated with influenza, rates were highest in the elderly (174/100,000) vs. the rest of the population (49/100,000) [5]. This increase may be due to an increase in the elderly population or the number of influenza seasons in which influenza A (H3N2) predominates [2-5]. Influenza-related deaths can result from pneumonia, exacerbations of cardiopulmonary conditions and other chronic diseases. Thompson et al. estimated rates of influenza-associated pulmonary and circulatory deaths/100,000 persons were 0.4-0.6 among persons 0-49 years, 7.5 among persons 50-64 years, and 98.3 among persons $\geq$65 years [3].

2.1.2 Current Strategies

Influenza vaccination is the primary means of preventing influenza infection. Two types of vaccine are available - inactivated trivalent influenza vaccine (TIV) and live attenuated influenza vaccine (LAIV). The Advisory Committee on Immunization Practices currently recommends annual vaccination with TIV in persons aged $\geq$50 years, children aged 6-23 months, pregnant women, and persons of any age with chronic medical conditions [6]. LAIV is recommended for healthy children and non-pregnant adults aged 2-49 years. TIV vaccination in the elderly results in reductions in hospitalization, morbidity, and mortality [7]. Annual influenza vaccination has been shown to be associated with a reduction in all-cause mortality in community dwelling elderly persons [8]. In addition, the CDC also recommends that health-care workers and household contacts who have frequent contact with persons at high risk should also be vaccinated. Although young children and the elderly suffer the greatest morbidity and mortality, exposure of their caregivers results in a high burden of health care expenditures. Influenza vaccination reduces both direct medical costs, such as physician visits and antibiotics uses, and indirect costs such as work absenteeism [9, 10].

While studies in children have demonstrated superior or equivalent efficacy between LAIV and TIV in children, recent studies have not consistently demonstrated the same robust results in healthy adults. One randomized controlled trial comparing LAIV and TIV published in 1994 found that LAIV was equally efficacious to TIV in reducing infection and morbidity due to influenza [11]. In a meta

analysis comparing 18 randomized trials involving a total of 5000 vaccines [12], the two vaccines were found to have similar efficacy in preventing influenza infection and similar rates of adverse reactions, but this conclusion was not based on studies directly comparing the two vaccines. However, Monto et al [13] reported sub-optimal protection against laboratory confirmed symptomatic influenza following administration of LAIV compared with TIV for the 2007-2008 influenza season. Absolute efficacy was 68% in those subjects receiving TIV vs. 36% in those receiving LAIV. Similarly, in a study evaluating prevention of laboratory-confirmed symptomatic illnesses from influenza in healthy adults during the 2004-2005 season in which most circulating viruses were dissimilar to those included in the vaccine, TIV was effective, while LAIV was less efficacious [14].

Concerns about vaccine safety and sub-optimal efficacy in the elderly, people with co-morbid conditions, and the immunosuppressed continue to spur the search for ways to boost immune response after administration of both types of vaccine. One recently published study in the elderly did find LAIV to be protective against culture confirmed influenza in adults aged 60 and over [15]. An alternative promising strategy is co-administration of LAIV and TIV in the elderly. Several placebo controlled studies suggest that the combination may be safe and immunogenic [10, 16-24]. In one trial to evaluate safety and efficacy of LAIV in combination with TIV in patients age $\geq$ 65 with chronic diseases, LAIV was well tolerated compared with an intranasal placebo [20]. In another double blind randomized study of 532 nursing home residents over a 3 year period, subjects who received both TIV and LAIV had significantly lower rates of laboratory documented influenza than those receiving TIV and placebo [23]. In a third large study of older adults with COPD, LAIV and TIV were found to be equally efficacious and well-tolerated [18]. A fourth study of nursing home residents and elderly adults in St. Petersburg, found that the combination vaccine strategy was well tolerated and associated with greater efficacy than TIV alone [21]. However, the majority of elderly participants in all published trials had suboptimal responses to the combination of LAIV and TIV, or LAIV alone, indicating that there is still room for improvement.

2.1.3 Influenza Vaccine and Immunity

Immunity to influenza infection is induced by antibody responses to viral surface antigens hemagglutinin (HA) and neuraminidase (NA). The influenza virus is constantly undergoing antigenic drift in these two proteins. The efficacy of influenza vaccination depends on the degree of concordance between the virus strains in the vaccine and those being spread in the population at large. Antibody induced immunity by vaccination in one season is unlikely to be of benefit in the following season.

Both systemic and local immune responses protect against infection with the influenza virus. Since the main portal-of-entry for the influenza virus is mucosal tissue, the mucosal immune system is the key first line of defense against infection. The mucosal immune response is primarily reflected by the local production of secretory IgA that can be detected in nasal washings. Levels of IgM and IgG can also be detected in nasal secretions during primary infection. In subjects previously exposed to influenza, the local and systemic IgA response predominates. Brokstad et al found that even in the absence of influenza exposure, much higher levels of influenza specific antibody secreting cells are found in nasal mucosa than in blood [25]. One of the advantages of the LAIV is that the vaccine

strains replicate in the respiratory epithelium of the nasal mucosa, stimulating nasal IgA and inducing the local humoral immune response. LAIV induces peak of serum IgA and IgM two weeks after immunization and a peak IgG response 4-12 weeks after immunization [26]. Following LAIV administration, mucosal antibodies detected in nasal wash specimens appear to have a long half-life and in previously immunized children may persist up to a year [27]. Nasal wash IgA levels were a stronger predictor of protection against influenza than serum HAI antibody titers [27]. In a study of the local and systemic immune response in nursing home elderly following either intranasal or intramuscular inactivated vaccine, the intranasal vaccine was found to be more effective at inducing a mucosal IgA response [28, 29].

Serum production of anti-influenza IgG reflects the systemic humoral response to either LAIV or TIV. This is most commonly measured by the serum hemagglutination inhibition (HAI) test, which measures the ability of serum antibodies to inhibit influenza hemagglutinin (HA)-induced agglutination of avian (chicken or turkey) red blood cells [30]. The systemic immune response to TIV can be detected within 7 days and most commonly peaks at 10-14 days [31]. The systemic acute immune response is characterized by a rise in serum IgA and IgM levels within the first two weeks, followed by IgG levels that persist for up to 6 weeks [31-33]. In those previously exposed to influenza vaccine, serum IgG and IgA are the main indicators of immune response [33]. To date, there are no published data on a head-to-head comparison of the efficacy of the two vaccines. TIV produces higher levels of serum anti-HA IgG and IgA antibodies, while LAIV induces higher levels of nasal wash IgA [12, 34, 35]. Both TIV and LAIV have similar rates of systemic and local immune responses when compared with placebo vaccine [34]. The LAIV resulted in lower levels of serum HAI antibody responses but higher levels of local IgA antibodies in nasal washings[34].

While humoral immune responses to influenza virus are responsible for the resistance to infection, cell mediated immunity is important for the clearance of the virus, reduction of the severity of the disease, and recovery from infection [17, 36]. In *in-vitro* studies, the cytotoxic T lymphocyte (CTL) response to influenza correlates with decreased viral shedding [37]. The CTL response is at least partially dependent on CD8+ T cells that are specific for HA as well as the internal proteins M, NP or B2 [38]. Th1 cytokines are also important for the cell mediated immune response to influenza, in particular interferon gamma that appears to be □isimportant for memory T cell responses to influenza in mice and humans [39, 40]. A recent study by Guthrie et al. demonstrated that tonsillar and peripheral blood mononuclear cells proliferate strongly in response to influenza antigens, suggesting that naturally acquired immunity exists within both the mucosal and systemic compartments [41]. In addition, influenza vaccination induced significantly stronger T cell responses in both the palatine tonsils and blood, in addition to increasing titers of anti-influenza antibodies in serum and saliva [41].The measurement of the CTL response to influenza has been used to study immune responses in different human populations and different vaccine preparations [17, 19, 42]. Another recently used approach to measure cell mediated immune response to influenza is to measure the proliferation of peripheral blood monocytes and cytokine production in response to influenza antigens [43, 44].

Immune responses in the elderly. Both B-cell and T cell function, particularly T cell generation and TCR diversity are down-regulated in the elderly [45 46, 47] Influenza-specific immune responses are also known to be reduced with aging [47]. Peripheral blood monocytes activated with influenza antigens show an age-related decline in the elderly compared with young adults. In addition, elderly subjects show a decreased and delayed type 1 T cell response to influenza, resulting in a reduced IgG1 subtype and total antibody response [40]. Immunization in the elderly population primarily results in induction of memory T and B cell responses and not naïve T cell responses. Since a naïve T cell response is required to protect against new epitopes by newly evolved strains of the virus, this may be the reason for failure of the immune response to vaccination in the elderly. McElhaney et al, recently studied granzyme B levels in virus stimulated peripheral blood mononuclear cells and found that older adults with laboratory documented influenza had lower levels of granzyme B than those who did not develop influenza, while both groups had similar antibody titers, raising questions about the best way to predict vaccine efficacy in the elderly [48].

Immune Response to Combination Influenza Vaccination. Despite the promise of enhanced mucosal immunity to influenza following LAIV, concerns about vaccine safety and sub-optimal efficacy in the elderly, people with co-morbid conditions and the immunosuppressed have resulted in a timely search for ways to boost the immune response in recipients of both the LAIV and TIV. Older persons are well-known to develop lower post-vaccination antibody titers than younger individuals [6, 38, 49, 50]. Subjects who received both TIV and LAIV have an improved immunologic response to influenza vaccination s[17, 22-24, 51]. In a study of fifty elderly nursing home residents who received TIV and either nasal LAIV or placebo, there were significant increases in anti–HI and anti-H3 IgA antibodies in nasal wash specimens from patients who received LAIV vs. placebo [17]. In two studies of older chronically ill adults, patients who received both LAIV and TIV had higher and sustained nasal wash IgA anti-influenza HA levels compared with those who received TIV alone [17-19]. In another study of nursing home residents who received LAIV, TIV, or a combination, only individuals who received LAIV (alone or in combination) had a rise in virus-specific nasal IgA. CTL activity is enhanced with the combination vaccines in the elderly [16, 23]. Sasaki et al recently reported on administration of TIV then either LAIV or TIV in the next influenza season. Subjects who had previously received TIV had higher prevaccine HAI titers, but lower HAI response to new LAIV or TIV and a lower effector B cell response to new TIV but not new LAIV or TIV [52].

2.1.4 Immune Response to Influenza Vaccine Adjuvants, Alternative Routes and Dosing

Given that many elderly and those with chronic illness or immunocompromised may not be able to safely receive LAIV (with or without TIV), attention has also focused alternative dosing regimens or intranasal administration of whole trivalent influenza vaccine. Several recent studies found that reduced dose intradermal or intramuscular injection of inactivated vaccine resulted in a similar antibody response to intramuscular injection with a full dose of vaccine [53-55]. One of these studies found that antibody responses in patients over age 60 were suboptimal compared with antibody responses in younger subjects. Chi et al found that administration of 60% of the TIV dose either intradermally or intramuscullary elicited antibody responses similar to intramuscular full dose TIV in

healthy adults aged 65 years and older. No data on efficacy of these reduced dose regimens has been published to date. Preliminary data using administration of whole trivalent influenza vaccine suggest that this approach vs. intramuscular vaccine are equally efficacious in inducing protective levels of antibodies, but the nasal route resulted in a greater mucosal IgA response in elderly patients [29, 56].

High dose influenza vaccines have also been evaluated in adults over age 65 years. Intramuscular administration of 60µg of the 3 antigens in the 2006 TIV resulted in higher levels of both influenza A strains in the vaccine and similar levels to the influenza B strain compared with the standard 15 µg dose of each antigen, without increased adverse events [57]. Another high dose influenza vaccine (Agriflu) with three times the standard dose was also recently approved by the FDA.

An alternative approach is to boost the immune response to influenza vaccination by administering an adjuvant at the time of vaccination. Cooper et al. used oligodeoxynucleotides containing immunostimulatory motifs as a vaccine adjuvant to TIV [43]. In a placebo, controlled trial, the adjuvant was safe and well tolerated. The adjuvant-vaccine combination did not increase HAI or ELISA titers in this small study, but there was a trend to increased titers in those with pre-existing immunity to one influenza strain. The greatest effect of this vaccine adjuvant was observed in the group receiving a low dose of TIV, raising the possibility that an immune boosting response may allow for a reduction in vaccine dose. Other adjuvant strategies focusing on the elderly have included an IL-2 supplemented liposomal influenza vaccine, a diphtheria toxoid conjugate vaccine, and a conjugate vaccine with heat-labile enterotoxin from *E.coli* [58-61].

MF59 is another vaccine adjuvant (submicron oil in water emulsion of 5% squalene, 0.5% Tween 80 and 5% Span 85) that has been safely used in Human Immunodeficiency Virus and Herpes simplex sub-unit vaccine trials. In a recent meta-analysis, MF59 adjuvanted influenza vaccine resulted in greater immunogenicity than non adjuvanted vaccine especially in those who had not been previously immunized [62]. Studies of MF59 adjuvanted influenza vaccine in healthy adults also resulted in higher HAI titers [63]. However, Boyce et al recently evaluated MF59 adjuvanted intranasal vaccine in an open label safety study finding similar mucosal IgA responses after administration of both adjuvanted and non-adjuvanted intranasal vaccine [64]. Another meta-analysis specifically looking at immunogenicity and safety of MF59 adjuvanted influenza vaccine in the elderly found increases in geometric mean titers to all 3 strains of influenza vaccine when compared with traditional TIV[65], a result that was also confirmed by Sindoni et al [66]. However, elderly patients had more adverse reactions to the adjuvanted vaccine. A recent study of MF59 adjuvanted H5N3 vaccine showed improved antibody responses and seroconversion when compared with non-adjuvanted vaccine [67]. Currently, the MF59-adjuvanted influenza vaccine is not licensed for use in the United States.

2.1.5 Clinical Uses and Safety of Probiotics

Probiotics are living microorganisms that exert health benefits beyond inherent nutrition [68]. Biotherapeutic agent is an alternative term that is used to describe microorganisms that have antagonistic properties toward pathogenic bacteria [69]. There are numerous commercially available

probiotics, both in lyophilized form or fermented food products. Several probiotics and biotherapeutic agents such as *Lactobacillus spp* have been studied for the treatment of antibiotic associated diarrhea, infantile diarrhea, traveler's diarrhea, urinary tract infections, and vaginal infection [68, 70-72].

2.1.6 Pharmacology and Toxicology Information

2.1.6.1 Pharmacology and mechanisms of action

Since effects of Lactobacilli vary by strain, we have focused our synthesis of the literature on LGG, ATCC 53103. The precise pharmacological effects and mechanisms of action are not known but effects of LGG and other probiotics are thought due to the following. First, the presence of LGG leads to colonization resistance, the prevention of adhesion to and colonization of the intestine by other pathogens, as well as the impedance of translocation of intestinal bacteria across the bowel wall. Second, LGG causes immune modulation, activating the body's innate immune response to fight infection or down-regulating the immune response in hypersensitivity. Third, LGG has direct antimicrobial effects resulting in production of locally acting substances, which kill or inhibit the growth of pathogenic organisms.

Colonization resistance: The presence of LGG in the intestinal tract serves to decrease both the adhesion of pathogenic organisms to the epithelium and colonization by those organisms. In mice infected with *Salmonella typhimurium* and given LGG versus placebo, LGG led to decreased *Salmonella* levels and prolonged life [73]. A similar effect has been shown with *Clostridium difficile* in hamsters [74]. Inhibition of adhesion of other organisms by LGG has been shown in gastrointestinal epithelium as well as uroepithelium [75]. Studies of Lactobacilli in tissue culture systems show inhibition of adherence of *Escherichia coli*, *Klebsiella,* and *Pseudomonas* in uroepithelial cells [75]. LGG strengthens the barrier mechanisms of the intestinal mucosa either cellularly or by its effect on the microecology. LGG itself does not invade the epithelium [76]. In suckling rats, LGG strengthens the intestinal mucosal barrier by decreasing permeability of the intestine to macromolecules, and increasing intestinal antibody production [77]. A similar decrease in intestinal permeability occurs in rats pretreated with LGG before rotavirus infection. In our laboratory using a lethal irradiation mouse model designed to develop bacteremia with intestinal flora, animals treated with LGG had prolonged survival. Lactobacilli were never isolated from the blood of these bacteremic mice despite ingestion of large quantities of LGG [78]. Similar results have been seen with *Salmonella* and *Escherichia coli* [73, 79]. Inhibition of adhesion of other organisms by LGG has been shown in gastrointestinal epithelium as well as uroepithelium [75] and in the vaginal epithelium [71, 80]. Mattar et al evaluated MUC-2 mucin gene expression in a Caco-2 cell-culture model incubated with LGG versus control media [81]. LGG was associated with increased MUC-2 expression possibly by binding to specific receptor sites on the enterocytes. This may explain previously observed inhibition of bacterial translocation [82].

Immune modulation:

Effect on the Gut Epithelium – LGG was associated with an increase in intestinal villi in germ-free rats [83], improvement in an induced gut permeability disorder in suckling rats [77], and prevention of cytokine-induced apoptosis in mouse and human colon cells [84], suggesting a possible effect of LGG on inflammatory conditions induced by microbial pathogens.

Effect on the Innate Immune System – Most of the studies of LGG on the innate immune system have been conducted on monocytes or macrophages *in vitro*. LGG results in the production of interferon gamma, IL-12, and IL-18 (the latter two are monocyte specific), weak production of IL-10, and no production of IL-4 [85]. LGG cell wall components activate human monocyte transcription factors involved in cytokine signaling both directly leading to NF-κ B activation and indirectly via STAT activation [86]. A recent study found a striking difference between LGG-stimulated dendritic cells that resulted in only moderate expression of co-stimulatory molecules, low production of TNF-α, and CCL20, and no production of IL-2, IL-12, IL-23, and IL-27 compared with a vigorous Th1-type response to stimulation with pathogenic *Streptococcus pyogenes* [87], suggesting differential responses to pathogenic and nonpathogenic gram positive bacteria. Similar differential modulation of dendritic cells was reported by Braat et al comparing responses to *Klebsiella pneumoniae* and *Lactobacillus rhamnosus* [88]. Korhonen et al reported that lipotechoic acid appeared to be the active component of LGG (in the presence of interferon gamma) that stimulated nitric oxide production in a macrophage cell line J775 [89].

Effect on the Adaptive Immune System – The evidence in support of the effect of LGG in B lymphocytes has mostly come from human trials of enhanced immunogenicity of the oral rotavirus vaccine in those receiving LGG [90] and on increased *Salmonella* specific IgA levels in those who received the oral *Salmonella* vaccine with LGG [91]. Thus, LGG appears to enhance IgA response to antigens concurrently delivered to the GI mucosa. Both *in vivo* and *in vitro* studies indirectly suggest that LGG attenuates the type 2 immune response. Pochard et al studied the effect of various lactobacilli including *Lactobacillus rhamnosus* on the cytokines secreted by peripheral blood mononuclear cells stimulated with Staphylococcal enterotoxin A or *D pteronyssinus* [92]. Preincubation with lactobacilli inhibited the production of IL-4 and IL-5 (Th2 cytokines), probably by an inhibitory effect of lactobacilli on IL-1 and interferon gamma, because neutralization of these cytokines restored IL-4 production.

Direct antimicrobial effects:

Production of Hydrogen Peroxide – The production of hydrogen peroxide may be a non-specific normal vaginal antimicrobial defense mechanism. Vaginal strains of *Lactobacillus (L crispatus and L paracasei)* that produce above a threshold amount of hydrogen peroxide also inhibited growth of *S aureus in vitro* [93].

Production of acids – LGG produces acetic and lactic acid, lowering the pH which results in inhibition of growth of a wide range of bacteria, including but not limited to *E coli, Streptococcus, Pseudomonas, Salmonella, Bacteroides, Clostridium, Bifidobacterium,* and *S aureus* [73, 94-96].

Production of biosurfactants – *Lactobacillus fermentum* RC-14 inhibits *S aureus* infections of surgical implants in rats [97-100]. Biosurfactants have also been recovered from *L rhamnosus* [101].

Production of antimicrobial substances – Four classes of bacteriocins are produced by lactobacilli. They have a relatively narrow spectrum of activity and are toxic to closely related bacteria, including *Lactococcus*, *Streptococcus, Staphylococcus, Listeria*, and *Mycobacteria*. Bacteriocins either target the cytoplasmic membrane or essential enzymes of susceptible bacteria. LGG specifically secretes an inhibitory compound that does not have characteristics of bacteriocins and is neither lactic nor acetic acid. This compound has a low molecular weight, is heat stable, and is active against a wide range of bacteria, including *S aureus* [102].

<u>Information on absorption, distribution, metabolism and excretion</u>: LGG is not absorbed or distributed, except in rare case reports reported below regarding safety. Little is known about its metabolism. When ingested orally, LGG adheres to the intestinal epithelium, colonizes the gut, and becomes one of the principal bacteria in the fecal flora. Because it is particularly resistant to acid and bile, LGG survives in the intestinal tract better than other Lactobacilli and can be recovered from stool, days to weeks after cessation of administration [103]. LGG can be shown to adhere to intestinal epithelium in cell culture [104] as well as by biopsy of colonic mucosa in patients [105].

2.1.6.2 Toxicology

2.1.6.1.1 Integrated Summary of Toxicological Effects of LGG in Animals and in Vitro

There are limited formal toxicology studies of LGG in animals, particularly relating to acute, subacute, and chronic toxicity, or effects on reproduction and the developing fetus. In one study, male adult Swiss mice were fed LGG in graduated doses of 1, 2, 4, or 6 g of test bacteria/bodyweight, and compared to control animals fed distilled water. There were no treatment-related deaths and no evidence of treatment-related toxicity, although the mice fed bacteria showed anorexia and listlessness in the initial 24 hours post-dosing [106]. The authors concluded that the amount of LGG ingested by the mice would be equivalent to more than 420 grams of bacteria for a 70-kg human [107]. However, there are numerous studies evaluating the safety of LGG *in vitro* and in animal models. These studies address risk of translocation; risk of transfer of antimicrobial resistance; and risk of gastrointestinal and immunologic toxicity.

Risk of Translocation of LGG: Translocation by intestinal bacteria is facilitated by numerous factors including intestinal mucosal injury, immunodeficiency, gut prematurity, and abnormal bacterial flora (e.g. overgrowth) [108, 109], as well as adherence of the bacteria to the mucosal surface [110]. Ouwehand et al [111] studied adhesion of *Lactobacillus* spp to human intestinal mucosa of patients with diverticulitis, rectal carcinoma, and irritable bowel disease (IBD) versus healthy normal human colonic tissue. Adherence to immobilized colonic mucosa and mucus was measured using radio-labeled bacteria. All strains were more adherent to mucus than whole tissue. *Lactobacillus rhamnosus* GG adhered significantly less to control and diverticulosis tissue than rectal carcinoma or IBD tissue, but had significantly greater adherence to intestinal mucus from all the tissue types than other types of *Lactobacillus* that were tested. However, it is not clear whether the *in vitro* finding of greater adherence to mucus predicts ability of the bacteria to translocate. More recently, Vesterlund et al compared 52 invasive clinical *Lactobacillus* spp isolates, with similar bacteria (all

Lactobacilli) isolated from 15 probiotics and 44 fecal samples [112]. In this study, the authors speculate that translocation in the clinical isolates versus probiotic strains could have been facilitated by their increased ability to adhere to mucus.

Risk of Immunologic Toxicity: Theoretical concerns have also been raised about mucus degradation and platelet aggregating activity. However, probiotics do not degrade intestinal mucus based on both *in vitro* and in studies of gnotobiotic rats [113]. Since there are several case reports of bacterial endocarditis in patients receiving probiotics, concerns have been raised about whether probiotics have platelet aggregating activity [114]. Harty et al [115, 116] recently examined the aggregation properties of 10 *Lactobacillus* strains from patients with infective endocarditis (IE): 5 *L rhamnosus* and 5 *L paracasei*. These strains were then compared to oral strains. Aggregation of platelets occurred with all *L rhamnosus* IE strains and eleven of fourteen strains from other *Lactobacillus species*. Inhibition of aggregation with the peptide arginine-glycine-aspartic acid-serine (RGDS) was consistent with involvement of fibronectin and/or fibrinogen.

Risk of Transfer of Antimicrobial Resistance: Ideally, probiotic strains would not harbor antimicrobial resistance genes on transmissible elements that are capable of transfer to pathogenic or opportunistic pathogenic bacteria [117, 118] but many existing probiotic strains already do [119-122]. Antibiotic resistance can be located on mobile genetic elements such as plasmids or transposons (where transfer between bacteria is easy), or on the bacterial chromosome (where transfer is difficult, at least for lactobacilli [120]. Plasmids are common in most of the probiotic bacteria, but not all antimicrobial resistance is harbored on plasmids. Ammor et al recently found resistance genes in several lactic acid bacteria and Bifidobacterium – specifically, resistance to tetracycline [tet(M), tet(W), tet(O) and tet(O/W)], erythromycin and clindamycin [erm(B)], and streptomycin [aph(E) and sat(3)] [123]. Most of the resistance determinants were located on the bacterial chromosome – except for tet(M), which was identified on plasmids in *Lactococcus lactis*. Given the increasing clinical importance of invasive infections with vancomycin-resistant enterococci and threat of emergence of vancomycin-resistant *Staphylococcus aureus*, attention has been focused on the potential for transfer of vancomycin resistance to and from probiotic bacteria. Many strains of lactobacilli are naturally resistant to vancomycin. In the Lactobacillus strains studied to date, the vancomycin resistance genes appear to be chromosomally located and are not easily transferable to other genera [124, 125]. Mater et al demonstrated transfer of vancomycin resistance (VanA cluster) from Enterococcus to a commercial strain *of Lactobacillus acidophilus*, both *in vitro* and in the gut of mice [126, 127]. Since the mice were colonized with human microbiota and this transfer occurred in the absence of selective pressure from antibiotics, further investigation of the potential is urgently needed. However, since many lactobacilli are already intrinsically resistant to vancomycin, there would be no selective advantage to harboring additional vancomycin-resistant plasmids.

Risk of Gastrointestinal Toxicity: Theoretical concerns have been raised about whether probiotics produce gastrointestinal toxicity as a result of their enzymatic activity [128]. Attention has particularly focused on bile salt deconjugase activity that could result in malabsorption and increased risk for colon cancer by acting on mucus-producing cells and stimulating proliferation [129]. However, there is no evidence in support of this concern.

Summary: The risks of translocation and invasive disease caused by LGG (ATCC 53103) appear to be low, although there are theoretical risks that LGG may degrade intestinal mucus and could cause platelet aggregation that might favor invasion. Risks of transfer or antimicrobial resistance (particularly vancomycin resistance) from LGG to other organisms appear to be low, based on the location of vancomycin resistance on the bacterial chromosome. There is no evidence of gastrointestinal toxicity to date.

2.1.7 Previous Human Experience with Lactobacillus GG

2.1.7.1 Controlled Trials

One recently published systemic review on use of probiotics for treating infectious diarrhea (Cochrane Collaboration [130]) noted the variable quality and variety of probiotics used in the published literature. Specifically, in the Cochrane review of 64 potentially relevant studies, only 23 were included in their analysis based on methodologic quality. There are similar concerns for trials in normal volunteers as well as adults and children with a wide range of conditions who have been treated with LGG. Since this section focuses on previous human experience, we have included all published trials, regardless of methodologic quality. We have included all studies in which the probiotic was specified with “GG” as either LGG, *Lactobacillus* GG*, Lactobacillus rhamnosus* GG, or identified as ATCC 53103.

Normal Volunteer Studies: Almost 4,000 healthy adults and children have participated in 34 clinical trials involving the administration of LGG. Tables 1-3 provide the details of these studies.

Table 1 shows the 19 clinical trials involving 1,267 healthy adults who consumed LGG. In 3 of the trials, the authors explicitly stated that the healthy adults did not experience adverse events. In 2 of the trials, the authors reported 5 subjects who had non-serious adverse events. No adverse events were reported in 13 trials. In our pilot study (Davidson et al, 2010 submitted) that is very similar to the proposed trial, the 42 healthy adults were asked open ended questions about adverse events and then asked about specific adverse events seen in probiotic trials as well as associated with administration of LAIV. One or more adverse events was reported at the 2, 4 or 8 week visit in 14/21 (67% of LGG subjects) and 17/21 (81% of placebo subjects). One subject in the placebo group was hospitalized for a sinus infection on study day 58. This event was considered serious but unrelated to the study protocol. Other non-serious adverse events occurring at any time after LAIV administration included: possibly LAIV related - rhinorrhea (9 in the LGG group, 9 in the placebo group), headache (6 in the LGG group, 6 in the placebo group), cough (2 in the LGG group, 6 in the placebo group), muscle aches (0 in the LGG group, 6 in the placebo group), sore throat (1 in the LGG group, 4 in the placebo group), weakness (0 in the LGG group, 3 in the placebo group), chills (0 in the LGG group, 2 in the placebo group); possibly probiotic related – gas (4 in the LGG group, 4 in the placebo group), nausea (6 in the LGG group, 2 in the placebo group), rumbling (2 in the LGG group, 3 in the placebo group), decreased appetite (0 in the LGG group, 5 in the placebo group), bloating (1 in the LGG group, 2 in the placebo group), diarrhea (1 in the LGG

group, 2 in the placebo group), abdominal pain (1 in the LGG group, 1 in the placebo group) and other symptoms (2 in the LGG group, 5 in the placebo group).

These studies show that LGG may colonize the GI tract for a short duration (determined by detecting LGG in the stool) after the subjects had stopped consuming LGG. Goldin [103] found LGG in the stool in 87% of the subjects 3 days after stopping LGG and 33% of the subjects 7 days after stopping LGG, but this study was published in 1992 when molecular methods for the precise identification of LGG versus other Lactobacilli were not available. Saxelin's study [131] had similar results through day 7 after stopping LGG. Alander [132] found LGG (confirmed by PCR) in biopsy samples from the colonic mucosa in 2 out of 7 subjects 28 days after discontinuing LGG. The fecal samples were negative at that time. These results were obtained despite clean out procedures used in preparation for colonoscopy and indicate that colonization can persist for at least one month after LGG has been stopped. Immunomodulatory effects of LGG in normal subjects are not clear [133].

TABLE 1: Studies of Lactobacillus GG ATCC 53103 in Healthy Adults

Author	Healthy Subject Age	Study Reason	Probiotic Organism	Probiotic Manufacturer	Probiotic Dose	Probiotic Duration	Control	Probiotic # subjects	Controls # subjects	Adverse Events
Siitonen, 1990 [134]	18-24 y	Assess gastrointestinal side effects while taking erythromycin	Lactobacillus GG	NS	Dose not clear - LGG fermented yogurt, 125 ml/day	7 days	Pasteurized regular yogurt with no live bacteria	8	8	None reported
Oksanen, 1990 [135]	10-80 y	Travelers, to prevent diarrhea	Lactobacillus GG	Valio, Helsinki, Finland	$2x10^8$ cfu/day	NS	Ethyl cellulose powder	402	418	Stated none occurred
Goldin, 1992 [103]	21-55 y	Determine colonization	LGG frozen concentrate, yogurt or whey drink	Yogurt - D Brown, Cornell, Frozen concentrate or whey NS	$4x10^8$/day frozen; $3.6x10^{11}$/day yogurt; $1.6x10^{11}$/day whey	4 weeks concentrate; 7 days yogurt; 35 days whey	N/A	76 (3 groups: 15 - frozen, 15 - yogurt, 46 – whey)	N/A	None reported
		Determine colonization while taking ampicillin			Dose not clear	10 days	N/A	37	N/A	None reported
Ling, 1994 [136]	20-41 y	Study mechanisms	Lactobacillus GG	NS	$3x10^{10}$ cfu/day	4 weeks	Pasteurized yogurt and fiber product	42 (2 groups: 21 - yogurt; 21 - yogurt + fiber)	22	None reported
Saxelin, 1995 [131]	20-55 y	Determine colonization	Lactobacillus GG ATCC 53103	NS	2 doses: low dose - $1.6x10^8$ cfu/day, high dose - $1.2x10^{10}$ cfu/day	7 days	N/A	20 (2 groups: 10 - low dose, 10 - high dose)	N/A	None reported

Author	Healthy Subject Age	Study Reason	Probiotic Organism	Probiotic Manufacturer	Probiotic Dose	Probiotic Duration	Control	Probiotic # subjects	Controls # subjects	Adverse Events
Benno, 1996 [137]	29-53 y	Determine colonization	Lactobacillus GG	Takanashi Milk Products o, Ltd, Yokohama, Japan	2 dosages: $1.4x10^{10}$ cfu/day; $2.8x10^{10}$ cfu/day	$1.4x10^{10}$ cfu/day x 4 weeks; $2.8x10^{10}$ cfu/day x 1 day	N/A	13 (2 groups: 8 - $1.4x10^{8}$ cfu/day, 5 - $2.8x10^{8}$ cfu/day)	N/A	None reported
Hilton, 1997 [138]	>18 y	Travelers, to prevent diarrhea	Lactobacillus GG	NS	$2x10^{9}$ cfu/day	2 days prior to departure and throughout trip	Ethyl cellulose powder	200	200	2 due to LGG - abdominal cramping
Pelto, 1998 [139]	20-50 y	Study mechanisms	LGG ATCC 53103	Valio, Helsinki, Finland	$2.6x10^{8}$ cfu/day	1 week	Milk	17 (cross-over)	17 (cross-over)	None reported
Alander, 1999 [132]	27-78 y	Determine colonization (during routine colonoscopy)	Lactobacillus rhamnosus GG ATCC 53103	Valio, Helsinki, Finland	$6x10^{10}$ cfu/day	12 days	N/A	21 (3 groups: 6 - colonoscopy as LGG stopped, 8 - colonoscopy 1 week after LGG, 7 - colonoscopy 2 weeks after LGG)	N/A	None reported
Fang, 2000 [91]	20-50 y	Study immune response to Salmonella typhi Ty21a oral vaccine	Lactobacillus GG ATCC 53103	Valio, Helsinki, Finland	$4.0x10^{10}$ cfu/day	7 days	Ethyl cellulose	10	9	None reported
Gotteland, 2001 [140]	18-38 y	Assess gastrointestinal effects after taking 2 doses of indomethacin	Combination - Lactobacillus GG + L helveticus + L acidophilus	Soprole, Santiago, Chile	LGG $2.4x10^{9}$ cfu/day + L. helveticus and L. acidophilus $2.4x10^{9}$ cfu/day each	5 days	Heat-killed lactic acid bacteria	18	18	None reported

Author	Healthy Subject Age	Study Reason	Probiotic Organism	Probiotic Manufacturer	Probiotic Dose	Probiotic Duration	Control	Probiotic # subjects	Controls # subjects	Adverse Events
Ahola, 2002 [141]	18-35 y	Study mechanisms	Combination - Lactobacillus rhamnosus GG ATCC 53103 + Lactobacillus rhamnosus LC 705	Valio, Helsinki, Finland	LGG $1.4x10^9$ cfu/day + L rhamnosus LC 705 $9x10^8$ cfu/day	3 weeks	Edam cheese with 16% fat but without bacteria	41	42	None reported
Gluck, 2003 [142]	41±8 y, 39±9 y	Assess effects on nasal colonization of bacteria	Combination - Lactobacillus GG ATCC 53103 + Bifidobacterium sp B420 + L acidophilus 145 + S. thermophilus	Emmi Schweiz AG, Lucerne, Switzerland	LGG $4.6x10^{11}$ cfu/day + ST $1.8x10^{12}$ cfu/day + LA $2x10^{11}$ cfu/day + Bb $5.5x10^{11}$ cfu/day	3 weeks	Standard yogurt	108	101	None reported
Schultz, 2003 [133]	21-43 y	Study mechanisms	Lactobacillus rhamnosus GG	ConAgra Functional foods, Omaha, Nebraska	$2x10^9$ cfu/day	35 days	N/A	10	N/A	3 due to LGG - mild abdominal bloating and meteorism
Cohen, 2007 [143]	25-45 y	Assess effects during administration of isoflavone	Lactobacillus GG	ConAgra Functional foods, Omaha, Nebraska	$4x10^{12}$ cfu/day	4 weeks per group	Soy protein mixture	32 (cross-over, 2 groups: soy protein + LGG, LGG alone)	32 (cross-over group)	Stated none occurred
Kekkonen, 2007 [144] Moreira, 2007 [145]	Mean age 39-40 y	Study mechanisms	Lactobacillus rhamnosus GG ATCC 53103	Valio, Helsinki, Finland	$4x10^{10}$ cfu/day by bottle or $1x10^{10}$ cfu/day by capsule	3 months	2 options: milk-based fruit drink or capsules	71	70	Stated none occurred
Kekkonen, 2008 [146, 147]	23-58 y	Study mechanisms	LGG ATCC 53103	NS	$1.6x10^{10}$ cfu/day	3 weeks	3 groups: Bifidobacterium (Bb12), Propionibacterium freundenreichii ssp shermanii JS (PFS), placebo	13	49 (3 groups: 16 - Bb12, 17 - PFS, 16 - placebo)	None reported

Author	Healthy Subject Age	Study Reason	Probiotic Organism	Probiotic Manufacturer	Probiotic Dose	Probiotic Duration	Control	Probiotic # subjects	Controls # subjects	Adverse Events
Gluck, 2003 [142]	41±8 y, 39±9 y	Assess effects on nasal colonization of bacteria	Combination - Lactobacillus GG ATCC 53103, Bifidobacterium sp B420, L acidophilus 145, S. thermophilus	Emmi Schweiz AG, Lucerne, Switzerland	LGG $4.6x10^{11}$ cfu/day + ST $1.8x10^{12}$ cfu/day + LA $2x10^{11}$ cfu/day + Bb $5.5x10^{11}$ cfu/day	3 weeks	standard yogurt	108	101	None reported
Davidson, 2010 (submitted)	18-49 y	Assess LGG as immune adjuvant to LAIV	Lactobacillus GG ATCC 53103	Chr Hansen, Denmark	$2 x 10^{10}$ cfu 2x/day	4 weeks	Microcrystalline cellulose in gelatin capsules	21	21	14 in LGG group, 17 in placebo – details in text above

Legend NS - not specified d – days, y – year, mo- months CFU - colony forming units

Table 2 shows the 9 clinical trials involving 664 healthy children who consumed LGG. In 3 of the trials, the authors explicitly stated that the healthy children did not experience adverse events. In 3 of the trials, the authors reported non-serious adverse events, but with no difference between the LGG and placebo groups. One of these studies involved concurrent administration of rotavirus vaccine, which may have resulted in the presence of fever [90]. No adverse events were reported in the remaining 3 trials.

Sepp [148] demonstrated that LGG could be recovered from stool in neonates given LGG for the first 2 weeks of life, but this study published in 1993 did not use molecular methods to identify the precise *Lactobacillus* spp. In 1995 Sheen [149] reported recovering 8/11 LGG from any stool and 6/11 LGG from multiple stools during a 10-day administration of LGG to infants aged 6-24 months, again using non molecular methods. Agarwal [150] reported presence of LGG on the last day of LGG administration (this is not necessarily colonization) in 5/24 (21%) of lower birth weight infants and 11/23 (47%) of higher birth weight infants. Non-molecular methods were used to detect LGG. Petschow [151] reported detection of LGG in feces (using non molecular methods) during LGG administration in infants aged 0-3 months receiving low dose LGG - 8/12 (67%); medium dose LGG - 11/13 (85%); and high dose - 10/12 (83%). The details of the daily dose are shown in Table 2. Twenty-eight days after discontinuing LGG, colonization was reported as 7/12 (58%); medium dose LGG - 5/13 (45%); and high dose – 2/11 (18%). Taken together, these results suggest that colonization with LGG, after LGG administration has stopped, can occur, but precise duration of colonization confirmed by molecular methods is not clear.

TABLE 2: Studies of Lactobacillus GG ATCC 53103 in Healthy Children

Author	Healthy Child Age	Study Reason	Probiotic Organism	Probiotic Manufacturer	Probiotic Dose	Probiotic Duration	Control	Probiotic # subjects	Controls # subjects	Adverse Events

Author	Healthy Child Age	Study Reason	Probiotic Organism	Probiotic Manufacturer	Probiotic Dose	Probiotic Duration	Control	Probiotic # subjects	Controls # subjects	Adverse Events
Sepp, 1993 [148]	<1 mo	Determine colonization	Lactobacillus GG	NS	Dose not clear, 10^{10}-10^{11} cfu/g	First 2 weeks of life	None	15	10	None reported
Isolauri, 1995 [90]	60-150 d	Study immune response to rotavirus vaccine	LGG ATCC 53103	Valio, Helsinki, Finland	$1x10^{11}$ cfu/day	5 days	Microcrystalline cellulose	30	30	Temperature > 38 in 17% (LGG) vs 14% (control), Vomiting - 2 in LGG group
Sheen, 1995 [149]	6-24 mo	Determine colonization	LGG	NS	$5x10^{8}$ cfu/day	10 days	Heat-killed LGG	11	13	None reported
Hatakka, 2001 [152]	1.3-6.8 y	Daycare attendees to prevent infections	Lactobacillus rhamnosus GG	Valio, Helsinki, Finland	>1-$2x10^{8}$ cfu/day	7 months	Milk without probiotic	282	289	Stated none occurred
Agarwal, 2003 [150]	<1 mo	Determine colonization	Lactobacillus rhamnosus GG	Valio, Ltd. USA	$2x10^{9}$ cfu/day	2 groups: <1500g: LGG initiated on day 2-3 of life x 21 days; 1500-1999g, LGG initiated on day 1-3 of life x 8 days	Nonsupplemented breast milk feed	47 (2 groups: 24 - <1.5 kg, 23 - 1.5-1.99 kg)	24 (2 groups: 15 - <1.5 kg, 9 - 1.5-1.99 kg)	Stated none occurred
Petschow, 2005 [151]	0-3 mo	Determine colonization	Lactobacillus GG	NS	3 dosages: 10^{8} cfu/day, 10^{9} cfu/day, 10^{10} cfu/day	14 days	Nutramigen powder	44 (3 groups: 15 - low dose, 14 - medium dose, 15 - high dose)	15	Thrush, nasal congestion, diaper rash, and upper respiratory infection similar in all groups
Rautava, 2006 [153]	2-65 d	Study general health in bottle-fed children	Combination - Lactobacillus GG ATCC 53103 + Bifidobacterium brevis 12	Valio, Helsinki, Finland (LGG), Chr Hansen, Denmark (Bb12)	$1x10^{10}$ cfu/day, both probiotics	To age 1 year	Microcrystalline cellulose	38	43	3 due to GI complaints
Vendt, 2006 [154]	<2 mo	Assess children's growth	Lactobacillus rhamnosus GG ATCC 53103	Valio Ltd, Helsinki, Finland	Dose not clear - 10^{7} cfu/g	To 6 months of age	Tutteli Infant Formula	60	60	Stated none occurred

Author	Healthy Child Age	Study Reason	Probiotic Organism	Probiotic Manufacturer	Probiotic Dose	Probiotic Duration	Control	Probiotic # subjects	Controls # subjects	Adverse Events
Smerud, 2008 [155]	12-36 mo	Daycare attendees to prevent infections	Combination – LGG + Bifidobacterium b12 + L acidophilus LA-5	TINE BA, Norway	LGG $1.5x10^{10}$ cfu/day + Bb12 $1.5x10^{10}$ cfu/day + LA-5 $1.5x10^{9}$ cfu/day	7 months winter	Heated fermented milk drink	117	123	None reported

Legend NS - not specified d – days, y – year, mo-months CFU - colony forming units

Table 3 shows the 7 clinical trials involving 1,111 healthy pregnant women who consumed LGG and the 991 healthy children who continued to consume LGG from birth. In 4 of the trials, the authors explicitly state that the healthy children did not experience adverse events. In 1 of the trials, the authors report no difference between the LGG and placebo groups, including rates of hospitalization (serious adverse event). The authors of this study state that hospitalizations through the first 24 months of life (18 months after study therapy was discontinued) were not related to LGG in combination with other probiotics or placebo [156]. No adverse events were reported in the remaining 2 trials.

Schultz [157] treated pregnant women with LGG (not their infants) and noted that at 1 and 6 months, all 4 vaginally-delivered children and half of the children delivered by c-section were colonized with LGG. Two children had LGG detected 24 months after delivery. LGG was confirmed by PCR. The mothers discontinued LGG at delivery. These data suggest that long-term colonization can occur. Children in the Kalliomaki study who were exposed to LGG during gestation and for 6 months after birth have now been followed for 7 years without adverse events [158].

TABLE 3: Studies of Lactobacillus GG ATCC 53103 in Healthy Pregnant Women and Newborns

Author	Healthy Pregnant Women and Newborn Age	Study Reason	Probiotic Organism	Probiotic Manufacturer	Probiotic Dose	Probiotic Duration	Control	Probiotic # subjects	Controls # subjects	Adverse Events
Kalliomaki, 2001 [159]; Rautava, 2002 [160]; Kalliomaki 2003 [161]; Laitinen 2005 [162]; Kalliomaki 2007[158]	Pregnant women age NS, infants 0-6 mo	Prevent atopic eczema in infants	LGG	Valio, Helsinki, Finland	$2x10^{10}$ cfu/day	2-4 weeks before delivery, 6 months afterwards, breastfeeding mothers took capsules, non breastfed infants took LGG	Microcrystalline cellulose	77	82	Stated none occurred – Please note study reports now extend through 7 years of infant follow-up [158]
Schultz, 2004 [157]	Pregnant women age NS	Determine colonization in infants	Lactobacillus rhamnosus GG ATCC 53103	ConAgra Functional foods, Omaha, Nebraska	$2x10^{9}$ cfu/day	Self administered in late pregnancy	None	6	3	Stated none occurred

Author	Healthy Pregnant Women and Newborn Age	Study Reason	Probiotic Organism	Probiotic Manufacturer	Probiotic Dose	Probiotic Duration	Control	Probiotic # subjects	Controls # subjects	Adverse Events
Gueimonde, 2006 [163]	Pregnant women age NS, infants (via breast milk 0-3 weeks)	Determine colonization in infants	Lactobacillus GG	NS	Dose NS	2-4 weeks before delivery, 3 weeks afterwards via breast milk	NS	29	24	None reported
Rinne, 2006 [164]	Pregnant women age NS, infants 0-6 mo	Determine colonization in infants	Lactobacillus rhamnosus GG ATCC 53103	Not described	$1x10^{10}$ cfu/day	2-4 weeks before delivery and 6 months after birth (mothers could consume themselves or give to infants)	Microcrystalline cellulose	64	68	Stated none occurred
Kaplas, 2007 [165]	Pregnant women 25-36 y	Healthy pregnant women to evaluate placenta phospholipid fatty acids	Combination - Lactobacillus GG ATCC 53103 + Bifidobacterium brevis 12	Valio, Helsinki, Finland (LGG), Chr Hansen, Denmark (Bb12)	10^9 cfu/day, both probiotics	2nd trimester to birth	2 groups microcrystalline cellulose, dietary counseling	10	20 (2 groups: 12 - diet, 8 – placebo)	None reported

Author	Healthy Pregnant Women and Newborn Age	Study Reason	Probiotic Organism	Probiotic Manufacturer	Probiotic Dose	Probiotic Duration	Control	Probiotic # subjects	Controls # subjects	Adverse Events
Kukkonen, 2007 [166]; Kukkonen 2008 [156]	Pregnant women age NS, infants 0-6 mo	Healthy pregnant women and their newborns to prevent allergic diseases	Combination - Lactobacillus rhamnosus GG ATCC 53103 + Lactobacillus rhamnosus LC705 + Bifidobacterium Bb99 + Propionibacterium freundenreichii spp shermanii JS	Valio, Helsinki, Finland	Mothers: LGG $1x10^{10}$ cfu/day + L rhamnosus LC705 $1x10^{10}$ cfu/day + Bifidobacterium breve Bb99 $4x10^{8}$ cfu/day + Propionibacterium freudenreichii ssp. shermanii JS $4x10^{9}$ cfu/day; Infants: half adult dose	Mothers: 2-4 weeks before delivery; infants: 6 months after birth	Microcrystalline cellulose for mothers and infants; for infants, sugar syrup	610 mothers, 506 infants	613 mothers, 512 infants	35 (Combination) 37 (control) abdominal discomfort; vomiting 7 (combination) and 12 (control); 13 (combination), 9 control) excessive crying; - Please note this study now extends thru 2 years – hospitalizations in 2 groups similar and unrelated to intervention
Kopp 2008 [167, 168]	Pregnant women age 95% percentiles 26.5-40.3 y, infants 0-6 months	Healthy pregnant women and their newborns to prevent atopic eczema	Lactobacillus GG ATCC 53103	Infectopharm, Heppenheim, Germany	$1x10^{10}$ cfu/day	4-6 weeks before delivery; breastfeeding mothers for 3 months then children for 3 months; not breastfeeding - children for 6 months	Microcrystalline cellulose	54	51	Stated none occurred

Legend NS - not specified d – days, y-year, mo-months CFU - colony forming units

2.1.7.2 Other Published Material Relevant to Safety

Table 4 lists the reported cases of invasive disease due to consumption of LGG.

Table 4: Published Case Reports of Invasive Infection due to LGG or ATCC Strain 53103

	Patient		Risk Factors				Probiotic Use		Invasive Infection		
Reference	Age	Gender	Underlying Condition	Immunocompromise	ICU	Central Line	Consumed	Indication	Positive Cultures	Molecular Methods	Treatment and Outcome
Rautio et al., 1999 [169]	74 y	F	Diabetes mellitus	Diabetes	NS	NS	L. rhamnosus GG 0.5 L diary products/day (brand NS)	Self administered for abdominal discomfort	Liver abscess	PCR and PFGE	Pen G, Pip-Tazo, Cipro, Clina – Patient survived

	Patient		Risk Factors				Probiotic Use		Invasive Infection		
Reference	Age	Gender	Underlying Condition	Immunocompromise	ICU	Central Line	Consumed	Indication	Positive Cultures	Molecular Methods	Treatment and Outcome
MacKay et al., 1999 [170]	67 y	M	Mitral valve regurgitation dental extraction (amoxicillin prophylaxis)	NS	NS	NS	Lactobacillus rhamnosus, $3x10^9$ cfu/day, L acidophilus (dose NS), S faecalis (dose NS) (brand NS)	Self administered	Blood	API 50 CH, pyrolysis mass spectrometry	Amp, Gent – Patient survived
Kunz et al., 2004 [171]	3 mo	M	Short gut syndrome	Born at 36 weeks	NS	NS	LGG (Culturelle) 1 capsule/day (dose NS) ConAgra, Omaha, Nebraska	Not clear, possibly to treat cholestasis	Blood	No confirmatory testing	Amp – Outcome not stated
	<1 mo	M	Short gut syndrome, gastrochisis	Born at 34 weeks	NS	NS	LGG 1 capsule/day (dose and brand NS)	Prevent small bowel bacterial overgrowth	Blood	PFGE	Ceftr, amp – outcome not stated
Land et al., 2005 [172]	4 mo	M	Cardiac surgery, multiple post-operative complications	NS	NS	Y	LGG (Culturelle) $10x10^9$ cfu/day (ConAgra Foods)	Treat diarrhea	Blood, catheter infected	PCR	Catheter removed, Pen G, Gent – Patient survived
	6 y	F	Cerebral palsy, jejunostomy feeding, microcephaly, seizure disorder	NS	NS	Y	LGG (Culturelle) $10x10^9$ cfu/day (ConAgra Foods)	Treat diarrhea	Blood, catheter infected	PCR	Amp – Patient survived
De Groote et al., 2005 [173]	11 mo	M	Necrotizing Enterocolitis, Short gut syndrome, parenteral nutrition	Previously Premature (26 weeks)	NS	Y	L. rhamnosus GG, 1/8 capsule 2x/day (dose NS, brand NS)	Treat rotavirus diarrhea	Blood obtained via catheter	PFGE	Catheter removed, vanco, ceftaz

Legend

NS – not stated	y – year, mo-months	Y-yes
PCR - polymerase chain reaction	PFGE - pulsed field gel electrophoresis	CFU - colony forming units
Amp – Ampicillin	Gent – Gentamicin	Ceftr - Ceftriaxone
Vanco – Vancomycin	Pen G - Penicillin G	Ceftaz - Ceftazidine
Pip-Tazo - Pipericillin-Tazobactam	Cipro - Ciprofloxacin	Clinda - Clindamycin

Acceptable molecular methods used to determine whether the invasive isolate matched the consumed probiotic include polymerase chain reaction (PCR) and pulsed field gel electrophoresis (PFGE). Use of the API 50 CHI identification system is not adequate [174]. Although, the second

case report used only API 50 CHI for comparison of the invasive and probiotic isolate, we have included it in the Table for completeness of reported cases.

Epidemiologic Studies – LGG is used in more than 30 countries and an estimated 3 million kilograms of LGG-containing products were safely consumed by a minimum of 40,000 persons in Finland alone in 1992 [175]. Salminen et al recently evaluated the possible effects of increased use of LGG in Finland since 1990 by studying Lactobacillus bacteremia at the Helsinki University. Lactobacilli were isolated in 0.02% of all blood cultures with positive results in Helsinki University Central Hospital and in Finland as a whole. No trends were seen that suggested an increase in Lactobacillus bacteremia. The average incidence was 0.3 cases/100,000 inhabitants/year in 1995-2000 in Finland. Identification to the species level was done for 66 cases of Lactobacillus bacteremia, and 48 isolates were confirmed to be *Lactobacillus* strains. Twenty-six of these strains were *L rhamnosus*, and 11 isolates were identical to *L rhamnosus* GG. The results indicate that increased probiotic use of *L rhamnosus* GG has not led to an increase in Lactobacillus bacteremia [175]. The same authors recently reviewed the 89 cases with Lactobacillus bacteremia reported between 1990 and 2000, to study the risk factors and outcomes in these patients [176]. Of the 89 cases, the blood isolate was not confirmed to be *Lactobacillus* in 42, was a non-rhamnosus *Lactobacillus* in 22, was *L rhamnosus* but not GG in 14, and was *L rhamnosus* GG in 11. Patient charts were reviewed for predictors of mortality. LGG use by these patients was not studied. Mortality after *L rhamnosus* bacteremia (LGG and non LGG combined) was associated with severe or fatal co-morbidities). In a recent Finnish study, three of these “LGG-like” blood isolates were examined closely for phenotypic characteristics, and while they may not be distinguishable from LGG by pulsed-field gel electrophoresis, they were found to differ from LGG in other characteristics such as adhesion properties, resistance to serum-mediated killing, and induction of respirator burst [177].

Synthesis – The methodologic quality of some of the literature on LGG limits some of the conclusions that can be made about safety. Overall, almost 4,000 healthy adults, children, and pregnant women have received LGG. Since use of LGG in patients in intensive care units, particularly those with central venous catheters, or in patients who are on immunosuppressive therapy, have short gut syndrome, or are at risk for endocarditis has rarely associated with invasive disease due to the probiotic strain (7 case reports), these risk factors are exclusion criteria in our proposed study population.

2.1.8 Effect of Probiotics on the Immune System

2.1.8.1 Effects of Probiotics on Systemic and Mucosal Humoral Immune System

Probiotics are widely available in Europe and the United States and although they are advertised as promoting immunity or boosting the immune system, few human studies have evaluated their immunomodulatory properties. In vitro and animal data suggest that probiotics increase levels of intestinal IgA and upregulate cytokine production. Human studies provide a strong rationale for the study of probiotics as a vaccine immune adjuvant. In a study of healthy Japanese infants,

administration of bifidobacteria containing formula resulted in an elevation of total IgA and anti-poliovirus IgA levels in the feces [178]. Similarly, 30 healthy subjects were randomized to LGG, *Lactococcus lactis* or placebo for 7 days, prior to receiving oral *Salmonella typhi* vaccine (10 per group). Although there was insufficient power to detect differences between groups, there was a trend towards higher specific IgA anti-Salmonella antibodies in the LGG treated group [91]. Other investigators randomized healthy subjects to receive either fermented milk with L acidophilus and bifidobacteria vs. control for 3 weeks during which oral *Salmonella typhi* vaccine was administered. The experimental group had significantly higher total serum IgA and specific serum IgA to *S typhi* [179]. Finally, responses to administration of DxRRV rhesus-human reassortant live oral rotavirus vaccine were compared in infants randomized to LGG vs placebo. Infants receiving LGG had higher rates of IgA seroconversion and rises in rotavirus specific IgM secreting cells vs. infants receiving placebo [90].

Further support for the use of probiotics in combination with mucosally administered vaccine comes from natural studies of rotavirus infection. Several studies have evaluated the effect of LGG on rotavirus infection. In one study, 44 previously healthy children with acute rotavirus infection were randomized to receive either LGG or pasteurized yogurt without LGG [180]. The LGG treated group had a decrease in duration of symptoms and an increased nonspecific response in IgG, IgM, and IgA compared with placebo treated children. In the convalescent phase, there was a significant increase in the IgA antigen specific antibody secreting cells to rotavirus in the LGG group compared to the placebo treated group. In a separate study, LGG vs. *Lactobacillus casei* subsp. rhamnosus (Lactophilus) or a combination of *Streptococcus thermophilus* and *Lactobacillus delbruckii* subsp. bulgaricus (Yalacta) was administered twice daily for 5 days to children with rotavirus infection. Those receiving LGG had higher serum IgA and specific antibody secreting cells to rotavirus during the convalescent phase of rotavirus infection [181]. Kaila et al had a similar result when children with acute rotavirus infection were treated with viable versus inactivated LGG – those who received viable LGG had a significant increase in serum rotavirus specific IgA and rotavirus specific IgA secreting cells [182].

The majority of research in probiotics has previously focused on pathogens that enter the human host via the enteral route. However, the effects of secretory IgA are not limited to the gastrointestinal tract. Secretory IgA antibodies may be found throughout the mucosal immune system, including the respiratory tract, salivary glands, and lacrimal glands. In addition, the antigen presenting cells of the Peyers patches and the local gut immune system may interact with lymphocytes that induce both an innate and humoral immune responses at distal sites. Animal studies have started to focus on the effect of probiotics on distal mucosal sites. In a mouse model looking at the affects of Lactobacillus on infection with *Streptococcus pneumoniae*, mice were challenged with *Lactobacillus fermentum, S pneumoniae*, or *L fermentum* and then *S pneumoniae* (experimental group) [183]. Anti *S pneumoniae* antibodies were increased in the *Lactobacillus* treated mice compared with controls, and *Lactobacillus* treated mice had an increased number of macrophages in the lung and lymphocytes in the trachea. Another study by Alvarez et al. looked at mice who were challenged with aerosolized *Pseudomonas aeruginosa* [184]. Mice fed a diet of

lactobacilli had a significant increase in IgA and IgM levels in bronchoalveolar lavage samples after infection with *P aeruginosa*.

While these animal studies show the potential for immune modulation in the respiratory tract after administration of lactobacilli during bacterial infections, Hori et al. investigated the effect of oral *Lactobacillus casei* strain Shirota on influenza infection of the upper respiratory tract in mice [185, 186]. Those on an *L casei* diet had a lower titer of influenza in nasal washings and an increase in NK cell function, interferon gamma and TNF-alpha. In a similar study using mouse model of influenza infection of the upper respiratory tract, the titer of virus in the nasal washings of infant mice receiving *L. casei* Shirota (*L. casei* Shirota group) was significantly lower than that in infant mice receiving saline [187]. Taken as a whole, there is promising indirect evidence in support of the potential to boost immune response to at least LAIV based on administration of other oral live vaccines in humans as well as direct evidence in support of improved immune response to an influenza virus challenge in mice treated with LGG.

2.1.8.2 Effects of Probiotics on the Innate Immune System

Both the innate and adaptive immune systems are modulated by probiotics. The initial interaction of orally delivered probiotics with the mucosal immune system occurs by interaction with epithelial cells in the lining of the gastrointestinal tract. These epithelial cells function as immunoregulatory cells. In vitro studies have shown that lactobacillus increases transepithelial resistance and may prevent decreased transepithelial resistance when administered with other pathogenic bacteria [188, 189]. The next step is recognition of lactobacillus molecular patterns by antigen presenting cells (macrophages and dendritic cells). Lactobacilli have been shown to upregulate cytokine expression in murine dendritic cells and human peripheral blood mononuclear cells in *in vitro* studies [85, 88, 190-192]. Lactobacillus has been shown *in vitro* to induce production and expression of Th-1 type cytokines TNFα, IL-2, IL-1β, IL-6 [193] and IL-18 in peripheral blood mononuclear cells [85, 190, 192, 194]. Lactobacillus has also been found to activate transcription factor NF-κB and Toll-like receptors (TLR 2 and 9) [86, 191, 195]. The upregulation of Th-1 type cytokines by Lactobacillus may play a role in the CTL response to influenza.

Probiotics have also been found to enhance the innate immune response in the elderly. In a trial of elderly and middle aged adults, patients who received *Lactobacillus rhamnosus* HN001 had increased polymorphonuclear (PMN) phagocytic activity and natural killer (NK) cell tumorcidal activity [196]. In a study of 30 healthy elderly subjects, *Bifidobacterium lactis* HN019 was found to increase the proportions of total, helper and activated T lymphocytes and NK cells [21, 197]. Phagocytic activity of mononuclear and (PMN) phagocytes and tumorcidal activity of NK cells was also increased. In a subsequent double blind placebo controlled study, elderly adults who received *Bifidobacterium lactis* HN019, the probiotics enhanced PMN and NK cell activity when compared with placebo [198]. In a similar study, *Bifidobacterium* was found to increase interferon alpha production in elderly patients [199].

2.1.8.3 Effects of Prebiotics and Probiotics on the Immune Response to Influenza Vaccination

Probiotics may enhance innate and adaptive immunity and augment the immune response to mucosally delivered vaccines (Fang 2000). Mice fed *Lactobacillus* prior to an influenza virus challenge had higher levels of influenza specific IgG and greater protection against illness (Yasui 2004). Few studies on the use of probiotics as an immune adjuvant to influenza vaccination have been published to date. In one study in which a prebiotic containing nutritional supplement was administered to elderly patients (age≥70) receiving the trivalent influenza vaccine, there was no effect of the prebiotic mixture on immunologic response. However, in a placebo controlled trial using a nutritional supplement containing *Lactobacillus paracasei,* elderly patients receiving the influenza and pneumococcal vaccines had an increase in the innate immune response and a decreased number of infections when compared with placebo [200]. Additionally, the results of both a pilot and confirmatory placebo-controlled randomized trial using a yogurt drink containing *Lactobacillus casei* demonstrated that elderly patients who took the probiotic during the influenza season had increased relevant specific antibody response to the vaccine when compared with placebo [201]. Probiotic *Lactobacillus fermentum (*CECT5716 [202] also was shown to improve immunogenicity of TIV in a human trial. However, there are no human studies of probiotics on immunogenicity of mucosally delivered LAIV.

During the 2007-2008 influenza season, our group conducted a study of LGG vs. placebo as an immune adjuvant to LAIV in 42 normal healthy subjects aged 18 to 49. Approximately 52% of the LGG group and 38% of the placebo group had previously receive TIV (P=0.54). Only 3 of the 42 (7%) were lost to follow up, all immediately after the baseline visit. Overall, 15% seroconverted for the H1N1 strain, 45% for the H3N2 strain and 41% for the B strain. In the intent to treat analysis, there was no difference in seroconversion rates between treatment and placebo groups for the H1N1 and B strains, but in the per protocol analysis there was a trend to increased seroprotection in the LGG group vs. placebo for the more generally immunogenic A/Wisconsin/67/2005 (H3N2) vaccine strain on day 14 (79% vs. 55%, p=0.11), day 28 (84% vs. 55%, p=0.08) and day 56 (84% vs. 60%, p=0.16) (Davidson et al, 2010, submitted). Although there were significantly more reports of myalgias and decreased appetite in the LGG group, adverse events were similar to those observed in other LAIV studies [203]. The proposed study uses the same regimen as was used in this proof of concept study.

2.1.9 Effects of Probiotics on the Microbiota and Host Immune Response

Evaluation of the bacteria that colonize the gastrointestional tract and nasopharynx has traditionally depended on culturing the organisms in the microbiology laboratory. These standard culture methods, even when optimized, can miss up to 80% of organisms that can be detected by newer culture independent methods[204]. High-throughput DNA sequencing provides new opportunities for understanding the microbial ecology of the gut and upper respiratory tract. These methods use the bacterial 16S rRNA to identify bacterial species. Since portions of the 16S rRNA are well conserved across both prokaryotes and archaea, PCR primers that span conserved regions can be

used to amplify the sequences from all the species in a sample. Highly variable intervening portions of the genome that are amplified with the conserved regions exhibit sufficient diversity to allow the identification of the separate species of bacteria that are present. Recent technological advances in sequencing can generate hundreds of thousands of sequences from a single sample (deep sequencing). Although originally applied to the analysis of environmental samples, this technology is now being applied to the study of complex human microbial communities[205].

To date, most studies describing the bacterial diversity and richness of the human microbiome have focused on the gastrointestinal tract[206-208]. The predominant bacterial species of the human colon are the Firmicutes and Bacteroides. Throughout the gastrointestinal tract, from mouth to colon, there are different ecological niches that may be exploited by particular bacteria[206]. Changes in the gut microbiota may be related to diet, age and underlying disease[209-211]. Studies of the gastrointestinal tract in healthy newborns in the first year of life have shown a marked shift in gut microflora dependent on diet, environmental exposures and antibiotic use[212]. Studies of probiotics in newborns to prevent food allergies and in young children to prevent diarrhea have also suggested that changes in gut microflora may have profound effects on the host immune system[135, 160, 161, 213-216]. Recent seminal studies by Gordon et al have also suggested that these changes are not just due to interaction between the probiotics and the immune system, but more likely due to interactions with and changes in the microbial ecology of the gut [207, 217, 218].

The microbiota of the upper respiratory tract, particularly of the nasopharynx has not been studied as extensively as the microbiota of the colon. A recent study using 16S rRNA gene amplification techniques recently demonstrated the presence of over 700 species in the oral cavity of healthy subjects, over 50% of which were not identified by culture-based methods [219]. Using 16S rRNA gene sequence and reverse capture checkerboard hybridization, the predominant bacterial species in the oral cavity of healthy subjects and those with dental caries differed significantly [220].

However, there are no data on the microbiome of the nasopharynx in elderly subjects, nor on the effect of probiotics on the nasopharyngeal microbiome. Given the interest and importance of understanding the human microbiota and the ways that probiotics may alter the composition and function of the microbial communities inhabiting the GI and respiratory mucosal surfaces, we propose to study the effect of influenza immunization with and without probiotics.

Finally, at the request of NIH, we are collaborating with Dr Solano-Aguilar in the Diet, Genomics and Immunology Laboratory at the USDA, Beltsville, Maryland. The purpose of this collaboration is to evaluate the effects of LGG vs. placebo on the intestinal microbiota (specifically *Bifidobacterium* and *Lactobacillus spp*. in stool samples) and host immune response (specifically inflammatory/immune response genes).

2.1.10 Summary of the Background and Significance

- Influenza infections remain a major public health threat every year and when pandemics occur, the impact is even more substantial.
- There is room for improvement in the immunogenicity and efficacy of current influenza vaccines especially in the elderly and patients with chronic medical conditions
- Concomitant administration of LGG with oral live vaccines (polio, rotavirus and *S typhi*) in humans has resulted in enhanced humoral immunity against vaccine strains.
- Administration of LGG prior to influenza virus challenge results in improved immune response and clearing of the infection in the mouse model and preliminary data in younger healthy adults is encouraging.
- The novel technologies (454 pyrosequencing) that now permit detection of the human microbiota using non-culture based methods provide a timely and highly relevant way to evaluate whether probiotics such as LGG can safely modify the microflora of the gut and respiratory mucosa.
- Use of DNA and RNA PAXgene kits to study immune response gene polymorphisms and gene expression provide an opportunity to start to evaluate possible systemic immune effects of probiotics such as LGG.

This research will investigate whether LGG is an effective immune adjuvant to the influenza vaccine in elderly subjects as a first step towards the goal of boosting immune response in the elderly. LGG has the potential to be an easily accessible, cost effective influenza vaccine adjuvant that can safely be used in the elderly. It will be conducted as a Phase I investigation in three stages. The first stage will be an open label controlled trial to assess the safety of LGG in elderly subjects. The open label study will be completed and approval from the FDA will be obtained prior to proceeding to the second stage. The second stage will be a double blind placebo controlled randomized trial of LGG vs. placebo in elderly subjects receiving TIV during the influenza season, to assess the safety of LGG during administration of TIV. This second stage study will be completed and approval from the FDA will be obtained prior to proceeding to the third stage. The third stage will be a double blind placebo controlled randomized trial of LGG vs. placebo in elderly subjects receiving LAIV after the typical influenza season, to assess the safety of LGG during administration of LAIV. A future Phase II study may randomize elderly subjects to TIV or LAIV and LGG or placebo in a factorial design, depending on the results of the 3 stages of the Phase I study. This study protocol is for the first stage of the Phase I study only - an open label controlled trial to assess the safety of LGG in elderly subjects.

3 OBJECTIVES AND OUTCOMES

3.1 Study Objectives

Primary Objective - Assess the safety and tolerability of 2 x 10^{10} CFU LGG administered orally to elderly subjects for 28 days.

Secondary Objective - Evaluate the richness and microbial diversity in nasopharyngeal and stool specimens using pyrosequencing.

Optional Sub-Study Objectives

Compare cytokine production in response to bacterial stimulation by following the kinetics of mRNA expression of pro and anti-inflammatory genes and different signaling pathways in relation to changes in stool *Bifidobacterium* and *Lactobacillus spp*.

3.2 Outcome Measures

3.2.1 Primary Outcome Measure

Occurrence of adverse events defined as a new Grade II-IV toxicity (FDA's Guidance for Industry: Toxicity Grading Scale for Healthy Adult and Adolescent Subjects Enrolled in Preventative Vaccine Clinical Trials, September 2007), that are possibly or probably related to administration of LGG. Adverse events will be detected during study visits with standardized questionnaires, medical history, vital signs, physical examinations, laboratory tests and review of subject diaries as well as between study visits on telephone calls based on responses to adverse event questionnaires.

3.2.2 Secondary Outcome Measures

Richness and bacterial diversity of the nasopharngeal and gut microbiota and presence of LGG in stool specimens by routine culture

3.2.3 Optional Sub-study Outcome Measures

mRNA expression TNF-a, IL-12, IL-6, IFNg, IL-10, Mitogen activated protein kinase (MAPK)p38, phosphatidylinositol 3 (PI3) kinase, and nuclear factor Kappa B (NF-KB)

4 STUDY DESIGN

This is a phase I, open label clinical trial to evaluate the safety of *Lactobacillus rhamnosus* GG (LGG), ATCC 53103 in 10-15 elderly subjects. Enrollment of subjects is estimated to take approximately 3 months. Subject participation is approximately 3 months. The study will be conducted in elderly subjects at the Clinical Research Centers (CRC) at Tufts Medical Center and Massachusetts General Hospital. Subjects will be seen as outpatients. The study drug dose is $2x10^{10}$ LGG per day ($1x10^{10}$ LGG per capsule, 2 capsules per day). The study drug will be given orally twice a day for 28 days. The first dose will be ingested under observation in the CRC. Subjects will be evaluated during study visits at screening, Day 0 (baseline), Day 28 (+/- 2 days), and Day 56 (+/- 1 week), as well as on telephone calls on Days 3 (+/- 1 day), 7 (+/- 2 days), 14 (+/- 2 days).

Grade 2 or higher adverse events that were not present at baseline will be assessed using standardized questions asked during each study visit and telephone call, standardized physical examinations, and the results of laboratory tests. Subject diaries will be reviewed at each visit. Subjects will be reminded during the course of the study to contact the PI if any issue arises. Clinical laboratory tests will be performed at the CLIA-approved clinical laboratories at Tufts Medical Center and the Massachusetts General Hospital. Cultures of the stool and LGG capsules will be performed in Dr Snydman's research laboratory at Tufts Medical Center to assess for the presence of LGG. Bacterial DNA from nasopharyngeal and stool specimens will be processed in the CRC research laboratory for microbiota analysis.

A Data and Safety Monitoring Board (DSMB), see Section 9.7, will oversee the study. The DSMB will review the safety data approximately every six months. There will be no formal interim analysis. The DSMB will report on whether or not to continue the study after each review. The report will contain the following information regarding the study's safety to date:

- Overview of study status
- Summary of safety data for each subject through day 56
- Summary safety assessment

5 STUDY SCREENING AND ENROLLMENT

Subjects, aged 65-80 years, will be recruited from the greater Boston area. Strategies for recruitment include advertising in local newspapers, on web sites and subject databases using IRB-approved materials. It is anticipated that a total of 80 elderly subjects will be screened at both institutions (up to 40 at each institution) to achieve a sample size of 10-15 enrolled subjects. We expect that 50% of these will be female and about 20% will be minorities, based on the population distribution in Massachusetts. An IRB approved phone script will be used to pre-screen subjects who provide telephone numbers in response to advertisements. The script will provide a brief description of the study and ask the interested subject if they are in good general health, whether they consume yogurt or probiotics on a daily basis, whether they are interested in participating in the study and availability for the required follow-up period. Interpreters will be available as needed. Those who are interested will be scheduled for a screening visit in the CRC.

5.1 Screening

At the start of the screening visit, the Principal Investigator or designee will describe the study in detail and review the informed consent, Health Insurance Portability and Accountability Act (HIPAA) authorization and consent for HIV testing with the subject. Subjects are allowed as much time as they need to give informed, signed consent and willingness to proceed is reviewed at each visit. Screening numbers will be assigned by the Principal Investigator/designee. HIV testing is done according to the hospital's standard protocol.

Subjects who are screened and do not meet all entry criteria (screening failures) will be entered into a screening log. Descriptive data collected on screening failures will be entered into the clinical study database as recommended by the CONSORT statement [221].

5.2 Subject Inclusion Criteria

Subjects must meet all of the following inclusion criteria to be eligible to participate in the study:

- Age 65-80 years
- Willing to complete the informed consent process
- Able and willing to participate for the planned duration of the study, including availability for follow-up telephone contact
- Is community-dwelling for the past two years
- Has received routine physical in the past two years
- Has no new chronic conditions in the past two years

- Identifies a primary care clinician
- Has received recommended preventive services (Task Force for Clinical Preventive Services) for vaccination and cancer prevention/detection, e.g.;
    - Pneumococcal vaccination
    - Mammography
    - Screening colonoscopy for colon cancer
- Willing to comply with protocol and report on compliance and side effects during the study period
- Informed consent obtained and signed prior to screening.

5.3 Subject Exclusion Criteria

Subjects meeting any of the exclusion criteria at baseline will be excluded from the study.

- Consumption of supplements or food products containing LGG or probiotics for 28 days prior to the start of the study or consumption of yogurt that has the “live and active cultures” seal.
- Known or suspected allergies to probiotics, *Lactobacillus*, microcrystalline cellulose, gelatin, or antibiotics that may be used to treat LGG bacteremia or infection (i.e. subject able to tolerate at least 2 of the following regimens - Ampicillin or other beta lactam antibiotic, and Clindamycin, and Moxifloxacin).
- Received oral or parenteral antibiotics within 4 weeks of enrollment or prescribed antibiotics on the day of enrollment
- Drug or alcohol abuse within the previous 12 months
- Hospitalization, major surgery or endoscopy within the last 3 months
- Scheduled hospital admission within 3 months of enrollment
- Resident of a nursing home or rehabilitation center
- Presence of any of the following:
    - Grade 2 or higher abnormal vital signs or abnormalities on physical exam (Appendix A)
    - Indwelling catheter or implanted hardware/prosthetic device or feeding tube
    - Current or within the last 2 years, any episode of bowel leak, acute abdomen, diverticulitis, colitis, bloody bowel movements or peptic ulcer disease, including any surgical procedure or current prescription medications for any of these conditions
    - Current or within the last four weeks, active bowel disease such as an episode of infectious or non-infectious diarrhea, constipation, or vomiting lasting more than 12 hours or current prescription medications for any of these conditions

- Any history of gastric or intestinal dysmobility, slowed transit time, variable small intestinal permeability, pancreatitis, history of gastrointestinal tract cancer or metastasis, or inflammatory bowel disease or current prescription medications for any of these conditions
- Any history of Hepatitis B or Hepatitis C infections, cirrhosis, or chronic liver disease
- Underlying structural heart disease such as abnormal native heart valve or congenital abnormality, previous history of endocarditis or valve replacement, Stage IV congestive heart failure
- History of peripheral vascular disease or stroke
- Immunosuppression including HIV positive, solid organ or stem cell transplant recipient, receiving any oral or parenteral immunosuppressive therapy, neutrophil count <500/mm^3, or an anticipated drop in the neutrophil count to <500/mm^3 or active or planned chemotherapy or radiotherapy
- History of collagen vascular or autoimmune disease
- End stage renal disease
- History of chronic obstructive pulmonary disease or asthma
- Diabetes or thyroid disease
- Active tuberculosis (TB), defined as undergoing work up for suspected active TB infection or currently on treatment for active TB

- Positive drug or alcohol testing at screening or positive breathalyzer at baseline or an unwillingness to undergo drug and alcohol testing
- Abnormal laboratory tests defined as any of the following:
  - White blood cell (WBC) < 3.3 or > 12.0 K/µL
  - Platelets < 125 K/µL
  - Hemoglobin Males: < 12.0 g/dL; Females: < 11.0 g/dL
  - Creatinine > 1.8 mg/dL
  - Blood urea nitrogen (BUN) >27 mg/dL
  - Aspartate aminotransferase (AST) > 1.25 ULN
  - Alanine aminotransferase (ALT) > 1.25 ULN
  - Alkaline phosphatase > 2.0 ULN
  - Bilirubin (total) > 1.5 ULN
  - Glucose (non-fasting) >126 mg/dL
  - Positive HIV, Hepatitis B surface antigen or Hepatitis C antibody
- Any other condition that in the opinion of the investigator would jeopardize the safety or rights of the subject participating in the study or would make it unlikely the subject could complete the study

5.4 Enrollment

Subjects who meet all inclusion and have no exclusion criteria and who return for their baseline appointment are enrolled into the study. On enrollment, they will receive a unique study ID that will be used on all study forms and labels. Willingness to proceed is reviewed at each visit. In addition, subjects who enroll in this study will be informed of an optional sub study.

6 STUDY DRUG

6.1 Study Product Description

6.1.1 Acquisition

The study drug (LGG) will be supplied in blister packs by Amerifit Brands, Inc. Amerifit Brands, Inc. will ship the study drug via UPS overnight with ice packs to the research pharmacy at Tufts Medical Center and Massachusetts General Hospital. Upon receipt, the research pharmacist at Tufts Medical Center and Massachusetts General Hospital will inventory the contents of the shipment received, confirm the contents match with the shipping paperwork, and document the receipt of the shipment in log books to be maintained by the research pharmacy.

6.1.2 Formulation, Packaging, and Labeling

Lactobacillus rhamnosus GG, ATCC 53103 (LGG) is supplied in a gelatin capsule at a dosage of 1×10^{10} LGG per capsule. The capsule also contains microcrystalline cellulose (purified partially depolymerized cellulose), an inactive ingredient. Each capsule is wrapped in double foil to protect it against harmful light, air, and moisture and provided in blister packs. The blister packs are labeled with the lot number of the study drug prior to shipping to Tufts Medical Center and Massachusetts General Hospital.

6.1.3 Product Storage and Stability

The LGG capsules should be stored in a cool, dry place at or below 72-75°F. Although LGG capsules do not require refrigeration, they are more stable when stored under refrigeration. The stability of the capsule contents will be assessed at predetermined time points as described in Section 13.

6.2 Dosage, Preparation, and Administration of Study Drug

The dose of LGG for this study is 1×10^{10} LGG administered twice a day for 28 days. This dose has been widely used in studies of LGG described in Tables 1-3 above. When an order for study drug is received, the research pharmacist will retrieve the LGG capsules in the blister packs and place them in a plastic bag. The plastic bag is labeled with the following information:

- Subject study ID
- Name of subject
- Name of study/protocol number
- MD prescribing the study drug

- Directions
- Quantity dispensed
- Dispensing date
- “Study Drug”
- Expiration date
- Storage requirements “Keep at or below room temperature”

The study drug will be sent to the CRC where research study staff will dispense it to the subject, along with clear instructions on proper use of the study drug. An instruction sheet on taking the LGG will be given to all subjects and the first dose will be administered under direct observation in the CRC.

6.3 Accountability Procedures for the Study Drug

The study pharmacist and/or study coordinator will keep source documentation for accountability of study drug that details receipt, dispensing, and return of used and unused study drug. Study drug will be inventoried and accounted for throughout the study. All study drugs will be stored in locked facilities until they are either returned to Amerifit Brands, Inc. at the closure of the study or at an earlier time point if requested, or destroyed by the pharmacy. Prior to dispensing, blister packs containing study drug will be visually inspected. If a blister pack appears to have tears or signs of tampering, the blister pack will be rejected for use and retained until the end of the study. At the end of the study, Amerifit Brands, Inc. will determine dispensation of all remaining unused blister packs at the study site. An accountability log will be kept, indicating the final disposition of study drug. This log will be provided to the Principal Investigator at the end of the study.

6.4 Assessment of Subject Compliance with Study Drug

At the 28 day study visit, subjects will be asked to bring in all remaining study drug. Study staff will count and record the number of study drug capsules brought back.

6.5 Concomitant Therapy

All medications taken within 30 days prior to the administration of study drug and all concomitant medications administered during the study are to be recorded on the relevant case report form page(s), along with the reason for use. All prior hospitalizations as well as all surgeries will be recorded on the relevant CRF page(s). Subjects using LGG or other probiotics (including yogurt displaying the “live and active cultures” seal) within 28 days will be excluded from participating in the study, unless they are willing to discontinue these products for 28 days before their baseline visit and for the duration of the study. Those who consume yogurt will be eligible only if they

consume yogurt brands sold in the United States that are heat-treated, thus have active cultures destroyed. During the study period, subjects will be asked to avoid consuming brands of yogurt that display the “live and active cultures” seal (as listed by the National Yogurt Association’s seal program – www.aboutyogurt.com/lacYogurt).

7 STUDY SCHEDULE

The schedule of evaluations and procedures that must be performed at specific time points is described in the following sections and is summarized in the Time and Event Schedule (page 11).

7.1 Screening (Visit Day -31 to Day -1)

Potential subjects for this study will be scheduled for a screening visit. At the start of the visit, the nature of the study will be explained to him/her by the study investigator or designee, and the potential subject will be asked to give written informed consent and sign the HIPAA authorization. Informed consent/HIPAA authorization must be obtained prior to any procedures occurring. Subjects will be asked to describe their medical history (including a review of all inclusion and exclusion criteria), and have a physical examination (with vital signs) and laboratory tests. HIV testing will be performed according to the Tufts Medical Center's and Massachusetts General Hospital standard procedure, after the subject has signed the Tufts Medical Center or Massachusetts General Hospital standard HIV testing consent form. The specific procedures during the screening visit include:

- Demographics, medical history and review of inclusion and exclusion criteria, concomitant therapy/medications, consumption of probiotics, yogurt, etc.
- Physical exam with height, weight and vital signs
- Routine Laboratory tests
  - CBC
  - Serum chemistries/liver function tests
  - Drug and alcohol toxicity screen
  - Serology for anti-HIV, anti-HCV, and Hepatitis B surface antigen (HbsAg)

Subjects are provided with information on foods and probiotic products to avoid during the study, a stool sample collection kit and instructions on how to collect stool specimens should they be eligible to participate in the study.

7.2 Enrollment (Baseline Visit, Day 0)

Subjects who are eligible to participate in the study after all of the screening tests have been completed will return for a baseline visit. The following evaluations and procedures will be done at the baseline visit, prior to receiving the study drug:

- Interval medical history, concomitant therapy/medications, consumption of probiotics, yogurt, etc and review of symptoms

- Physical exam with weight and vital signs
- Routine Laboratory tests
  - CBC
  - Serum chemistries/liver function tests
- Breathylzer - Alcohol screen
- Research Laboratory tests
  - Collection of nasopharyngeal specimens for microbiota
  - Collection of stool sample for microbiota and LGG culture

Enrolled subjects will be offered an opportunity to participate in the sub-study. After they either decline or accept, study drug will be dispensed.

- Study drug cultures for colony counts
- First administration of study drug. Subject will learn how to take the study drug twice daily for the next 4 weeks.
- Sub-study only – blood for DNA, mRNA and cytokines

Subjects will be reminded to contact the PI at any time for any study related issues or adverse events that occur. Subjects will receive a new stool container and again be instructed on how to collect the stool.

Telephone Calls Days 3, 7, and 14

Volunteers will also be telephoned on Days 3 (+/- 1 day), 7 (+/- 2 days), and 14 (+/- 2 days), to inquire about adverse events and discuss study drug use. A standardized form that asks the same questions that are listed in the diary (Appendix C) will be developed for this purpose. Open-ended questions will also be asked to solicit adverse events. The volunteer will be reminded to call the PI or study staff at any time to discuss any questions or changes in health status.

7.3 Follow-up (Day 28 (End of Treatment), Day 56 (End of Study))

Study subjects will return for follow-up evaluations to the clinical study site on study day 28 and 56. The following evaluations and procedures will occur at each visit.

- Interval medical history, concomitant therapy/medications, consumption of probiotics, yogurt, etc and adverse event questionnaire, self report and diary review
- Physical exam with weight and vital signs
- Routine Laboratory tests
  - CBC

  - Serum chemistries/liver function tests
- Research Laboratory tests
  - Collection of nasopharyngeal specimens for microbiota
  - Collection of stool sample for microbiota and LGG culture
  - Sub-study only – blood for DNA, mRNA and cytokines
- Study drug cultures for colony counts (day 28 only)

At the follow-up visits, subjects will be reminded to contact the PI at any time for any issues or adverse events that occur. On day 28, subjects will receive a new stool container and again be instructed on how to collect the stool.

7.4 Early Termination Visit

Subjects are free to withdraw from participating in the study at any time upon request. The reason for withdrawal will be documented on the CRF. If a subject withdraws early due to an adverse event, he/she will be followed until resolution/stabilization of the adverse event. Subjects will be withdrawn if they are hospitalized.

If a subject prematurely withdraws from the study or is withdrawn from the study, the same procedures and evaluations will be performed as in the final study visit if possible at the time of withdrawal from the study (i.e. study subject withdraws at time of a visit and consents to having procedures/evaluations done):

- Interval medical history, concomitant therapy/medications, consumption of probiotics, yogurt, etc and adverse event questionnaire, self report and diary review
- Physical exam with weight and vital signs
- Routine Laboratory tests
  - CBC
  - Serum chemistries/liver function tests
- Research Laboratory tests
  - Collection of nasopharyngeal specimens for microbiota
  - Collection of stool sample for microbiota and LGG culture
  - Sub-study only – blood for DNA, mRNA and cytokines

8 STUDY PROCEDURES/EVALUATIONS

Dr Hibberd and her study team are responsible for ensuring that all study procedures and evaluations are performed.

8.1 Clinical Evaluations

- Medical history from subject interviews
  - Medical history, current conditions, review of symptoms
  - Drug allergies
  - Concomitant medications
- Physical examination
  - Vital signs, height, weight
  - Exam of body systems – HEENT, neck, heart, lungs, abdomen, skin, musculoskeletal, neurologic, lymph nodes, vascular, other

8.2 Laboratory Evaluations

8.2.1 Clinical Laboratory Evaluations

The following clinical laboratory evaluations will be performed by the Tufts Medical Center or Massachusetts General Hospital CLIA-approved clinical laboratories.

- Hepatitis panel and HIV (screening) - HCV antibody, HBsAg, HIV antibody.
- Drug and alcohol toxicity screen
- CBC – hemoglobin, WBC with differential, platelet count.
- Serum chemistries/liver function tests – alkaline phosphatase, AST, ALT, total bilirubin, BUN, creatinine, glucose.

8.2.2 Special Assays or Procedures

8.2.2.1 Culture of LGG

All cultures for LGG will be processed and performed in Dr Snydman's research laboratory at Tufts Medical Center using standard culture methods.

8.2.2.2 Microbiota

All samples for microbiota will be processed in preparation for bacterial RNA/DNA extraction by homogenizing the specimen with RNALater (Qiagen, Valencia CA) and then stored at – 80 C in multiple microfuge tubes. The batched samples will have purified bacterial DNA/RNA extracted and will be amplified using PCR. The PCR products will be pooled and pyrosequenced. Our current procedures are as follows, although these may be modified at the time that the batched analyses are run, due to likely scientific advances. Homogenates of stool will be thawed, and DNA will be extracted using a ZYMO Fecal DNA Kit (Zymo Research, CA). The purified DNA will be stored at -80°C in the tissue bank for future batched pyrosequencing of 16S rRNA genes at the University of Maryland's Institute of Genomic Sciences (IGS). Purified DNA, labeled only with study ID, specimen type and visit identifier, will be subjected to PCR to generate amplicons of 16S rRNA genes for pyrosequencing by protocols established by IGS. The forward primer for all stool DNA samples will be the 16S rRNA gene primer 454_27F (GCCTTGCCAGCCCGCTCAGTCAGAGTTTGATCCTGGCTCAG, Sigma-Aldrich, Inc, St. Louis MO) [222]. The reverse primer for each sample will be constructed to contain a unique tag sequence to serve as a "barcode" to identify its origin. After amplification, the PCR products will pooled and pyrosequenced at IGS on a Roche/Life Sciences 454 Pyrosequencer (Branford, CT). To evaluate possible contamination of reagents and subsequent amplification of foreign DNA during PCR amplification, control reactions that contain reagents but no DNA template will be included in each PCR run. If this negative control shows positive amplification, the PCR will be repeated with fresh reagents. To limit contamination, all DNA handling will be performed in a dedicated hood, barrier tips will be used on pipettes to avoid cross-contamination, and gloves will be changed regularly. To help control for PCR-induced biases [223], quantitative PCR will be used to find the amplification saturation point for each sample. During the PCR amplification, the minimum number of cycles will be used to reach the amount of DNA product needed for pyrosequencing. The procedures for the nasopharyngeal samples are the same except that DNA will be extracted using the DNA Mini Kit (Qiagen, Valencia, CA). Samples are only identified with a study ID number, specimen type and study visit descriptor.

8.2.2.3 Immune Response Genes

DNA and RNA will be extracted from blood using the Paxgene kits and will be stored for future analysis of immune response genes and immune response gene products such as cytokines as follows. The subject's RNA will be extracted from 5 mL of whole blood per visit. The RNA will be stored at -80 C for later batched analysis of RNA gene expression profiles in Dr Solano-Aguilar's laboratory at the USDA-ARS in Beltsville, Maryland. Its quality and quantity will be analyzed by the Experion automated electrophoresis system (Biorad) and equal RNA amounts (10 micrograms) per each sample will be used for first strand cDNA synthesis using Superscript II Reverse Transcriptase (Invitrogen) [224]. Cytokine production in response to bacterial stimulation will be analyzed using real time PCR by following the kinetics of mRNA expression of pro-inflammatory cytokine genes (i.e. TNF-a, IL-12, IL-6, IFNg), anti-inflammatory genes (IL-10) and different signaling pathways (i.e. Mitogen activated protein kinase (MAPK) p38, phosphatidylinositol 3 (PI3) kinase, and nuclear

factor Kappa B (NF-KB) involved in probiotic-induced cytokine production as previously described in vitro [225]. Two grams of stool per visit will also be stored at -80 C and provided to Dr Solano-Aguilar to enable her to extract bacterial DNA from the specimens. Samples are only identified with a study ID number, specimen type and study visit descriptor.

9 ASSESSMENT OF SAFETY

9.1 Subject Evaluations

Study staff will inquire about symptoms (anticipated adverse events) using standardized and opened ended questions and at each study visit. AEs will also be solicited during telephone calls on study day 3, 7, and 14. In addition, routine physical examinations and safety labs will be assessed at all study visits.

Subjects will also be asked to keep a daily symptom diary. Subjects will be instructed on how to complete this diary at the baseline visit. Subjects will document all symptoms experienced and the intensity of the symptoms on a daily basis. The symptom diary will be reviewed at all study visits. The diary will also be reviewed during scheduled telephone calls on Days 3 (+/- 1 day), 7 (+/- 2 days) and 14 (+/- 2 days). A phone script will be utilized for this purpose during these scheduled calls. If any symptoms have been recorded in the diary and are reported during the scheduled telephone call, a study physician will follow-up with the subject by phone. The study physician will then determine how to rate the event based on his/her evaluation and will arrange for appropriate follow-up and treatment, if necessary. An additional study visit may be scheduled. The study physician will complete the appropriate AE form for all adverse events identified during the scheduled phone calls and study visits, as well as during any unscheduled calls or visits.

9.2 Subject Safety Information

A list of risks is presented to the subject in the consent form, along with contact information to reach an investigator in case of a perceived adverse event or side effect. Subjects are given a copy of the consent form and instructed to keep this for the duration of the study.

9.3 Availability of the Investigator

Dr Hibberd, the Principal Investigator, or her designee is available 24 hours a day, seven days a week for study-related questions at both Tufts Medical Center and Massachusetts General Hospital. In the event that she is not available by cell phone or pager, her pager number will be forwarded to another investigator who will be familiar with the study protocol and has Dr Hibberd's contact information.

9.4 Adverse Events

9.4.1 Definitions

An **adverse event (AE)** is any undesirable experience associated with the use of a medical product in a subject (http://www.fda.gov/medWatch/report/DESK/advevnt.htm). An AE can therefore be any

unfavorable and unintended sign (including an abnormal laboratory finding), symptom, or disease temporally associated with the use of a study drug. Study subjects will be monitored for the occurrence of events from the day of enrollment for a maximum period of 2 months, although intense monitoring of adverse events will occur during the first 28 days when the subject is taking LGG. The occurrence of an AE may come to the attention of study staff during study visits and interviews with a study subject presenting for medical care, or upon review by a study monitor. In addition, subjects will be evaluated in person or by telephone and asked about unanticipated adverse events. We will use both open-ended questions and specific questions about possible adverse effects, such as presence of diarrhea and other abdominal symptoms.

9.4.2 Recording of Adverse Events

Each adverse event will be recorded on an Adverse Event Case Report Form which will include the following information:

- Description of symptoms and/or event
- Onset and Duration, including intermittent or not
- Adverse Event Severity (including determination if the event qualifies as a Serious Adverse Event – see Section 9.4.2.1 below)
- Assessment of relationship between the study drug and adverse event (see Section 9.4.2.2 below)
- Action(s) taken to treat the adverse event
- Outcome

Once identified, all AEs (including serious) will be followed until resolved or until the PI considers that the subject is stable. Evaluation of AEs will be done during follow-up visits, as well as during unscheduled visits, and scheduled telephone calls. All communications, examinations, and testing that occurs as a result of the AE will be clearly documented on AE forms and the appropriate CRFs. Changes in the severity of an AE will be documented to allow an assessment of the duration of the event at each level of intensity to be performed.

If events occur which raise questions about the safety of continued administration of LGG, the subject’s physician will have the option to withdraw the subject from the study.

9.4.2.1 Adverse Event Severity

All AEs will be assessed by the clinician and classified according to the **FDA’s Guidance for Industry: Toxicity Grading Scale for Healthy Adult and Adolescent Volunteers Enrolled in Preventive Vaccine Clinical Trials** as recommended by FDA. See Appendix A. As per the FDA’s recommendation, we will use the following grading scale to classify adverse events:

- Mild (Grade 1): an event which requires no treatment and does not interfere with the subject's daily activities.
- Moderate (Grade 2): an event which may cause some interference with the subject's daily activity but does not require medical intervention
- Severe (Grade 3): an event which prevents usual daily activity (incapacitating; unable to perform usual activities; requires bed rest or absenteeism) and requires medical intervention.
- Potentially life threatening (Grade 4): an event which results in an ER visit or hospitalization

A **serious adverse event (SAE)** is defined as an AE that meets one of the following conditions (http://www.fda.gov/medWatch/report/DESK/advevnt.htm)

- Death – Report if the subject's death is suspected as being a direct outcome of the adverse event
- Life-Threatening – Report if the subject was at substantial risk of dying at the time of the adverse event or it is suspected that the use or continued use of the product would result in the subject's death.
- Hospitalization (initial or prolonged) – Report of admission to the hospital or prolongation of a hospital stay results because of the adverse event.
- Disability – Report if the adverse event resulted in a significant, persistent, or permanent change, impairment, damage or disruption in the subject's body function/structure, physical activities or quality of life.
- Congenital Anomaly – Report if there are suspicions that exposure to a medical product prior to conception or during pregnancy resulted in an adverse outcome in the child.
- Requires Intervention to Prevent Permanent Impairment or Damage – Report if you suspect that the use of a medical product may result in a condition which required medical or surgical intervention to preclude permanent impairment or damage to a subject.

9.4.2.2 Assessing the Relationship between Study Drug and Adverse Event

The principal investigator will classify the relationship of the study protocol to the adverse event as follows:

- Not related: The event is clearly related to factors such as the subject's clinical state, not to therapeutic interventions associated with the study protocol.
- Remote: The event was most likely related to factors such as the subject's clinical state, not to therapeutic interventions associated with the study protocol.
- Possible: The event follows a reasonable temporal sequence from consuming LGG, but is possibly related to factors such as the subject's clinical state.

- Probable: The event follows a reasonable temporal sequence from consuming LGG and cannot be reasonably explained by factors such as the subject's clinical state.
- Highly Probable: The event follows a reasonable temporal sequence from consuming LGG, and cannot be reasonably explained by factors such as the subject's clinical state. In addition, the event occurs immediately following ingestion or application of study drug or reappears on repeat exposure, if the PI considers it safe to re-expose the subject to study drug.

9.4.3 Reporting of Adverse Events

All serious adverse events and new Grade 3 or 4 toxicities reported by the subject or detected and reported to any study physician will be classified as above and recorded on an Adverse Event Form and in the source document. The causal relationship will be evaluated by the Principal Investigator and the relationship of the event to the study drug will be reported to the IRBs and the chair of the DSMB within 72 hours and to the NIH and FDA.

Other adverse events will be summarized approximately quarterly for the DSMB and the IRB.

9.5 Withdrawal of Subjects

The study drug will be discontinued and the subject withdrawn from the study if any of the following occur:

- An adverse event, intercurrent illness, or other medical condition or situation occurs such that continued participation in the study would not be in the best interest of the subject
- If an enrolled subject begins immunosuppressive medication or events occur which raise questions as to the safety of continued administration of LGG, and/or the subject's primary care physician wishes to withdraw the subject from the study
- Grade 3 or Grade 4 gastrointestinal side effects
- Lactobacillus bacteremia or invasive *Lactobacillus* infection
- Subject withdraws consent
- Subject is lost to follow-up

9.6 Rescue Medication

There is a theoretical risk of translocation of LGG across the bowel wall and resultant clinical infection caused by Lactobacillus. Possible infections include bacteremia, endocarditis, or abscesses. The antibiotic susceptibilities of LGG, as reported by various researchers, are attached (Appendix C). Based on the available literature, including experimental models of infective endocarditis with *Lactobacillus plantarum* [226], we would recommend treatment of *Lactobacillus* endocarditis with a beta-lactam and gentamicin, but bacteremia alone or other sites of infection may be treatable with a beta lactam only [176, 226, 227]. If any study subject develops invasive disease

(bacteremia, endocarditis, etc.), we will recommend this treatment course, but will also ask that an independent infectious disease physician (not involved in the study) evaluate and treat the subject. We will also obtain *in vitro* sensitivity data on the specific isolate as several articles show a benefit of treating with antibiotics to which the organism is sensitive *in vitro* [176, 226].

The dose, route, frequency, and duration of treatment will be determined by the infectious disease consultant caring for the subject but the recommended regimens are as follows:

Ampicillin 2 grams intravenously every 6 hours (or alternative beta-lactam antibiotic) for 14 days or
Clindamycin 900 mg intravenously every 8 hours for 14 days or
Moxifloxacin 400 mg intravenously every 24 hours for 14 days

Alternative oral regimens for these 3 drugs should the subject be able to transition to an oral regimen to complete the 14 days of therapy are as follows:

Ampicillin 500 mg orally every 6 hours (or alternative beta-lactam antibiotic) for 14 days or
Clindamycin 300 mg orally every 8 hours for 14 days or
Moxifloxacin 400 mg orally once a day for 14 days

At the conclusion of therapy for invasive disease, we will obtain throat and stool cultures to determine whether the subject is colonized with LGG in either of these locations. The *in vitro* susceptibility pattern of the isolate will be rechecked to guide additional antibiotic therapy to eradicate the LGG. Subjects will be followed until LGG is no longer isolated from these potential colonizing sites.

9.7 Safety Oversight and Study Termination

NIH or the Principal Investigator will appoint a Data and Safety Monitoring Board (DSMB). The DSMB is responsible for monitoring the project for subject safety and adequacy of data quality. The DSMB will advise the Principal Investigator. If the DSMB recommends a study change for subject safety or ethical reasons, or if the study is closed early due to slow accrual, the Principal Investigator will be responsible for implementing the recommendations as expeditiously as possible, according to standard policies of NIH. If the PI does not concur with the recommendations of the DSMB, the NIH program office, Principal Investigator, and DSMB chair will be responsible for reaching a mutually acceptable decision according to usual practices. The DSMB will meet approximately every 6 months (either in person or by conference call) during the study and more frequently as needed. The DSMB will consist of at least 3 members: two physicians (at least one infectious disease specialist) and a statistician. Decisions will be made by majority vote. The DSMB members will receive reports of all Grade 1-4 toxicities throughout the conduct of the study. The DSMB will make recommendations to the PI based on their analysis of the reports.

The study may be terminated at any time, including at a DSMB interim safety review. If a Grade 4 or serious AE occurs (see Appendix A and Section 9.4.1.1) and the event is judged to be probably or definitely related to having received the study drug, the study will be immediately suspended by the Principal Investigator pending review of all appropriate safety data. The event will be reported to the DSMB, IRB and FDA within 72 hours of notification of its occurrence. No additional subjects will receive the study drug depending on the joint decision of the DSMB and Principal Investigator as to whether further doses can be given or the entire trial should be terminated.

DSMB members will be asked to review our assessment of the likely relatedness of the Grade 4 or SAE to the study drug, and if it is considered probably or definitely related, the event will be considered an SAE that will result in stopping of the study.

10 CLINICAL MONITORING

10.1 Study Monitoring Plan

The CRCs in both institutions have procedures in place to verify consent and HIPAA requirements at each visit. During the study, the FDA may monitor the clinical site at its discretion to check the progress of enrollment, verify the presence of informed consent and HIPAA authorization, check adherence to the inclusion/exclusion criteria, monitor completeness of study subjects' records and accuracy of entries on the CRFs, review adherence to the protocol and to Good Clinical Practice, verify the study drug is being stored, dispensed, and accounted for according to specification, and review documentation of serious adverse events and the recording of safety variables. The clinical monitoring plan document in the Manual of Operations will include details describing who will conduct the monitoring, at what frequency monitoring will be done, and what level of detail monitoring will be conducted (the number of subject charts to be reviewed, which/what proportion of data fields will be monitored, and what will be monitored), and who will be responsible for ensuring that monitoring findings are addressed. No information in these records about the identity of the study subject will leave the clinical study site.

11 STATISTICAL METHODS

11.1 Sample Size Considerations

The sample size for this study is not determined from power analysis. The sample size of 10-15 adults was recommended by the FDA.

11.2 Interim Analysis

There are no formal statistical interim analyses for this Phase I study. Interim safety data will be provided to the DSMB approximately every 6 months.

11.3 Statistical Analysis

11.3.1 Safety

Adverse events reported by subjects who receive at least one dose of the study will be summarized by body system and relationship to study product. The rate of Grade II or higher vital signs, physical examinations and laboratory tests will be calculated and summarized using descriptive statistics. Concomitant medications and significant non-drug therapies will also be reported. The precise type of missing data, should it occur, will be described.

11.3.2 Microbiota Richness and Diversity

Richness will be reported as the number of operational taxonomic units. Diversity will be reported as the Shannon Diversity Index. Additional graphical output such as heat maps will be generated.

11.3.3 Cytokine Production

We will describe the time course of mRNA production of TNF-a, IL-12, IL-6, IFNg, IL-10) and different Mitogen activated protein kinase (MAPK)p38, phosphatidylinositol 3 (PI3) kinase, and nuclear factor Kappa B (NF-KB), over time.

12 SOURCE DOCUMENTS AND ACCESS TO SOURCE DATA/DOCUMENTS

Medical and research records will be maintained for this study in compliance with ICH E6, Section 4.9, and regulatory and institutional requirements for the protection of confidentiality of research subjects. Study staff listed in the consent form will have access to records. Authorized representatives of NIH and regulatory agencies may examine (and when required by applicable law, to copy) clinical records for the purposes of quality assurance reviews, audits, and evaluation of the study safety and progress.

Source documents for this study contain demographic and medical information, and a copy of the signed Informed Consent /HIPAA Authorization. Clinical lab test results will also be the source documents for this study.

13 QUALITY CONTROL AND QUALITY ASSURANCE

The quality of the research will be assured by the following:

- Developing Standard Operating Procedures (SOPs) that detail how the research team will ensure that data are generated, documented, and reported in compliance with the protocol, GCP, and the applicable regulatory requirements (refer to Section 10). These will include:
  - Accountability procedures for the study drug to clearly outline responsibilities and expectations (Refer to Section 6.3)
  - Procedures for ensuring that the study drug being used contains the appropriate amount of active LGG throughout the study.
  - A study schedule and study procedures to clearly outline what happens at each stage of the study (Refer to Section 7 and Section 8)
  - A plan to evaluate safety which includes safety parameters to be evaluated and methods and timing for assessing and recording safety data (Refer to Section 9)
  - Internal site monitoring procedures to identify problems quickly so they can be resolved and prevented from occurring in the future
  - Data handling/record keeping procedures to clearly outline how data are recorded, who is responsible for various data management tasks, how often summary reports are written, how protocol deviations are handled (Refer to Section 15)
    - Applying quality control to each stage of data handling to ensure that all data are reliable and have been processed correctly (Refer to Section 15)
    - Appointing a Data Safety Monitoring Board to assess the progress of the trial, including safety data (Refer to Section 9.7)
    - Developing a training program for study staff to ensure that each member of the study team has the knowledge base to effectively/accurately carry out study responsibilities

14 ETHICS/PROTECTION OF HUMAN SUBJECTS

14.1 Ethical Standard

The investigator will ensure that this study is conducted in full conformity with the principles set forth in The Belmont Report: Ethical Principles and Guidelines for the Protection of Human Subjects of Research of the US National Commission for the Protection of Human Subjects of Biomedical and Behavioral Research (April 18, 1979) and codified in 45 CFR Part 46 and/or the ICH E6; 62 Federal Regulations 25691 (1997).

14.2 Institutional Review Board

Before implementing this study, the protocol, the proposed Informed Consent/HIPAA Authorization, and other information to study subjects must be reviewed by the Institutional Review Board (IRB). A signed and dated statement that the protocol and Informed Consent/HIPAA authorization have been approved by the IRB must be obtained before study initiation. Any amendments to the protocol which need formal approval as required by federal law will be approved by this committee. The IRB will also be notified for all other amendments (i.e. administrative changes).

14.3 Informed Consent Process

Informed consent is a process that is initiated prior to the subject agreeing to participate in the study and continuing throughout the subject's study participation. Extensive discussion of risks and possible benefits of this therapy will be provided to the subjects and their families. Consent forms describing in detail the study drug, study procedures, and risks are given to the subject and written documentation of informed consent is required prior to administering study drug. Consent forms will be IRB-approved and the subject will be asked to read and review the document. Upon reviewing the document, the investigator will explain the research study to the subject and answer any questions that may arise. The subjects will sign the informed consent document prior to any procedures being done specifically for the study. The subjects should have the opportunity to discuss the study with their surrogates or think about it prior to agreeing to participate. The subjects may withdraw consent at any time throughout the course of the trial. A copy of the informed consent document will be given to the subjects for their records. The rights and welfare of the subjects will be protected by emphasizing to them that the quality of their medical care will not be adversely affected if they decline to participate in this study.

In addition to the research consent form, a consent form for HIV testing will also be used in this study. This HIV testing consent form has been approved by Tufts Medical Center or Massachusetts General Hospital.

This study includes an optional sub-study. Subjects who enroll in the study will be given an opportunity to accept or decline participation in the optional sub-study. They are not required to participate in the sub-study.

14.3.1 Informed Consent/Assent Process (in Case of a Minor)

Not applicable.

14.4 Exclusion of Women, Minorities, and Children (Special Populations)

Children will be excluded in this study, as this study is intended to examine the safety of LGG in elderly subjects.

14.5 Subject Confidentiality

Subject confidentiality is strictly held in trust by the participating investigators and their staff. This confidentiality is extended to cover testing of biological samples and genetic tests in addition to the clinical information relating to participating subjects.

15 DATA HANDLING AND RECORD KEEPING

The investigator is responsible to ensure the accuracy, completeness, legibility, and timeliness of the data reported. All instructions regarding completing forms, data handling procedures, and procedures for data monitoring will be provided in the Standard Operating Procedures. All source documents should be completed in a neat, legible manner to ensure accurate interpretation of data. When making changes or corrections, the original entry will be crossed out with a single line, and the change will be initialed and dated. ERASING, OVERWRITING, OR USING CORRECTION FLUID OR TAPE ON THE ORIGINAL will not be permitted.

15.1 Data Management Responsibilities

All source documents and laboratory reports must be reviewed by the clinical team and data entry staff, who will ensure that they are accurate and complete. Adverse events must be graded, assessed for severity and causality, and reviewed by the PI or designee.

Data collection is the responsibility of the clinical trial staff under the supervision of the PI. During the study, the investigator must maintain complete and accurate documentation for the study.

Dr Hibberd's group will assume responsibilities for data management and quality review, and Ms Anne-Maria Fiorino will perform the statistical analysis and report the study data.

15.2 Types of Data

Data for this study will include safety, laboratory, and outcome measures (e.g. safety). Study data will be collected on study CRFs and entered into the study data base. Safety reports will be presented to the DSMB approximately every 6 months.

15.3 Study Records Retention

Study documents will be retained for a minimum of 2 years.

15.4 Protocol Deviations

A protocol deviation is any noncompliance with the clinical trial protocol, Good Clinical Practice (GCP), or Manual of Procedures requirements. The noncompliance may be either on the part of the investigator or the study site staff. As a result of deviations, corrective actions are to be developed by the site and implemented promptly.

These practices are consistent with ICH E6:

- o Compliance with Protocol, Sections 4.5.1, 4.5.2, and 4.5.3
- o Quality Assurance and Quality Control, Section 5.1.1
- o Noncompliance, Sections 5.20.1, and 5.20.2.

It is the responsibility of the site's study staff to use continuous vigilance to identify and report deviations promptly after identification of the protocol deviation to Dr Hibberd and the Tufts Medical Center and Massachusetts General Hospital IRBs per their guidelines. All deviations from the protocol must be addressed in subject source documents. A completed copy of the Protocol Deviation Form must be maintained in the regulatory file, as well as in the subject's source document. The site PI/study staff is responsible for knowing and adhering to their IRB requirements.

16 TRIAL REGISTRATION AND PUBLICATION POLICY

This trial will be registered on Clinicaltrials.gov, which is sponsored by the National Library of Medicine. The PI will publish results of this research in a scientific journal.

Reference List

1. Couch RB. Advances in influenza virus vaccine research. Ann N Y Acad Sci 1993;685:803-812.
2. Thompson WW, Shay DK, Weintraub E et al. Mortality associated with influenza and respiratory syncytial virus in the United States. JAMA 2003;289(2):179-186.
3. Thompson WW, Shay DK, Weintraub E et al. Influenza-associated hospitalizations in the United States. JAMA 2004;292(11):1333-1340.
4. Simonsen L, Clarke MJ, Schonberger LB, Arden NH, Cox NJ, Fukuda K. Pandemic versus epidemic influenza mortality: a pattern of changing age distribution. J Infect Dis 1998;178(1):53-60.
5. Simonsen L, Fukuda K, Schonberger LB, Cox NJ. The impact of influenza epidemics on hospitalizations. J Infect Dis 2000;181(3):831-837.
6. Harper SA, Fukuda K, Uyeki TM, Cox NJ, Bridges CB. Prevention and control of influenza: recommendations of the Advisory Committee on Immunization Practices (ACIP). MMWR Recomm Rep 2004;53(RR-6):1-40.
7. Govaert TM, Thijs CT, Masurel N, Sprenger MJ, Dinant GJ, Knottnerus JA. The efficacy of influenza vaccination in elderly individuals. A randomized double-blind placebo-controlled trial. JAMA 1994;272(21):1661-1665.
8. Voordouw AC, Sturkenboom MC, Dieleman JP et al. Annual revaccination against influenza and mortality risk in community-dwelling elderly persons. JAMA 2004;292(17):2089-2095.
9. Bridges CB, Thompson WW, Meltzer MI et al. Effectiveness and cost-benefit of influenza vaccination of healthy working adults: A randomized controlled trial. JAMA 2000;284(13):1655-1663.
10. Nichol KL, Mendelman PM, Mallon KP et al. Effectiveness of live, attenuated intranasal influenza virus vaccine in healthy, working adults: a randomized controlled trial. JAMA 1999;282(2):137-144.
11. Edwards KM, Dupont WD, Westrich MK, Plummer WD, Jr., Palmer PS, Wright PF. A randomized controlled trial of cold-adapted and inactivated vaccines for the prevention of influenza A disease. J Infect Dis 1994;169(1):68-76.
12. Beyer WE, Palache AM, de Jong JC, Osterhaus AD. Cold-adapted live influenza vaccine versus inactivated vaccine: systemic vaccine reactions, local and systemic antibody response, and vaccine efficacy. A meta-analysis. Vaccine 2002;20(9-10):1340-1353.
13. Monto AS, Ohmit SE, Petrie JG et al. Comparative efficacy of inactivated and live attenuated influenza vaccines. N Engl J Med 2009;361(13):1260-1267.
14. Ohmit SE, Victor JC, Rotthoff JR et al. Prevention of antigenically drifted influenza by inactivated and live attenuated vaccines. N Engl J Med 2006;355(24):2513-2522.
15. De Villiers PJ, Steele AD, Hiemstra LA et al. Efficacy and safety of a live attenuated influenza vaccine in adults 60 years of age and older. Vaccine 2009;28(1):228-234.
16. Gorse GJ, Campbell MJ, Otto EE, Powers DC, Chambers GW, Newman FK. Increased anti-influenza A virus cytotoxic T cell activity following vaccination of the chronically ill elderly with live attenuated or inactivated influenza virus vaccine. J Infect Dis 1995;172(1):1-10.
17. Gorse GJ, Otto EE, Powers DC, Chambers GW, Eickhoff CS, Newman FK. Induction of mucosal antibodies by live attenuated and inactivated influenza virus vaccines in the chronically ill elderly. J Infect Dis 1996;173(2):285-290.

18. Gorse GJ, O'Connor TZ, Young SL et al. Efficacy trial of live, cold-adapted and inactivated influenza virus vaccines in older adults with chronic obstructive pulmonary disease: a VA cooperative study. Vaccine 2003;21(17-18):2133-2144.
19. Gorse GJ, O'Connor TZ, Newman FK et al. Immunity to influenza in older adults with chronic obstructive pulmonary disease. J Infect Dis 2004;190(1):11-19.
20. Jackson LA, Holmes SJ, Mendelman PM, Huggins L, Cho I, Rhorer J. Safety of a trivalent live attenuated intranasal influenza vaccine, FluMist, administered in addition to parenteral trivalent inactivated influenza vaccine to seniors with chronic medical conditions. Vaccine 1999;17(15-16):1905-1909.
21. Rudenko LG, Arden NH, Grigorieva E et al. Immunogenicity and efficacy of Russian live attenuated and US inactivated influenza vaccines used alone and in combination in nursing home residents. Vaccine 2000;19(2-3):308-318.
22. Stepanova L, Naykhin A, Kolmskog C et al. The humoral response to live and inactivated influenza vaccines administered alone and in combination to young adults and elderly. J Clin Virol 2002;24(3):193-201.
23. Treanor JJ, Mattison HR, Dumyati G et al. Protective efficacy of combined live intranasal and inactivated influenza A virus vaccines in the elderly. Ann Intern Med 1992;117(8):625-633.
24. Treanor JJ, Betts RF. Evaluation of live, cold-adapted influenza A and B virus vaccines in elderly and high-risk subjects. Vaccine 1998;16(18):1756-1760.
25. Brokstad KA, Cox RJ, Eriksson JC, Olofsson J, Jonsson R, Davidsson A. High Prevalence of Influenza Specific Antibody Secreting Cells in Nasal Mucosa. Scandinavian Journal of Immunology 2001;54(1-2):243-247.
26. Clements ML, Murphy BR. Development and persistence of local and systemic antibody responses in adults given live attenuated or inactivated influenza A virus vaccine. J Clin Microbiol 1986;23(1):66-72.
27. Belshe RB, Gruber WC, Mendelman PM et al. Correlates of immune protection induced by live, attenuated, cold-adapted, trivalent, intranasal influenza virus vaccine. J Infect Dis 2000;181(3):1133-1137.
28. Muszkat M, Yehuda AB, Schein MH et al. Local and systemic immune response in community-dwelling elderly after intranasal or intramuscular immunization with inactivated influenza vaccine. J Med Virol 2000;61(1):100-106.
29. Muszkat M, Friedman G, Schein MH et al. Local SIgA response following administration of a novel intranasal inactivated influenza virus vaccine in community residing elderly. Vaccine 2000;18(16):1696-1699.
30. World Health Organization. Laboratory procedures: identification of influenza isolates by hemagglutination inhibition. http://www who int/emc-documents/influenza/docs/animalinfluenza/HTML/lab_procedures_e htm, 2002.
31. Cox RJ, Brokstad KA, Zuckerman MA, Wood JM, Haaheim LR, Oxford JS. An early humoral immune response in peripheral blood following parenteral inactivated influenza vaccination. Vaccine 1994;12(11):993-999.
32. Brokstad KA, Cox RJ, Major D, Wood JM, Haaheim LR. Cross-reaction but no avidity change of the serum antibody response after influenza vaccination. Vaccine 1995;13(16):1522-1528.
33. el Madhun AS, Cox RJ, Soreide A, Olofsson J, Haaheim LR. Systemic and mucosal immune responses in young children and adults after parenteral influenza vaccination. J Infect Dis 1998;178(4):933-939.

34. Treanor JJ, Kotloff K, Betts RF et al. Evaluation of trivalent, live, cold-adapted (CAIV-T) and inactivated (TIV) influenza vaccines in prevention of virus infection and illness following challenge of adults with wild-type influenza A (H1N1), A (H3N2), and B viruses. Vaccine 1999;18(9-10):899-906.
35. Abramson JS. Intranasal, cold-adapted, live, attenuated influenza vaccine. Pediatr Infect Dis J 1999;18(12):1103-1104.
36. Cox RJ, Brokstad KA, Ogra P. Influenza virus: immunity and vaccination strategies. Comparison of the immune response to inactivated and live, attenuated influenza vaccines. Scand J Immunol 2004;59(1):1-15.
37. McMichael AJ, Gotch FM, Noble GR, Beare PA. Cytotoxic T-cell immunity to influenza. New England Journal of Medicine 309(1):13-7, 1983.
38. Treanor J. Influenza. In: Mandell G, Barrett J, Dolin R, editors. Mandell: Principles and Practice of Infectious Diseases. 5th ed. Philadelphia: Churchill Livingstone, Inc.; 2000:1823-1842.
39. Bot A, Bot S, Bona CA. Protective role of gamma interferon during the recall response to influenza virus. J Virol 1998;72(8):6637-6645.
40. Deng Y, Jing Y, Campbell AE, Gravenstein S. Age-related impaired type 1 T cell responses to influenza: reduced activation ex vivo, decreased expansion in CTL culture in vitro, and blunted response to influenza vaccination in vivo in the elderly. J Immunol 2004;172(6):3437-3446.
41. Guthrie T, Hobbs CG, Davenport V, Horton RE, Heyderman RS, Williams NA. Parenteral influenza vaccination influences mucosal and systemic T cell-mediated immunity in healthy adults. J Infect Dis 2004;190(11):1927-1935.
42. Gorse GJ, Belshe RB. Enhancement of anti-influenza A virus cytotoxicity following influenza A virus vaccination in older, chronically ill adults. J Clin Microbiol 1990;28(11):2539-2550.
43. Cooper CL, Davis HL, Morris ML et al. Safety and immunogenicity of CPG 7909 injection as an adjuvant to Fluarix influenza vaccine. Vaccine 2004;22(23-24):3136-3143.
44. McElhaney JE, Beattie BL, Devine R, Grynoch R, Toth EL, Bleackley RC. Age-related decline in interleukin 2 production in response to influenza vaccine. J Am Geriatr Soc 1990;38(6):652-658.
45. Frasca D, Riley RL, Blomberg BB. Humoral immune response and B-cell functions including immunoglobulin class switch are downregulated in aged mice and humans. Semin Immunol 2005;17(5):378-384.
46. Naylor K, Li G, Vallejo AN et al. The influence of age on T cell generation and TCR diversity. J Immunol 2005;174(11):7446-7452.
47. Gardner EM, Gonzalez EW, Nogusa S, Murasko DM. Age-related changes in the immune response to influenza vaccination in a racially diverse, healthy elderly population. Vaccine 2006;24(10):1609-1614.
48. McElhaney JE, Ewen C, Zhou X et al. Granzyme B: Correlates with protection and enhanced CTL response to influenza vaccination in older adults. Vaccine 2009;27(18):2418-2425.
49. Boyce TG, Poland GA. Promises and challenges of live-attenuated intranasal influenza vaccines across the age spectrum: a review. Biomed Pharmacother 2000;54(4):210-218.
50. Murasko DM, Bernstein ED, Gardner EM et al. Role of humoral and cell-mediated immunity in protection from influenza disease after immunization of healthy elderly. Exp Gerontol 2002;37(2-3):427-439.
51. Jackson LA, Holmes SJ, Mendelman PM, Huggins L, Cho I, Rhorer J. Safety of a trivalent live attenuated intranasal influenza vaccine, FluMist(TM), administered in addition to

parenteral trivalent inactivated influenza vaccine to seniors with chronic medical conditions. Vaccine 1999;17(15-16):1905-1909.
52. Sasaki S, He XS, Holmes TH et al. Influence of prior influenza vaccination on antibody and B-cell responses. PLoS One 2008;3(8):e2975.
53. Cooper CL, Davis H, Cameron DW. Influenza vaccination with 1/10th the full dose. N Engl J Med 2004;351(22):2339-2340.
54. Kenney RT, Frech SA, Muenz LR, Villar CP, Glenn GM. Dose sparing with intradermal injection of influenza vaccine. N Engl J Med 2004;351(22):2295-2301.
55. Belshe RB, Newman FK, Cannon J et al. Serum antibody responses after intradermal vaccination against influenza. N Engl J Med 2004;351(22):2286-2294.
56. Muszkat M, Greenbaum E, Ben-Yehuda A et al. Local and systemic immune response in nursing-home elderly following intranasal or intramuscular immunization with inactivated influenza vaccine. Vaccine 2003;21(11-12):1180-1186.
57. Falsey AR, Treanor JJ, Tornieporth N, Capellan J, Gorse GJ. Randomized, double-blind controlled phase 3 trial comparing the immunogenicity of high-dose and standard-dose influenza vaccine in adults 65 years of age and older. J Infect Dis 2009;200(2):172-180.
58. Ben Yehuda A, Joseph A, Barenholz Y et al. Immunogenicity and safety of a novel IL-2-supplemented liposomal influenza vaccine (INFLUSOME-VAC) in nursing-home residents. Vaccine 2003;21(23):3169-3178.
59. Frech SA, Kenney RT, Spyr CA et al. Improved immune responses to influenza vaccination in the elderly using an immunostimulant patch. Vaccine 2005;23(7):946-950.
60. Tumpey TM, Renshaw M, Clements JD, Katz JM. Mucosal delivery of inactivated influenza vaccine induces B-cell-dependent heterosubtypic cross-protection against lethal influenza A H5N1 virus infection. J Virol 2001;75(11):5141-5150.
61. Gravenstein S, Drinka P, Duthie EH et al. Efficacy of an influenza hemagglutinin-diphtheria toxoid conjugate vaccine in elderly nursing home subjects during an influenza outbreak. J Am Geriatr Soc 1994;42(3):245-251.
62. Podda A. The adjuvanted influenza vaccines with novel adjuvants: experience with the MF59-adjuvanted vaccine. Vaccine 2001;19(17-19):2673-2680.
63. Frey S, Poland G, Percell S, Podda A. Comparison of the safety, tolerability, and immunogenicity of a MF59-adjuvanted influenza vaccine and a non-adjuvanted influenza vaccine in non-elderly adults. Vaccine 2003;21(27-30):4234-4237.
64. Boyce TG, Hsu HH, Sannella EC et al. Safety and immunogenicity of adjuvanted and unadjuvanted subunit influenza vaccines administered intranasally to healthy adults. Vaccine 2000;19(2-3):217-226.
65. Banzhoff A, Nacci P, Podda A. A new MF59-adjuvanted influenza vaccine enhances the immune response in the elderly with chronic diseases: results from an immunogenicity meta-analysis. Gerontology 2003;49(3):177-184.
66. Sindoni D, La F, V, Squeri R et al. Comparison between a conventional subunit vaccine and the MF59-adjuvanted subunit influenza vaccine in the elderly: an evaluation of the safety, tolerability and immunogenicity. J Prev Med Hyg 2009;50(2):121-126.
67. Stephenson I, Bugarini R, Nicholson KG et al. Cross-reactivity to highly pathogenic avian influenza H5N1 viruses after vaccination with nonadjuvanted and MF59-adjuvanted influenza A/Duck/Singapore/97 (H5N3) vaccine: a potential priming strategy. J Infect Dis 2005;191(8):1210-1215.
68. varez-Olmos MI, Oberhelman RA. Probiotic agents and infectious diseases: a modern perspective on a traditional therapy. Clin Infect Dis 2001;32(11):1567-1576.
69. Elmer GW. Probiotics: "living drugs". Am J Health Syst Pharm 2001;58(12):1101-1109.

70. Bruce AW, Reid G. Intravaginal instillation of lactobacilli for prevention of recurrent urinary tract infections. Can J Microbiol 1988;34(3):339-343.
71. Hilton E, Rindos P, Isenberg HD. Lactobacillus GG vaginal suppositories and vaginitis. J Clin Microbiol 1995;33(5):1433.
72. Reid G, Bruce AW, Taylor M. Influence of three-day antimicrobial therapy and lactobacillus vaginal suppositories on recurrence of urinary tract infections. Clin Ther 1992;14(1):11-16.
73. Hudault S, Lievin V, Bernet-Camard MF, Servin AL. Antagonistic activity exerted in vitro and in vivo by Lactobacillus casei (strain GG) against Salmonella typhimurium C5 infection. Appl Environ Microbiol 1997;63(2):513-518.
74. Naaber P, Mikelsaar RH, Salminen S, Mikelsaar M. Bacterial translocation, intestinal microflora and morphological changes of intestinal mucosa in experimental models of Clostridium difficile infection. J Med Microbiol 1998;47(7):591-598.
75. Chan RC, Reid G, Irvin RT, Bruce AW, Costerton JW. Competitive exclusion of uropathogens from human uroepithelial cells by Lactobacillus whole cells and cell wall fragments. Infect Immun 1985;47(1):84-89.
76. Salminen S, Isolauri E, Salminen E. Clinical uses of probiotics for stabilizing the gut mucosal barrier: successful strains and future challenges. Antonie Van Leeuwenhoek 1996;70(2-4):347-358.
77. Isolauri E, Majamaa H, Arvola T, Rantala I, Virtanen E, Arvilommi H. Lactobacillus casei strain GG reverses increased intestinal permeability induced by cow milk in suckling rats. Gastroenterology 1993;105(6):1643-1650.
78. Dong MY, Chang TW, Gorbach SL. Effects of feeding lactobacillus GG on lethal irradiation in mice. Diagn Microbiol Infect Dis 1987;7(1):1-7.
79. Lee DJ, Drongowski RA, Coran AG, Harmon CM. Evaluation of probiotic treatment in a neonatal animal model. Pediatr Surg Int 2000;16(4):237-242.
80. Parent D, Bossens M, Bayot D et al. Therapy of bacterial vaginosis using exogenously-applied Lactobacilli acidophili and a low dose of estriol: a placebo-controlled multicentric clinical trial. Arzneimittelforschung 1996;46(1):68-73.
81. Mattar AF, Teitelbaum DH, Drongowski RA, Yongyi F, Harmon CM, Coran AG. Probiotics up-regulate MUC-2 mucin gene expression in a Caco-2 cell-culture model. Pediatr Surg Int 2002;18(7):586-590.
82. Mattar AF, Drongowski RA, Coran AG, Harmon CM. Effect of probiotics on enterocyte bacterial translocation in vitro. Pediatr Surg Int 2001;17(4):265-268.
83. Banasaz M, Norin E, Holma R, Midtvedt T. Increased enterocyte production in gnotobiotic rats mono-associated with Lactobacillus rhamnosus GG. Appl Environ Microbiol 2002;68(6):3031-3034.
84. Yan F, Polk DB. Probiotic bacterium prevents cytokine-induced apoptosis in intestinal epithelial cells. J Biol Chem 2002;277(52):50959-50965.
85. Miettinen M, Matikainen S, Vuopio-Varkila J et al. Lactobacilli and streptococci induce interleukin-12 (IL-12), IL-18, and gamma interferon production in human peripheral blood mononuclear cells. Infect Immun 1998;66(12):6058-6062.
86. Miettinen M, Lehtonen A, Julkunen I, Matikainen S. Lactobacilli and Streptococci activate NF-kappa B and STAT signaling pathways in human macrophages. J Immunol 2000;164(7):3733-3740.
87. Veckman V, Miettinen M, Pirhonen J, Siren J, Matikainen S, Julkunen I. Streptococcus pyogenes and Lactobacillus rhamnosus differentially induce maturation and production of Th1-type cytokines and chemokines in human monocyte-derived dendritic cells. J Leukoc Biol 2004;75(5):764-771.

88. Braat H, De Jong EC, van den Brande JM et al. Dichotomy between Lactobacillus rhamnosus and Klebsiella pneumoniae on dendritic cell phenotype and function. J Mol Med 2004;82(3):197-205.
89. Korhonen R, Korpela R, Saxelin M, Maki M, Kankaanranta H, Moilanen E. Induction of nitric oxide synthesis by probiotic Lactobacillus rhamnosus GG in J774 macrophages and human T84 intestinal epithelial cells. Inflammation 2001;25(4):223-232.
90. Isolauri E, Joensuu J, Suomalainen H, Luomala M, Vesikari T. Improved immunogenicity of oral D x RRV reassortant rotavirus vaccine by Lactobacillus casei GG. Vaccine 1995;13(3):310-312.
91. Fang H, Elina T, Heikki A, Seppo S. Modulation of humoral immune response through probiotic intake. FEMS Immunol Med Microbiol 2000;29(1):47-52.
92. Pochard P, Gosset P, Grangette C et al. Lactic acid bacteria inhibit TH2 cytokine production by mononuclear cells from allergic patients. J Allergy Clin Immunol 2002;110(4):617-623.
93. Ocana VS, De Ruiz Holgado AA, Nader-Macias ME. Growth inhibition of Staphylococcus aureus by H2O2-producing Lactobacillus paracasei subsp. paracasei isolated from the human vagina. FEMS Immunol Med Microbiol 1999;23(2):87-92.
94. Fang W, Shi M, Huang L, Chen J, Wang Y. Antagonism of lactic acid bacteria towards Staphylococcus aureus and Escherichia coli on agar plates and in milk. Vet Res 1996;27(1):3-12.
95. Forestier C, De CC, Vatoux C, Joly B. Probiotic activities of Lactobacillus casei rhamnosus: in vitro adherence to intestinal cells and antimicrobial properties. Res Microbiol 2001;152(2):167-173.
96. Lehto EM, Salminen SJ. Inhibition of Salmonella typhimurium adhesion to Caco-2 cell cultures by Lactobacillus strain GG spent culture supernate: only a pH effect? FEMS Immunol Med Microbiol 1997;18(2):125-132.
97. Gan BS, Kim J, Reid G, Cadieux P, Howard JC. Lactobacillus fermentum RC-14 inhibits Staphylococcus aureus infection of surgical implants in rats. J Infect Dis 2002;185(9):1369-1372.
98. Reid G, Lam D, Bruce AW, van der Mei HC, Busscher HJ. Adhesion of lactobacilli to urinary catheters and diapers: effect of surface properties. J Biomed Mater Res 1994;28(6):731-734.
99. Reid G, Tieszer C, Lam D. Influence of lactobacilli on the adhesion of Staphylococcus aureus and Candida albicans to fibers and epithelial cells. J Ind Microbiol 1995;15(3):248-253.
100. Reid G. Adhesion of urogenital organisms to polymers and prosthetic devices. Methods Enzymol 1995;253:514-519.
101. Velraeds MM, van der Mei HC, Reid G, Busscher HJ. Inhibition of initial adhesion of uropathogenic Enterococcus faecalis by biosurfactants from Lactobacillus isolates. Appl Environ Microbiol 1996;62(6):1958-1963.
102. Silva M, Jacobus NV, Deneke C, Gorbach SL. Antimicrobial substance from a human Lactobacillus strain. Antimicrob Agents Chemother 1987;31(8):1231-1233.
103. Goldin BR, Gorbach SL, Saxelin M, Barakat S, Gualtieri L, Salminen S. Survival of Lactobacillus species (strain GG) in human gastrointestinal tract. Dig Dis Sci 1992;37(1):121-128.
104. Elo S, Saxelin M, Salminen S. Attachment of lactobacillus casei strain GG to human colon carcinoma cell line Caco-2: comparison with other dairy strains. Letters in Applied Microbiology 1991;13:154-156.

105. Alander M, Korpela R, Saxelin M, Vilpponen-Salmela T, Mattila-Sandholm T, von Wright A. Recovery of Lactobacillus rhamnosus GG from human colonic biopsies. Lett Appl Microbiol 1997;24(5):361-364.
106. Donahue DC, Deighton M, Ahokas JT, Salminen S. Toxicity of lactic acid bacteria. In: Salminen S, Wright A, editors. Lactic Acid Bacteria. New York: Marcel Dekker Inc.; 1993:307-313.
107. Salminen SJ, Donahue DC. Safety assessment of Lactobacillus strain GG (ATCC 53103). Nutrition Today 1996;Supplement 31(6):12S-15S.
108. Berg RD, Wommack E, Deitch EA. Immunosuppression and intestinal bacterial overgrowth synergistically promote bacterial translocation. Arch Surg 1988;123(11):1359-1364.
109. Deitch EA, Maejima K, Berg R. Effect of oral antibiotics and bacterial overgrowth on the translocation of the GI tract microflora in burned rats. J Trauma 1985;25(5):385-392.
110. Reddy BS, MacFie J, Gatt M, farlane-Smith L, Bitzopoulou K, Snelling AM. Commensal bacteria do translocate across the intestinal barrier in surgical patients. Clin Nutr 2007;26(2):208-215.
111. Ouwehand AC, Salminen S, Roberts PJ, Ovaska J, Salminen E. Disease-dependent adhesion of lactic acid bacteria to the human intestinal mucosa. Clin Diagn Lab Immunol 2003;10(4):643-646.
112. Vesterlund S, Vankerckhoven V, Saxelin M, Goossens H, Salminen S, Ouwehand AC. Safety assessment of Lactobacillus strains: presence of putative risk factors in faecal, blood and probiotic isolates. Int J Food Microbiol 2007;116(3):325-331.
113. Ruseler-van Embden JG, van Lieshout LM, Gosselink MJ, Marteau P. Inability of Lactobacillus casei strain GG, L. acidophilus, and Bifidobacterium bifidum to degrade intestinal mucus glycoproteins. Scand J Gastroenterol 1995;30(7):675-680.
114. Douglas CW, Brown PR, Preston FE. Platelet aggregation by oral streptococci. FEMS Microbiol Lett 1990;60(1-2):63-67.
115. Harty DW, Patrikakis M, Hume EB, Oakey HJ, Knox KW. The aggregation of human platelets by Lactobacillus species. J Gen Microbiol 1993;139(12):2945-2951.
116. Harty DW, Oakey HJ, Patrikakis M, Hume EB, Knox KW. Pathogenic potential of lactobacilli. Int J Food Microbiol 1994;24(1-2):179-189.
117. Mathur S, Singh R. Antibiotic resistance in food lactic acid bacteria--a review. Int J Food Microbiol 2005;105(3):281-295.
118. Salyers AA, Gupta A, Wang Y. Human intestinal bacteria as reservoirs for antibiotic resistance genes. Trends Microbiol 2004;12(9):412-416.
119. Ammor MS, Florez AB, Mayo B. Antibiotic resistance in non-enterococcal lactic acid bacteria and bifidobacteria. Food Microbiol 2007;24(6):559-570.
120. Borriello SP, Hammes WP, Holzapfel W et al. Safety of probiotics that contain lactobacilli or bifidobacteria. Clin Infect Dis 2003;36(6):775-780.
121. Danielsen M, Wind A. Susceptibility of Lactobacillus spp. to antimicrobial agents. Int J Food Microbiol 2003;82(1):1-11.
122. Perreten V, Schwarz F, Cresta L, Boeglin M, Dasen G, Teuber M. Antibiotic resistance spread in food. Nature 1997;389(6653):801-802.
123. Ammor MS, Florez AB, van Hoek AH et al. Molecular characterization of intrinsic and acquired antibiotic resistance in lactic acid bacteria and bifidobacteria. J Mol Microbiol Biotechnol 2008;14(1-3):6-15.
124. Klein G, Hallmann C, Casas IA, Abad J, Louwers J, Reuter G. Exclusion of vanA, vanB and vanC type glycopeptide resistance in strains of Lactobacillus reuteri and Lactobacillus

rhamnosus used as probiotics by polymerase chain reaction and hybridization methods. J Appl Microbiol 2000;89(5):815-824.
125. Tynkkynen S, Singh KV, Varmanen P. Vancomycin resistance factor of Lactobacillus rhamnosus GG in relation to enterococcal vancomycin resistance (van) genes. Int J Food Microbiol 1998;41(3):195-204.
126. Mater DD, Langella P, Corthier G, Flores MJ. Evidence of vancomycin resistance gene transfer between enterococci of human origin in the gut of mice harbouring human microbiota. J Antimicrob Chemother 2005;56(5):975-978.
127. Mater DD, Langella P, Corthier G, Flores MJ. A probiotic Lactobacillus strain can acquire vancomycin resistance during digestive transit in mice. J Mol Microbiol Biotechnol 2008;14(1-3):123-127.
128. Bernardeau M, Guguen M, Vernoux JP. Beneficial lactobacilli in food and feed: long-term use, biodiversity and proposals for specific and realistic safety assessments. FEMS Microbiol Rev 2006;30(4):487-513.
129. Cheah PY. Hypotheses for the etiology of colorectal cancer--an overview. Nutr Cancer 1990;14(1):5-13.
130. Allen SJ, Okoko B, Martinez E, Gregorio G, Dans LF. Probiotics for treating infectious diarrhoea. Cochrane Database Syst Rev 2004;(2):CD003048.
131. Saxelin M, Pessi T, Salminen S. Fecal recovery following oral administration of Lactobacillus strain GG (ATCC 53103) in gelatine capsules to healthy volunteers. Int J Food Microbiol 1995;25(2):199-203.
132. Alander M, Satokari R, Korpela R et al. Persistence of colonization of human colonic mucosa by a probiotic strain, Lactobacillus rhamnosus GG, after oral consumption. Appl Environ Microbiol 1999;65(1):351-354.
133. Schultz M, Linde HJ, Lehn N et al. Immunomodulatory consequences of oral administration of Lactobacillus rhamnosus strain GG in healthy volunteers. J Dairy Res 2003;70(2):165-173.
134. Siitonen S, Vapaatalo H, Salminen S et al. Effect of Lactobacillus GG yoghurt in prevention of antibiotic associated diarrhoea. Ann Med 1990;22(1):57-59.
135. Oksanen PJ, Salminen S, Saxelin M et al. Prevention of travellers' diarrhoea by Lactobacillus GG. Ann Med 1990;22(1):53-56.
136. Ling WH, Korpela R, Mykkanen H, Salminen S, Hanninen O. Lactobacillus strain GG supplementation decreases colonic hydrolytic and reductive enzyme activities in healthy female adults. J Nutr 1994;124(1):18-23.
137. Benno Y, He F, Hosoda M et al. Effects of lactobacillus GG yogurt on human intestinal microecology in Japanese subjects. Nutrition Today 1996;31:9S-11S.
138. Hilton E, Kolakowski P, Singer C, Smith M. Efficacy of Lactobacillus GG as a Diarrheal Preventive in Travelers. J Travel Med 1997;4(1):41-43.
139. Pelto L, Isolauri E, Lilius EM, Nuutila J, Salminen S. Probiotic bacteria down-regulate the milk-induced inflammatory response in milk-hypersensitive subjects but have an immunostimulatory effect in healthy subjects. Clin Exp Allergy 1998;28(12):1474-1479.
140. Gotteland M, Cruchet S, Verbeke S. Effect of Lactobacillus ingestion on the gastrointestinal mucosal barrier alterations induced by indometacin in humans. Aliment Pharmacol Ther 2001;15(1):11-17.
141. Ahola AJ, Yli-Knuuttila H, Suomalainen T et al. Short-term consumption of probiotic-containing cheese and its effect on dental caries risk factors. Arch Oral Biol 2002;47(11):799-804.

142. Gluck U, Gebbers JO. Ingested probiotics reduce nasal colonization with pathogenic bacteria (Staphylococcus aureus, Streptococcus pneumoniae, and beta-hemolytic streptococci). Am J Clin Nutr 2003;77(2):517-520.
143. Cohen LA, Crespin JS, Wolper C et al. Soy isoflavone intake and estrogen excretion patterns in young women: effect of probiotic administration. In Vivo 2007;21(3):507-512.
144. Kekkonen RA, Vasankari TJ, Vuorimaa T, Haahtela T, Julkunen I, Korpela R. The effect of probiotics on respiratory infections and gastrointestinal symptoms during training in marathon runners. Int J Sport Nutr Exerc Metab 2007;17(4):352-363.
145. Moreira A, Kekkonen R, Korpela R, Delgado L, Haahtela T. Allergy in marathon runners and effect of Lactobacillus GG supplementation on allergic inflammatory markers. Respir Med 2007;101(6):1123-1131.
146. Kekkonen RA, Lummela N, Karjalainen H et al. Probiotic intervention has strain-specific anti-inflammatory effects in healthy adults. World J Gastroenterol 2008;14(13):2029-2036.
147. Kekkonen RA, Sysi-Aho M, Seppanen-Laakso T et al. Effect of probiotic Lactobacillus rhamnosus GG intervention on global serum lipidomic profiles in healthy adults. World J Gastroenterol 2008;14(20):3188-3194.
148. Sepp E, Mikelsaar M, Salminen S. Effect of administration of Lactobacillus caseii strain GG on the gastrointestinal microbiota of newborns. Microbial Ecology in Health and Disease 1993;6(6):309-314.
149. Sheen P, Oberhelman RA, Gilman RH, Cabrera L, Verastegui M, Madico G. Short report: a placebo-controlled study of Lactobacillus GG colonization in one-to-three-year-old Peruvian children. Am J Trop Med Hyg 1995;52(5):389-392.
150. Agarwal R, Sharma N, Chaudhry R et al. Effects of oral Lactobacillus GG on enteric microflora in low-birth-weight neonates. J Pediatr Gastroenterol Nutr 2003;36(3):397-402.
151. Petschow BW, Figueroa R, Harris CL, Beck LB, Ziegler E, Goldin B. Effects of feeding an infant formula containing Lactobacillus GG on the colonization of the intestine: a dose-response study in healthy infants. J Clin Gastroenterol 2005;39(9):786-790.
152. Hatakka K, Savilahti E, Ponka A et al. Effect of long term consumption of probiotic milk on infections in children attending day care centres: double blind, randomised trial. BMJ 2001;322(7298):1327.
153. Rautava S, Arvilommi H, Isolauri E. Specific probiotics in enhancing maturation of IgA responses in formula-fed infants. Pediatr Res 2006;60(2):221-224.
154. Vendt N, Grunberg H, Tuure T et al. Growth during the first 6 months of life in infants using formula enriched with Lactobacillus rhamnosus GG: double-blind, randomized trial. J Hum Nutr Diet 2006;19(1):51-58.
155. Smerud HK, Kleiveland CR, Mosland AR, Grave G, Birkeland S-E. Effect of a probiotic milk product on gastrointestinal and respiratory infections in children attending day-care. Microbial Ecology in Health and Disease 2008;1-6.
156. Kukkonen K, Savilahti E, Haahtela T et al. Long-term safety and impact on infection rates of postnatal probiotic and prebiotic (synbiotic) treatment: randomized, double-blind, placebo-controlled trial. Pediatrics 2008;122(1):8-12.
157. Schultz M, Gottl C, Young RJ, Iwen P, Vanderhoof JA. Administration of oral probiotic bacteria to pregnant women causes temporary infantile colonization. J Pediatr Gastroenterol Nutr 2004;38(3):293-297.
158. Kalliomaki M, Salminen S, Poussa T, Isolauri E. Probiotics during the first 7 years of life: a cumulative risk reduction of eczema in a randomized, placebo-controlled trial. J Allergy Clin Immunol 2007;119(4):1019-1021.

159. Kalliomaki M, Salminen S, Arvilommi H, Kero P, Koskinen P, Isolauri E. Probiotics in primary prevention of atopic disease: a randomised placebo-controlled trial. Lancet 2001;357(9262):1076-1079.
160. Rautava S, Kalliomaki M, Isolauri E. Probiotics during pregnancy and breast-feeding might confer immunomodulatory protection against atopic disease in the infant. J Allergy Clin Immunol 2002;109(1):119-121.
161. Kalliomaki M, Salminen S, Poussa T, Arvilommi H, Isolauri E. Probiotics and prevention of atopic disease: 4-year follow-up of a randomised placebo-controlled trial. Lancet 2003;361(9372):1869-1871.
162. Laitinen K, Kalliomaki M, Poussa T, Lagstrom H, Isolauri E. Evaluation of diet and growth in children with and without atopic eczema: follow-up study from birth to 4 years. Br J Nutr 2005;94(4):565-574.
163. Gueimonde M, Sakata S, Kalliomaki M, Isolauri E, Benno Y, Salminen S. Effect of maternal consumption of lactobacillus GG on transfer and establishment of fecal bifidobacterial microbiota in neonates. J Pediatr Gastroenterol Nutr 2006;42(2):166-170.
164. Rinne M, Kalliomaki M, Salminen S, Isolauri E. Probiotic intervention in the first months of life: short-term effects on gastrointestinal symptoms and long-term effects on gut microbiota. J Pediatr Gastroenterol Nutr 2006;43(2):200-205.
165. Kaplas N, Isolauri E, Lampi AM, Ojala T, Laitinen K. Dietary counseling and probiotic supplementation during pregnancy modify placental phospholipid fatty acids. Lipids 2007;42(9):865-870.
166. Kukkonen K, Savilahti E, Haahtela T et al. Probiotics and prebiotic galacto-oligosaccharides in the prevention of allergic diseases: a randomized, double-blind, placebo-controlled trial. J Allergy Clin Immunol 2007;119(1):192-198.
167. Kopp MV, Hennemuth I, Heinzmann A, Urbanek R. Randomized, double-blind, placebo-controlled trial of probiotics for primary prevention: no clinical effects of Lactobacillus GG supplementation. Pediatrics 2008;121(4):e850-e856.
168. Kopp MV, Goldstein M, Dietschek A, Sofke J, Heinzmann A, Urbanek R. Lactobacillus GG has in vitro effects on enhanced interleukin-10 and interferon-gamma release of mononuclear cells but no in vivo effects in supplemented mothers and their neonates. Clin Exp Allergy 2008;38(4):602-610.
169. Rautio M, Jousimies-Somer H, Kauma H et al. Liver abscess due to a Lactobacillus rhamnosus strain indistinguishable from L. rhamnosus strain GG. Clin Infect Dis 1999;28(5):1159-1160.
170. Mackay AD, Taylor MB, Kibbler CC, Hamilton-Miller JM. Lactobacillus endocarditis caused by a probiotic organism. Clin Microbiol Infect 1999;5(5):290-292.
171. Kunz AN, Noel JM, Fairchok MP. Two cases of Lactobacillus bacteremia during probiotic treatment of short gut syndrome. J Pediatr Gastroenterol Nutr 2004;38(4):457-458.
172. Land MH, Rouster-Stevens K, Woods CR, Cannon ML, Cnota J, Shetty AK. Lactobacillus sepsis associated with probiotic therapy. Pediatrics 2005;115(1):178-181.
173. De Groote MA, Frank DN, Dowell E, Glode MP, Pace NR. Lactobacillus rhamnosus GG bacteremia associated with probiotic use in a child with short gut syndrome. Pediatr Infect Dis J 2005;24(3):278-280.
174. Boyd MA, Antonio MA, Hillier SL. Comparison of API 50 CH strips to whole-chromosomal DNA probes for identification of Lactobacillus species. J Clin Microbiol 2005;43(10):5309-5311.

175. Salminen MK, Tynkkynen S, Rautelin H et al. Lactobacillus bacteremia during a rapid increase in probiotic use of Lactobacillus rhamnosus GG in Finland. Clin Infect Dis 2002;35(10):1155-1160.
176. Salminen MK, Rautelin H, Tynkkynen S et al. Lactobacillus bacteremia, clinical significance, and patient outcome, with special focus on probiotic L. rhamnosus GG. Clin Infect Dis 2004;38(1):62-69.
177. Ouwehand AC, Saxelin M, Salminen S. Phenotypic differences between commercial Lactobacillus rhamnosus GG and L. rhamnosus strains recovered from blood. Clin Infect Dis 2004;39(12):1858-1860.
178. Fukushima Y, Kawata Y, Hara H, Terada A, Mitsuoka T. Effect of a probiotic formula on intestinal immunoglobulin A production in healthy children. Int J Food Microbiol 1998;42(1-2):39-44.
179. Link-Amster H, Rochat F, Saudan KY, Mignot O, Aeschlimann JM. Modulation of a specific humoral immune response and changes in intestinal flora mediated through fermented milk intake. FEMS Immunol Med Microbiol 1994;10(1):55-63.
180. Kaila M, Isolauri E, Soppi E, Virtanen E, Laine S, Arvilommi H. Enhancement of the circulating antibody secreting cell response in human diarrhea by a human Lactobacillus strain. Pediatr Res 1992;32(2):141-144.
181. Majamaa H, Isolauri E, Saxelin M, Vesikari T. Lactic acid bacteria in the treatment of acute rotavirus gastroenteritis. J Pediatr Gastroenterol Nutr 1995;20(3):333-338.
182. Kaila M, Isolauri E, Saxelin M, Arvilommi H, Vesikari T. Viable versus inactivated lactobacillus strain GG in acute rotavirus diarrhoea. Arch Dis Child 1995;72(1):51-53.
183. Cangemi de GR, Santos V, Nader-Macias ME. Protective effect of intranasally inoculated Lactobacillus fermentum against Streptococcus pneumoniae challenge on the mouse respiratory tract. FEMS Immunol Med Microbiol 2001;31(3):187-195.
184. Alvarez S, Herrero C, Bru E, Perdigon G. Effect of Lactobacillus casei and yogurt administration on prevention of Pseudomonas aeruginosa infection in young mice. J Food Prot 2001;64(11):1768-1774.
185. Hori T, Kiyoshima J, Shida K, Yasui H. Effect of intranasal administration of Lactobacillus casei Shirota on influenza virus infection of upper respiratory tract in mice. Clin Diagn Lab Immunol 2001;8(3):593-597.
186. Hori T, Kiyoshima J, Shida K, Yasui H. Augmentation of cellular immunity and reduction of influenza virus titer in aged mice fed Lactobacillus casei strain Shirota. Clin Diagn Lab Immunol 2002;9(1):105-108.
187. Yasui H, Kiyoshima J, Hori T. Reduction of influenza virus titer and protection against influenza virus infection in infant mice fed Lactobacillus casei Shirota. Clin Diagn Lab Immunol 2004;11(4):675-679.
188. Madsen K, Cornish A, Soper P et al. Probiotic bacteria enhance murine and human intestinal epithelial barrier function. Gastroenterology 2001;121(3):580-591.
189. Otte JM, Podolsky DK. Functional modulation of enterocytes by gram-positive and gram-negative microorganisms. Am J Physiol Gastrointest Liver Physiol 2004;286(4):G613-G626.
190. Christensen HR, Frokiaer H, Pestka JJ. Lactobacilli differentially modulate expression of cytokines and maturation surface markers in murine dendritic cells. J Immunol 2002;168(1):171-178.
191. Matsuguchi T, Takagi A, Matsuzaki T et al. Lipoteichoic acids from Lactobacillus strains elicit strong tumor necrosis factor alpha-inducing activities in macrophages through Toll-like receptor 2. Clin Diagn Lab Immunol 2003;10(2):259-266.

192. Miettinen M, Vuopio-Varkila J, Varkila K. Production of human tumor necrosis factor alpha, interleukin-6, and interleukin-10 is induced by lactic acid bacteria. Infect Immun 1996;64(12):5403-5405.
193. Bunout D, Hirsch S, Pia de la MM et al. Effects of prebiotics on the immune response to vaccination in the elderly. JPEN J Parenter Enteral Nutr 2002;26(6):372-376.
194. Maassen CB, van Holten-Neelen C, Balk F et al. Strain-dependent induction of cytokine profiles in the gut by orally administered Lactobacillus strains. Vaccine 2000;18(23):2613-2623.
195. Rachmilewitz D, Katakura K, Karmeli F et al. Toll-like receptor 9 signaling mediates the anti-inflammatory effects of probiotics in murine experimental colitis. Gastroenterology 2004;126(2):520-528.
196. Sheih YH, Chiang BL, Wang LH, Liao CK, Gill HS. Systemic immunity-enhancing effects in healthy subjects following dietary consumption of the lactic acid bacterium Lactobacillus rhamnosus HN001. J Am Coll Nutr 2001;20(2 Suppl):149-156.
197. Gill HS, Rutherfurd KJ, Cross ML, Gopal PK. Enhancement of immunity in the elderly by dietary supplementation with the probiotic Bifidobacterium lactis HN019. Am J Clin Nutr 2001;74(6):833-839.
198. Chiang BL, Sheih YH, Wang LH, Liao CK, Gill HS. Enhancing immunity by dietary consumption of a probiotic lactic acid bacterium (Bifidobacterium lactis HN019): optimization and definition of cellular immune responses. Eur J Clin Nutr 2000;54(11):849-855.
199. Arunachalam K, Gill HS, Chandra RK. Enhancement of natural immune function by dietary consumption of Bifidobacterium lactis (HN019). Eur J Clin Nutr 2000;54(3):263-267.
200. Bunout D, Barrera G, Hirsch S et al. Effects of a nutritional supplement on the immune response and cytokine production in free-living Chilean elderly. JPEN J Parenter Enteral Nutr 2004;28(5):348-354.
201. Boge T, Remigy M, Vaudaine S, Tanguy J, Bourdet-Sicard R, van der WS. A probiotic fermented dairy drink improves antibody response to influenza vaccination in the elderly in two randomised controlled trials. Vaccine 2009;27(41):5677-5684.
202. Oliveres M, Diaz-Ropero M, Sierra S et al. Oral intake of Lactobacillus fermentum CECT5716 enhances the effects of influenza vaccination. Nutrition 2007;23:254-260.
203. Ohmit SE, Gross J, Victor JC, Monto AS. Reduced reaction frequencies with repeated inactivated or live-attenuated influenza vaccination. Vaccine 2009;27(7):1050-1054.
204. Weng L, Rubin EM, Bristow J. Application of sequence-based methods in human microbial ecology. Genome Res 2006;16(3):316-322.
205. Turnbaugh PJ, Ley RE, Hamady M, Fraser-Liggett CM, Knight R, Gordon JI. The human microbiome project. Nature 2007;449(7164):804-810.
206. Eckburg PB, Bik EM, Bernstein CN et al. Diversity of the human intestinal microbial flora. Science 2005;308(5728):1635-1638.
207. Backhed F, Ley RE, Sonnenburg JL, Peterson DA, Gordon JI. Host-bacterial mutualism in the human intestine. Science 2005;307(5717):1915-1920.
208. Gill SR, Pop M, Deboy RT et al. Metagenomic analysis of the human distal gut microbiome. Science 2006;312(5778):1355-1359.
209. Andoh A, Sakata S, Koizumi Y, Mitsuyama K, Fujiyama Y, Benno Y. Terminal restriction fragment length polymorphism analysis of the diversity of fecal microbiota in patients with ulcerative colitis. Inflamm Bowel Dis 2007;13(8):955-962.
210. Manichanh C, Rigottier-Gois L, Bonnaud E et al. Reduced diversity of faecal microbiota in Crohn's disease revealed by a metagenomic approach. Gut 2006;55(2):205-211.

211. Kuhbacher T, Ott SJ, Helwig U et al. Bacterial and fungal microbiota in relation to probiotic therapy (VSL#3) in pouchitis. Gut 2006;55(6):833-841.
212. Palmer C, Bik EM, DiGiulio DB, Relman DA, Brown PO. Development of the human infant intestinal microbiota. PLoS Biol 2007;5(7):e177.
213. Majamaa H, Isolauri E. Probiotics: a novel approach in the management of food allergy. Journal of Allergy & Clinical Immunology 1997;99(2):179-185.
214. Pant AR, Graham SM, Allen SJ et al. Lactobacillus GG and acute diarrhoea in young children in the tropics. J Trop Pediatr 1996;42(3):162-165.
215. Raza S, Graham SM, Allen SJ, Sultana S, Cuevas L, Hart CA. Lactobacillus GG promotes recovery from acute nonbloody diarrhea in Pakistan. Pediatric Infectious Disease Journal 1995;14(2):107-111.
216. Szajewska H, Kotowska M, Mrukowicz JZ, Armanska M, Mikolajczyk W. Efficacy of Lactobacillus GG in prevention of nosocomial diarrhea in infants. J Pediatr 2001;138(3):361-365.
217. Hooper LV, Gordon JI. Commensal host-bacterial relationships in the gut. Science 2001;292(5519):1115-1118.
218. Hooper LV, Wong MH, Thelin A, Hansson L, Falk PG, Gordon JI. Molecular analysis of commensal host-microbial relationships in the intestine. Science 2001;291(5505):881-884.
219. Aas JA, Paster BJ, Stokes LN, Olsen I, Dewhirst FE. Defining the normal bacterial flora of the oral cavity. J Clin Microbiol 2005;43(11):5721-5732.
220. Corby PM, Lyons-Weiler J, Bretz WA et al. Microbial risk indicators of early childhood caries. J Clin Microbiol 2005;43(11):5753-5759.
221. Moher D, Schulz KF, Altman DG. The CONSORT statement: revised recommendations for improving the quality of reports of parallel-group randomized trials. Ann Intern Med 2001;134(8):657-662.
222. Lane D. 16S/23S rRNA Sequencing. In: Stackerbrandt E, Goodfellow M, editors. Nucleic Acid Techniques in Bacterial Systematics. New York: Wiley and Sons; 1991:115-175.
223. Acinas SG, Sarma-Rupavtarm R, Klepac-Ceraj V, Polz MF. PCR-induced sequence artifacts and bias: insights from comparison of two 16S rRNA clone libraries constructed from the same sample. Appl Environ Microbiol 2005;71(12):8966-8969.
224. Dawson HD, Beshah E, Nishi S et al. Localized multigene expression patterns support an evolving Th1/Th2-like paradigm in response to infections with Toxoplasma gondii and Ascaris suum. Infect Immun 2005;73(2):1116-1128.
225. Latvala S, Pietila TE, Veckman V et al. Potentially probiotic bacteria induce efficient maturation but differential cytokine production in human monocyte-derived dendritic cells. World J Gastroenterol 2008;14(36):5570-5583.
226. Bayer AS, Chow AW, Ishida K, Morrison JO, Guze LB. Therapy of experimental infective endocarditis due to antibiotic-tolerant Lactobacillus plantarum-bactericidal synergy of penicillin plus gentamicin. Correlation of in vitro susceptibility studies with in vivo efficacy. Chemotherapy 1981;27(6):444-451.
227. Danielsen M, Wind A, Leisner JJ, Arpi M. Antimicrobial susceptibility of human blood culture isolates of Lactobacillus spp. Eur J Clin Microbiol Infect Dis 2007;26(4):287-289.

APPENDIX A: GUIDANCE FOR INDUSTRY

TOXICITY GRADING SCALE FOR HEALTHY ADULT AND ADOLESCENT VOLUNTEERS ENROLLED IN PREVENTIVE CLINICAL TRIALS

Adverse events in a clinical trial of an investigational vaccine must be recorded and monitored and, when appropriate, reported to FDA and others involved in an investigation (sponsors, IRBs, and investigators). (See, for example, 21 CFR 312.32, 312.33, 312.50, 312.55, 312.56, 312.60, 312.62, 312.64, 312.66). Although the use of a toxicity grading scale for adverse events would not replace these regulatory requirements, using a scale to categorize adverse events observed during a clinical trial may assist you in monitoring safety and making required reports. Nonetheless, we believe that categorization or grading of data as outlined in this document is supplementary to and should not replace full and complete data analysis.

These guidelines for toxicity grading scales are primarily intended for healthy adult and adolescent volunteers. The parameters in the tables below are not necessarily applicable to every clinical trial of healthy volunteers. The parameters monitored should be appropriate for the specific study vaccine. For some preventive vaccines under development, it may be appropriate to include additional parameters to be monitored during a clinical trial or to alter the choice of values in the toxicity table. For example, additional parameters might be added based on one or more of the following: safety signals observed in pre-clinical toxicology studies, the biological plausibility of the occurrence of certain adverse events, or previous experience with a similar licensed product.

As discussed above, the tables do not represent a recommendation to monitor all the listed parameters in all clinical trials of healthy volunteers, nor do the tables represent all possible parameters to be monitored. In addition, these tables do not represent study inclusion or exclusion criteria. We recommend that the parameters monitored be appropriate for the study vaccine administered to healthy volunteers participating in the clinical trial.

A. Tables for Clinical Abnormalities

Local Reaction to Injectable Product	Mild (Grade 1)	Moderate (Grade 2)	Severe (Grade 3)	Potentially Life Threatening (Grade 4)
Pain	Does not interfere with activity	Repeated use of non-narcotic pain reliever > 24 hours or interferes with activity	Any use of narcotic pain reliever or prevents daily activity	Emergency room (ER) visit or hospitalization
Tenderness	Mild discomfort to touch	Discomfort with movement	Significant discomfort at rest	ER visit or hospitalization

Erythema/Redness *	2.5 – 5 cm	5.1 – 10 cm	> 10 cm	Necrosis or exfoliative dermatitis
Induration/Swelling **	2.5 – 5 cm and does not interfere with activity	5.1 – 10 cm or interferes with activity	> 10 cm or prevents daily activity	Necrosis

* In addition to grading the measured local reaction at the greatest single diameter, the measurement should be recorded as a continuous variable.

** Induration/Swelling should be evaluated and graded using the functional scale as well as the actual measurement.

**Vital Signs ***	**Mild (Grade 1)**	**Moderate (Grade 2)**	**Severe (Grade 3)**	**Potentially Life Threatening (Grade 4)**
Fever (°C) **<br>(°F) **	38.0 – 38.4<br>100.4 – 101.1	38.5 – 38.9<br>101.2 – 102.0	39.0 – 40<br>102.1 – 104	> 40<br>> 104
Tachycardia - beats per minute	101 – 115	116 – 130	> 130	ER visit or hospitalization for arrhythmia
Bradycardia - beats per minute***	50 – 54	45 – 49	< 45	ER visit or hospitalization for arrhythmia
Hypertension (systolic) - mm Hg	141 – 150	151 – 155	> 155	ER visit or hospitalization for malignant hypertension
Hypertension (diastolic) - mm Hg	91 – 95	96 – 100	> 100	ER visit or hospitalization for malignant hypertension
Hypotension (systolic) – mm Hg	85 – 89	80 – 84	< 80	ER visit or hospitalization for hypotensive shock
Respiratory Rate – breaths per minute	17 – 20	21 – 25	> 25	Intubation

* Subject should be at rest for all vital sign measurements.

** Oral temperature; no recent hot or cold beverages or smoking.

*** When resting heart rate is between 60 – 100 beats per minute. Use clinical judgment when characterizing bradycardia among some healthy subject populations, for example, conditioned athletes.

Systemic (General)	Mild (Grade 1)	Moderate (Grade 2)	Severe (Grade 3)	Potentially Life Threatening (Grade 4)
Nausea/vomiting	No interference with activity or 1 – 2 episodes/24 hours	Some interference with activity or > 2 episodes/24 hours	Prevents daily activity, requires outpatient IV hydration	ER visit or hospitalization for hypotensive shock
Diarrhea	2 – 3 loose stools or < 400 gms/24 hours	4 – 5 stools or 400 – 800 gms/24 hours	6 or more watery stools or > 800gms/24 hours or requires outpatient IV hydration	ER visit or hospitalization
Headache	No interference with activity	Repeated use of non-narcotic pain reliever > 24 hours or some interference with activity	Significant; any use of narcotic pain reliever or prevents daily activity	ER visit or hospitalization
Fatigue	No interference with activity	Some interference with activity	Significant; prevents daily activity	ER visit or hospitalization
Myalgia	No interference with activity	Some interference with activity	Significant; prevents daily activity	ER visit or hospitalization

Systemic Illness	Mild (Grade 1)	Moderate (Grade 2)	Severe (Grade 3)	Potentially Life Threatening (Grade 4)
Illness or clinical adverse event (as defined according to applicable regulations)	No interference with activity	Some interference with activity not requiring medical intervention	Prevents daily activity and requires medical intervention	ER visit or hospitalization

B. Tables for Laboratory Abnormalities

The laboratory values provided in the tables below serve as guidelines and are dependent upon institutional normal parameters. Institutional normal reference ranges should be provided to demonstrate that they are appropriate.

Serum *	Mild (Grade 1)	Moderate (Grade 2)	Severe (Grade 3)	Potentially Life Threatening (Grade 4)**
Sodium – Hyponatremia mEq/L	132 – 134	130 – 131	125 – 129	< 125
Sodium – Hypernatremia mEq/L	144 – 145	146 – 147	148 – 150	> 150
Potassium – Hyperkalemia mEq/L	5.1 – 5.2	5.3 – 5.4	5.5 – 5.6	> 5.6
Potassium – Hypokalemia mEq/L	3.5 – 3.6	3.3 – 3.4	3.1 – 3.2	< 3.1
Glucose – Hypoglycemia mg/dL	65 – 69	55 – 64	45 – 54	< 45
Glucose – Hyperglycemia<br>Fasting – mg/dL<br>Random – mg/dL	 100 – 110<br>110 – 125	 111 – 125<br>126 – 200	 >125<br>>200	Insulin requirements or hyperosmolar coma
Blood Urea Nitrogen BUN mg/dL	23 – 26	27 – 31	> 31	Requires dialysis
Creatinine – mg/dL	1.5 – 1.7	1.8 – 2.0	2.1 – 2.5	> 2.5 or requires dialysis
Calcium – Hypocalcemia mg/dL	8.0 – 8.4	7.5 – 7.9	7.0 – 7.4	< 7.0
Calcium – Hypercalcemia mg/dL	10.5 – 11.0	11.1 – 11.5	11.6 – 12.0	> 12.0
Magnesium – Hypomagnesemia mg/dL	1.3 – 1.5	1.1 – 1.2	0.9 – 1.0	< 0.9
Phosphorous – Hypophosphatemia mg/dL	2.3 – 2.5	2.0 – 2.2	1.6 – 1.9	< 1.6
CPK – mg/dL	1.25 – 1.5 x ULN***	1.6 – 3.0 x ULN	3.1 –10 x ULN	> 10 x ULN
Albumin – Hypoalbuminemia g/dL	2.8 – 3.1	2.5 – 2.7	< 2.5	--
Total Protein – Hypoproteinemia g/dL	5.5 – 6.0	5.0 – 5.4	< 5.0	--
Alkaline phosphate – increase by factor	1.1 – 2.0 x ULN	2.1 – 3.0 x ULN	□3.1 – 10 x ULN	> 10 x ULN
Liver Function Tests –ALT, AST	1.1 – 2.5 x ULN	2.6 – 5.0 x ULN	5.1 – 10 x ULN	> 10 x ULN

increase by factor				
Bilirubin – when accompanied by any increase in Liver Function Test increase by factor	1.1 – 1.25 x ULN	1.26 – 1.5 x ULN	1.51 – 1.75 x ULN	> 1.75 x ULN
Bilirubin – when Liver Function Test is normal; increase by factor	1.1 – 1.5 x ULN	1.6 – 2.0 x ULN	2.0 – 3.0 x ULN	> 3.0 x ULN
Cholesterol	201 – 210	211 – 225	> 226	---
Pancreatic enzymes – amylase, lipase	1.1 – 1.5 x ULN	1.6 – 2.0 x ULN	2.1 – 5.0 x ULN	> 5.0 x ULN

* The laboratory values provided in the tables serve as guidelines and are dependent upon institutional normal parameters. Institutional normal reference ranges should be provided to demonstrate that they are appropriate.

** The clinical signs or symptoms associated with laboratory abnormalities might result in characterization of the laboratory abnormalities as Potentially Life Threatening (Grade 4). For example, a low sodium value that falls within a grade 3 parameter (125-129 mE/L) should be recorded as a grade 4 hyponatremia event if the subject had a new seizure associated with the low sodium value.

***ULN" is the upper limit of the normal range.

**Hematology ***	**Mild (Grade 1)**	**Moderate (Grade 2)**	**Severe (Grade 3)**	**Potentially Life Threatening (Grade 4)**
Hemoglobin (Female) - gm/dL	11.0 – 12.0	9.5 – 10.9	8.0 – 9.4	< 8.0
Hemoglobin (Female) change from baseline value - gm/dL	Any decrease – 1.5	1.6 – 2.0	2.1 – 5.0	> 5.0
Hemoglobin (Male) - gm/dL	12.5 – 13.5	10.5 – 12.4	8.5 – 10.4	< 8.5
Hemoglobin (Male) change from baseline value – gm/dL	Any decrease – 1.5	1.6 – 2.0	2.1 – 5.0	> 5.0
WBC Increase - cell/mm^3	10,800 – 15,000	15,001 – 20,000	20,001 – 25, 000	> 25,000
WBC Decrease - cell/mm^3	2,500 – 3,500	1,500 – 2,499	1,000 – 1,499	< 1,000
Lymphocytes Decrease - cell/mm^3	750 – 1,000	500 – 749	250 – 499	< 250
Neutrophils Decrease - cell/mm^3	1,500 – 2,000	1,000 – 1,499	500 – 999	< 500

Eosinophils - cell/mm^3	650 – 1500	1501 - 5000	> 5000	Hypereosinophilic
Platelets Decreased - cell/mm^3	125,000 – 140,000	100,000 – 124,000	25,000 – 99,000	< 25,000
PT – increase by factor (prothrombin time)	1.0 – 1.10 x ULN**	□1.11 – 1.20 x ULN	1.21 – 1.25 x ULN	> 1.25 ULN
PTT – increase by factor (partial thromboplastin time)	1.0 – 1.2 x ULN	1.21 – 1.4 x ULN	1.41 – 1.5 x ULN	> 1.5 x ULN
Fibrinogen increase - mg/dL	400 – 500	501 – 600	> 600	--
Fibrinogen decrease - mg/dL	150 – 200	125 – 149	100 – 124	< 100 or associated with gross bleeding or disseminated intravascular coagulation (DIC)

* The laboratory values provided in the tables serve as guidelines and are dependent upon institutional normal parameters. Institutional normal reference ranges should be provided to demonstrate that they are appropriate.

** "ULN" is the upper limit of the normal range.

Urine *	Mild (Grade 1)	Moderate (Grade 2)	Severe (Grade 3)	Potentially Life Threatening (Grade 4)
Protein	Trace	1+	2+	Hospitalization or dialysis
Glucose	Trace	1+	2+	Hospitalization for hyperglycemia
Blood (microscopic) – red blood cells per high power field (rbc/hpf)	1 - 10	11 – 50	> 50 and/or gross blood	Hospitalization or packed red blood cells (PRBC) transfusion

* The laboratory values provided in the tables serve as guidelines and are dependent upon institutional normal parameters. Institutional normal reference ranges should be provided to demonstrate that they are appropriate.

C. References

1. National Cancer Institute Common Toxicity Criteria, April 30, 1999. http://ctep.cancer.gov/reporting/CTC-3.html)
2. Division of AIDS Table for Grading Severity of Adult Adverse Experiences; August 1992. (http://rcc.tech-res-intl.com/tox_tables.htm)
3. The Brighton Collaboration. Finalized Case Definitions and Guidelines. (http://brightoncollaboration.org/internet/en/index/definition___guidelines.html)
4. HIV Vaccine Trials Network Table for Grading Severity of Adverse Experiences; September 18, 2002. (http://rcc.tech-res-intl.com/tox_tables.htm)
5. Division of AIDS Table for Grading the Severity of Adult and Pediatric Adverse Events, December 2004. (http://www3.niaid.nih.gov/research/resources/DAIDSClinRsrch/PDF/Safety/DAIDSAEGradingTable.pdf)
6. Kratz A, Ferraro M, Sluss PM, Lewandrowski KB. Laboratory Reference Values. New England Journal of Medicine. 2004;351:1548-1563.

APPENDIX B: ANTIBIOTIC SUSCEPTIBILITIES

Valio Ltd. Research and Development, June 2004.
Table 1. Antibiotic susceptibility of *Lactobacillus* GG

	Minimum inhibitory concentration (MIC) µq/ml				
**Antibiotics**	Yhtyneet Laboratoriot Ltd, Finland Σ-teet, AB Biodisc	Vanderhoof et al, 1999	Klein et al, 2000 MD Plate Gram Positive, Radiometer	NCCLS Agar Dilution using Brucella agar + 5% SRBC Prof. Goldin Feb 4, 2004	Brain Heart Infusion Broth Dilution Prof. Goldin Jan 4, 1996
(Benzyl)penicillin	0.19	1.0	0.25		0.25
Ciprofloxacin	2.0	0.2	>4		1
Ofloxacin					2
Gentamicin	24.0		>32		
Ampicillin	0.50	0.5	1.0		1
Imipenem	2.0		2.0	2	1
Doxycycline	0.125				
Vancomycin	>258		>64		>32
Cefotaxime	4.0	4.0			
Erytromycine	0.094	0.25	0.5		
Amoxicillin/Clavulanate	0.5	0.5			
Cephalotin		16.0	4.0		
Tetracycline		2.0	<2.0		
Trimethoprim/Sulfamethoxazole		76.0	>4.0/ >76		
Oxacillin			1.0		
Clindamycin			0.5	1	0.25
Cloramphenicol			<4	>1 ≤8	4
Rifampin			<0.6		
Linezolid				4	
Meropenem				8	
Ertapenem				18	
Metronidazole				>16	
Moxifloxacin				1	
Trovafloxacin				0.5	
Minocycline				1	
Amp/Sulbactam 2:1				2	1
Pipercillin/ Tazobactam 4 ug/ml				1	
Ticarcillin/ Clavulanate 2 ug/ml				8	4
Pipercillin				1	0.5
Ticarcillin				8	4
Cefoxitin				>128	
Cefotetan				>256	
Cefmetazole				>128	
Cefmetazole					128
Ceftizoxime					32
Cefoxitin					>128
Cefoperazone					16

APPENDIX C: SYMPTOM DIARY

SYMPTOM DIARY

Instruction: Please check the box if YES for each symptom you have had each day. Please rate the symptom when it was bothering you the most as:

Mild - symptoms do not interfere with your daily activities, no medical therapy required.

Moderate - symptoms which may interfere with you daily activities, no or minimal medical therapy required

Severe - symptoms which interrupt your daily activities, medical therapy required, hospitalization possible

Very severe - symptoms which cause extreme limitations in your daily activity that required medical therapy and hospitalization

Please write the rating on the line next to check box.

Symptoms and Medications	Monday<br>__ __/__ __	Tuesday<br>__ __/__ __	Wednesday<br>__ __/__ __	Thursday<br>__ __/__ __	Friday<br>__ __/__ __	Saturday<br>__ __/__ __	Sunday<br>__ __/__ __
Bloating	☐____	☐____	☐____	☐____	☐____	☐____	☐____
Gas	☐____	☐____	☐____	☐____	☐____	☐____	☐____
Intestinal rumbling	☐____	☐____	☐____	☐____	☐____	☐____	☐____
Diarrhea	☐____	☐____	☐____	☐____	☐____	☐____	☐____
Blood in Stool	☐____	☐____	☐____	☐____	☐____	☐____	☐____
Abd. Cramps or Pain	☐____	☐____	☐____	☐____	☐____	☐____	☐____
Nausea	☐____	☐____	☐____	☐____	☐____	☐____	☐____
Vomiting	☐____	☐____	☐____	☐____	☐____	☐____	☐____
Loss of Appetite	☐____	☐____	☐____	☐____	☐____	☐____	☐____
Abnormal Taste	☐____	☐____	☐____	☐____	☐____	☐____	☐____
Heartburn	☐____	☐____	☐____	☐____	☐____	☐____	☐____
Constipation	☐____	☐____	☐____	☐____	☐____	☐____	☐____
Skin Rash	☐____	☐____	☐____	☐____	☐____	☐____	☐____
Other:__________	☐____	☐____	☐____	☐____	☐____	☐____	☐____
Other:__________	☐____	☐____	☐____	☐____	☐____	☐____	☐____
Other:__________	☐____	☐____	☐____	☐____	☐____	☐____	☐____
Did you take any medications (including any over-the-counter or prescription drugs, other than LGG)	☐ Yes ☐ No<br>If Yes, describe:<br>1.____________<br>2.____________<br>3.____________<br>4.____________	☐ Yes ☐ No<br>If Yes, describe:<br>1.____________<br>2.____________<br>3.____________<br>4.____________	☐ Yes ☐ No<br>If Yes, describe:<br>1.____________<br>2.____________<br>3.____________<br>4.____________	☐ Yes ☐ No<br>If Yes, describe:<br>1.____________<br>2.____________<br>3.____________<br>4.____________	☐ Yes ☐ No<br>If Yes, describe:<br>1.____________<br>2.____________<br>3.____________<br>4.____________	☐ Yes ☐ No<br>If Yes, describe:<br>1.____________<br>2.____________<br>3.____________<br>4.____________	☐ Yes ☐ No<br>If Yes, describe<br>1.____________<br>2.____________<br>3.____________<br>4.____________

APPENDIX D:

Amendment 1 Summary of Changes: 5/17/2010-10/15/2010

Amendment 1

Title: Open Label Study to Evaluate the Safety of Lactobacillus Rhamnosus GG ATCC 53103 (LGG) in Elderly Subjects

Version Number: 1.1

Date: October 15, 2010

The following changes are implemented in Protocol B1: Open Label Study to Evaluate the Safety of Lactobacillus Rhamnosus GG ATCC 53103 (LGG) in Elderly Subjects. These changes were requested in the FDA letter re: IND 14377 received August 18, 2010.

APPENDIX D AMENDMENT 1 SUMMARY OF CHANGES: 5/17/10 – 10/15/10

**Applicable Sections**	**Version 1.0, Serial Number 001, May 17, 2010**		**Version 1.1, Serial Number 002, October 15, 2010**	
	**Page #**	**States**	**Page #**	**States**
Cover page	1	Version Number: 1.0<br>Serial Number: 001<br>Date: May 17, 2010	1	Version Number: 1.1<br>Serial Number: 002<br>Date: October 15, 2010
Table of Contents	6		6	Appendix D Amendment 1 Summary of Changes 5/17/10 – 10/15/10 added
Protocol Summary	8	Enrolled subjects will take 2 LGG capsules orally, twice a day, for 28 days, as outpatients.	8	Enrolled subjects will take 1 LGG capsule orally, twice a day, for 28 days, as outpatients.
Description of Study Design	10	Baseline Visit box states “Randomize”	10	Word “Randomize” was removed from text in the Baseline Visit box.
1. Key Roles	12	Principal Investigator<br>50 Staniford Street, Suite 1054	12	Principal Investigator<br>Patricia L. Hibberd, MD, PhD<br>50 Staniford Street, Suite 401
1. Key Roles	12	Christine Botelho, MPH<br>50 Staniford Street, Suite 1056<br>Email: cbotelo@partners.org	12-13	Christine Botelho, MPH<br>50 Staniford Street, Suite 401<br>Email: cbotelho@partners.org
1. Key Roles	13	Statistician and Data Manager:<br>Anne-Maria Fiorino, MS<br>50 Staniford Street, Suite 1056	13	Statistician and Data Manager:<br>Anne-Maria Fiorino, MS<br>50 Staniford Street, Suite 401
1. Key Roles	13	Study Coordinator:<br>Irina Andreyeva<br>50 Staniford Street, Suite 1056	13	Study Coordinator:<br>Irina Andreyeva<br>50 Staniford Street, Suite 401
5. Study Screening and Enrollment	41		41	Duplicative word “subject” was removed in first paragraph.
5.2 Subject Inclusion Criteria	41		41-42	The following text has been added:<br>• Is community dwelling for the past two years<br>• Has received routine physical in the past two years<br>• Has no new chronic conditions

				in the past two years<br>• Identifies a primary care clinician.<br>• Has received recommended preventive services (Task Force for Clinical Preventative Services) for vaccination and cancer prevention/detection, e.g.:<br>o Pneumococcal vaccination<br>o Mammography<br>o Screening colonoscopy for colon cancer
5.3 Subject Exclusion Criteria	42		42-43	The following text has been added:<br>• Current or within the last 2 years, any episode of bowel leak, acute abdomen, diverticulitis, colitis, bloody bowel movements or peptic ulcer disease, including any surgical procedure or current prescription medications for any of these conditions.<br>• Current or within the last four weeks, active bowel disease such as an episode of infectious or non-infectious diarrhea, constipation or vomiting lasting more than 12 hours or current prescription medications for any of these conditions.<br>• Any history of gastric or intestinal dysmotility, slowed transit time, variable small intestinal permeability, pancreatitis, history of gastrointestinal tract cancer or metastasis or inflammatory bowel disease or current prescription medications for any of these conditions
5.3 Subject	42		43	The following text was deleted:

Exclusion Criteria				• Active bowel leak, acute abdomen, colitis or active GI disease or history of gastric or intestinal dysmotility, slowed transit time, variable small intestinal permeability, pancreatitis, history of gastrointestinal tract cancer or inflammatory bowel disease
5.3 Subject Exclusion Criteria	42	• History of Hepatitis B or Hepatitis C infections, cirrhosis, or chronic liver disease.	43	• Any history of Hepatitis B or Hepatitis C infections, cirrhosis, or chronic liver disease
5.3 Subject Exclusion Criteria	43	• Active (TB)	43	• Active tuberculosis (TB), defined as undergoing a work up for suspected active TB infection or currently on treatment for active TB
5.4 Enrollment	43		44	The following text was added:<br>In addition, subjects who enroll in this study will be informed of an optional sub study
14.2 Institutional Review Board	63	Any amendments to the protocol which need formal approval as required by local law will be approved by this committee.	64	Any amendments to the protocol which need formal approval as required by federal law will be approved by this committee.

Amendment 2 Summary of Changes: 10/16/2010- 6/6/2011

Amendment 2

Title: Open Label Study to Evaluate the Safety of Lactobacillus Rhamnosus GG ATCC 53103 (LGG) in Elderly Subjects

Version Number: 1.1

Date: June 6, 2011

The following changes are implemented in Protocol B1: Open Label Study to Evaluate the Safety of Lactobacillus Rhamnosus GG ATCC 53103 (LGG) in Elderly Subjects. These changes re frequency of DSMB meetings were reviewed and approved by the DSMB on 3/31/2011.

APPENDIX D AMENDMENT 2 SUMMARY OF CHANGES: 10/16/10 – 6/6/11

**Applicable Sections**	**Version 1.0, Serial Number 002, October 15, 2010, 2010**		**Version 1.1, Serial Number 003, June 6, 2011**	
	**Page #**	**States**	**Page #**	**States**
Cover page	1	Version Number: 1.1<br>Serial Number: 002<br>Date: October 15, 2010	1	Version Number: 1.1<br>Serial Number: 003<br>Date: June 6, 2011
Table of Contents	6		6	Appendix D Amendment 2 Summary of Changes 10/15/10 – 6/6/11 added
4. Study Design	40	The DSMB will review the safety data approximately every three months.	40	The DSMB will review the safety data approximately every six months.
9.7 Safety oversight and study termination	58	The DSMB will meet approximately every 3 months	58	The DSMB will meet approximately every 6 months.
11.2 Interim analysis	61	Interim safety data will be provided to the DSMB approximately every 3 months.	61	Interim safety data will be provided to the DSMB approximately every 6 months.
15.2 Types of data	66	Safety reports will be presented to the DSMB approximately every 3 months.	66	Safety reports will be presented to the DSMB approximately every 6 months.
