# Supplementary material for: No Evidence of Harms of Probiotic Lactobacillus rhamnosus GG ATCC 53103 in Healthy Elderly—A Phase I Open Label Study to Assess Safety, Tolerability and Cytokine Responses
Source: PLoS One. 2014 Dec 1;9(12):e113456. doi: 10.1371/journal.pone.0113456 (PMC4249962; doi:10.1371/journal.pone.0113456)
Supplement: Materials and Methods S1 — Detailed materials and methods for eliciting possible harms including forms used to collec9t this information during study visits and telephone calls and the daily symptom diary. (PDF) [file pone.0113456.s003.pdf]

Study ID: __ __ __ -__ __

Visit Date: __ __/__ __/__ __ __ __

BASELINE VISIT (Day 0)

INTERIM HISTORY

1. Any significant changes in physical health since last visit? ☐ **Yes** ☐ **No**

   If **Yes**, please describe: ____________________

2. Any MD/ER visits and/or hospitalization since last visit? ☐ **Yes** ☐ **No**

   If **Yes**, please describe: ____________________

3. Any new prescription medications since last visit? ☐ **Yes** ☐ **No**

   If **Yes**, list below:

Medication	Route	Reason for use	Start Date MM/DD/YY	Continuing	If No, Stop Date MM/DD/YY
				☐ Yes ☐ No	
				☐ Yes ☐ No	
				☐ Yes ☐ No	
				☐ Yes ☐ No	

4. Has the volunteer consumed any probiotics, yogurt or other products containing probiotics such as smoothies or cereal since the last visit? ☐ **Yes** ☐ **No**

   If **Yes**, when how much: ____________________

5. When did you last eat or drink? Time: __ __:__ __ Date: __ __ / __ __

   Please describe: ____________________

PHYSICAL EXAM

1. Oral Temperature: ___ ___ ___. ___ ° F
2. Heart rate: ___ ___ ___beats per minute
3. Respiratory rate: ___ ___ breaths per minute
4. Blood pressure: ___ ___ ___ / ___ ___ ___
5. Weight: ___ ___ ___ . ___kg
6. Height: ___ ___ ___.___ cm

**Body System**	**N**	**ABN**	**Not Done**	**If "Abnormal", please describe:**
7. HEENT	☐	☐	☐	______________________
8. Neck	☐	☐	☐	______________________
9. Heart	☐	☐	☐	______________________
10. Lungs	☐	☐	☐	______________________
11. Abdomen	☐	☐	☐	______________________
12. Skin	☐	☐	☐	______________________
13. Musculoskeletal	☐	☐	☐	______________________
14. Neurologic	☐	☐	☐	______________________
15. Lymph Nodes	☐	☐	☐	______________________
16. Vascular	☐	☐	☐	______________________
17. Other, Specify: ______________ ______________	☐	☐	☐	______________________ ______________________

Comments:
____
____

Form Completed by ______________________ Date __ __ / __ __ / __ __ __ __

Study ID: __ __ __ -__ __

Visit Date: __ __/__ __/__ __ __ __

REVIEW OF SYMPTOMS

Have the following symptoms occurred, since your last visit?			Was treatment required?		
	No	Yes	No	Yes	If **Yes**, comment:
a. Bloating	☐	☐	☐	☐	______________
b. Gas	☐	☐	☐	☐	______________
c. Intestinal Rumbling	☐	☐	☐	☐	______________
d. Diarrhea	☐	☐	☐	☐	______________
e. Blood in Stool	☐	☐	☐	☐	______________
f. Abdominal Cramps/Pain	☐	☐	☐	☐	______________
g. Nausea	☐	☐	☐	☐	______________
h. Vomiting	☐	☐	☐	☐	______________
i. Loss of Appetite	☐	☐	☐	☐	______________
j. Abnormal Taste	☐	☐	☐	☐	______________
k. Heartburn	☐	☐	☐	☐	______________
l. Constipation	☐	☐	☐	☐	______________
m. Skin Rash	☐	☐	☐	☐	______________
n. Fever	☐	☐	☐	☐	______________
o. Runny nose/nasal congestion	☐	☐	☐	☐	______________
p. Sore Throat	☐	☐	☐	☐	______________
q. Cough	☐	☐	☐	☐	______________
r. Headache	☐	☐	☐	☐	______________

	**No**	**Yes**	**No**	**Yes**	If **Yes**, comment:
s. Chills	☐	☐	☐	☐	____________________
t. Muscle Aches	☐	☐	☐	☐	____________________
u. Other: ________________	☐	☐	☐	☐	____________________

Comments:

____
____
____
____
____
____
____
____

Form Completed by ________________________ Date __ __ / __ __ / __ __ __ __ (mm/dd/yyyyy)

Study ID: __ __ __-__ __

SYMPTOM DIARY

Instruction: Please check the box if YES for each symptom you have had each day. Please rate the symptom when it was bothering you the most as:

Mild - symptoms do not interfere with your daily activities, no medical therapy required.

Moderate - symptoms which may interfere with your daily activities, no or minimal medical therapy required

Severe - symptoms which interrupt your daily activities, medical therapy required, hospitalization possible

Very severe - symptoms which cause extreme limitations in your daily activity that required medical therapy and hospitalization

Please write the rating on the line next to check box.

Symptoms and Medications	Monday __ __/__ __	Tuesday __ __/__ __	Wednesday __ __/__ __	Thursday __ __/__ __	Friday __ __/__ __	Saturday __ __/__ __	Sunday __ __/__ __
Bloating	☐________	☐________	☐________	☐________	☐________	☐________	☐________
Gas	☐________	☐________	☐________	☐________	☐________	☐________	☐________
Intestinal rumbling	☐________	☐________	☐________	☐________	☐________	☐________	☐________
Diarrhea	☐________	☐________	☐________	☐________	☐________	☐________	☐________
Blood in Stool	☐________	☐________	☐________	☐________	☐________	☐________	☐________
Abd. Cramps or Pain	☐________	☐________	☐________	☐________	☐________	☐________	☐________
Nausea	☐________	☐________	☐________	☐________	☐________	☐________	☐________
Vomiting	☐________	☐________	☐________	☐________	☐________	☐________	☐________
Loss of Appetite	☐________	☐________	☐________	☐________	☐________	☐________	☐________
Abnormal Taste	☐________	☐________	☐________	☐________	☐________	☐________	☐________
Heartburn	☐________	☐________	☐________	☐________	☐________	☐________	☐________
Constipation	☐________	☐________	☐________	☐________	☐________	☐________	☐________
Skin Rash	☐________	☐________	☐________	☐________	☐________	☐________	☐________
Other:__________	☐________	☐________	☐________	☐________	☐________	☐________	☐________
Other:__________	☐________	☐________	☐________	☐________	☐________	☐________	☐________
Other:__________	☐________	☐________	☐________	☐________	☐________	☐________	☐________
Did you take any new medications (including any over-the-counter or prescription drugs, other than LGG)	☐ Yes ☐ No<br>If Yes, describe:<br>1.__________<br>2.__________<br>3.__________<br>4.__________	☐ Yes ☐ No<br>If Yes, describe:<br>1.__________<br>2.__________<br>3.__________<br>4.__________	☐ Yes ☐ No<br>If Yes, describe:<br>1.__________<br>2.__________<br>3.__________<br>4.__________	☐ Yes ☐ No<br>If Yes, describe:<br>1.__________<br>2.__________<br>3.__________<br>4.__________	☐ Yes ☐ No<br>If Yes, describe:<br>1.__________<br>2.__________<br>3.__________<br>4.__________	☐ Yes ☐ No<br>If Yes, describe:<br>1.__________<br>2.__________<br>3.__________<br>4.__________	☐ Yes ☐ No<br>If Yes, describe:<br>1.__________<br>2.__________<br>3.__________<br>4.__________

Follow-Up Phone Script

Hello, this is _______ from Massachusetts General Hospital. I want to thank you for your participation in our research study and am calling to find out how you are doing.

This is your [put in visit day/month here] and I would like to ask you a few questions regarding your health. **[Use Follow-up Phone Call form: (Interim History), ask subject about their current health status]**

1. Have you had any significant changes in your physical health since your last visit or phone call?
If **NO:** go on to next question
If **YES:** document changes and let subject know that the study MD may contact subject to review these changes. Obtain time that would be convenient for subject to speak with study MD.

2. Have you had any MD/ER visits or any hospitalizations since your last visit or phone call?
If **NO:** go on to next question
If **YES:** document type of visit and let subject know that the study MD may contact subject to go over visit details. Obtain time that would be convenient for subject to speak with study MD.

3. Have you started any new prescription medication since your last visit or phone call?
If **NO:** go on to next question
If **YES:** document new prescription medication and let subject know that the study MD may contact subject to go over details of medication use. Obtain time that would be convenient for subject to speak with study MD.

4. Have you consumed any yogurt or products containing probiotics such as smoothies or cereal since your last visit or phone call?
If **NO:** proceed to adverse event assessment
If **YES:** probe for further details from subject

Now I want to ask you a few questions about any symptoms you may or may not be having. Please answer yes or no to the following questions:**[Use Adverse Event Assessment Form to evaluate symptoms]**

Have you had…?

a. Bloating - If **NO:** go on to next question
If **YES:** ask when symptom started and if/when symptom stopped.
If **symptom stopped**, document date. Let subject know that Study MD may be contacting them for further information. Ask subject if the symptom at its worst was **Mild** (symptom did not interfere with daily activities), no medical therapy required), **Moderate** (symptom did interfere with daily activities, no or minimal medical therapy required),

Severe (symptom interrupted daily activities, medical therapy required, hospitalization possible) or **Very Severe** (symptom caused extreme limitations in daily activity that required medical therapy and hospitalization).

If **symptom still ongoing**, document symptom start date. Let subject know that Study MD may be contacting them for further information. Ask subject if the symptom at its worst is **Mild** (symptom does not interfere with daily activities), no medical therapy required), **Moderate** (symptom does interfere with daily activities, no or minimal medical therapy required), **Severe** (symptom is interrupting daily activities, medical therapy is required, hospitalization possible) or **Very Severe** (symptom is causing extreme limitations in daily activity that required medical therapy and hospitalization). Should subject report the symptom as **Severe or Very Severe** page the Study MD immediately. Let subject know the Study MD has been paged and will contact them shortly. Let subject know that if they feel they cannot wait until the Study MD contacts them they should contact their PCP or go directly to the nearest Emergency Room.

b. Gas - If **NO:** go on to next question

If **YES:** ask when symptom started and if/when symptom stopped.

If **symptom stopped**, document date. Let subject know that Study MD may be contacting them for further information. Ask subject if the symptom at its worst was **Mild** (symptom did not interfere with daily activities), no medical therapy required), **Moderate** (symptom did interfere with daily activities, no or minimal medical therapy required), **Severe** (symptom interrupted daily activities, medical therapy required, hospitalization possible) or **Very Severe** (symptom caused extreme limitations in daily activity that required medical therapy and hospitalization).

If **symptom still ongoing**, document symptom start date. Let subject know that Study MD may be contacting them for further information. Ask subject if the symptom at its worst is **Mild** (symptom does not interfere with daily activities), no medical therapy required), **Moderate** (symptom does interfere with daily activities, no or minimal medical therapy required), **Severe** (symptom is interrupting daily activities, medical therapy is required, hospitalization possible) or **Very Severe** (symptom is causing extreme limitations in daily activity that required medical therapy and hospitalization). Should subject report the symptom as **Severe or Very Severe** page the Study MD immediately. Let subject know the Study MD has been paged and will contact them shortly. Let subject know that if they feel they cannot wait until the Study MD contacts them they should contact their PCP or go directly to the nearest Emergency Room.

c. Intestinal rumbling -

If **NO:** go on to next question

If **YES:** ask when symptom started and if/when symptom stopped.

If **symptom stopped**, document date. Let subject know that Study MD may be contacting them for further information. Ask subject if the symptom at its worst was **Mild** (symptom did not interfere with daily

activities), no medical therapy required), **Moderate** (symptom did interfere with daily activities, no or minimal medical therapy required), **Severe** (symptom interrupted daily activities, medical therapy required, hospitalization possible) or **Very Severe** (symptom caused extreme limitations in daily activity that required medical therapy and hospitalization).

If **symptom still ongoing**, document symptom start date. Let subject know that Study MD may be contacting them for further information. Ask subject if the symptom at its worst is **Mild** (symptom does not interfere with daily activities), no medical therapy required), **Moderate** (symptom does interfere with daily activities, no or minimal medical therapy required), **Severe** (symptom is interrupting daily activities, medical therapy is required, hospitalization possible) or **Very Severe** (symptom is causing extreme limitations in daily activity that required medical therapy and hospitalization). Should subject report the symptom as **Severe or Very Severe** page the Study MD immediately. Let subject know the Study MD has been paged and will contact them shortly. Let subject know that if they feel they cannot wait until the Study MD contacts them they should contact their PCP or go directly to the nearest Emergency Room.

d. Diarrhea - If **NO:** go on to next question

If **YES:** ask when symptom started and if/when symptom stopped.

If **symptom stopped**, document date. Let subject know that Study MD may be contacting them for further information. Ask subject if the symptom at its worst was **Mild** (symptom did not interfere with daily activities), no medical therapy required), **Moderate** (symptom did interfere with daily activities, no or minimal medical therapy required), **Severe** (symptom interrupted daily activities, medical therapy required, hospitalization possible) or **Very Severe** (symptom caused extreme limitations in daily activity that required medical therapy and hospitalization).

If **symptom still ongoing**, document symptom start date. Let subject know that Study MD may be contacting them for further information. Ask subject if the symptom at its worst is **Mild** (symptom does not interfere with daily activities), no medical therapy required), **Moderate** (symptom does interfere with daily activities, no or minimal medical therapy required), **Severe** (symptom is interrupting daily activities, medical therapy is required, hospitalization possible) or **Very Severe** (symptom is causing extreme limitations in daily activity that required medical therapy and hospitalization). Should subject report the symptom as **Severe or Very Severe** page the Study MD immediately. Let subject know the Study MD has been paged and will contact them shortly. Let subject know that if they feel they cannot wait until the Study MD contacts them they should contact their PCP or go directly to the nearest Emergency Room.

e. Blood in stool -

If **NO:** go on to next question

If **YES:** ask when symptom started and if/when symptom stopped.

If **symptom stopped**, document date. Let subject know that Study MD may be contacting them for further information. Ask subject if the symptom at its worst was **Mild** (symptom did not interfere with daily activities), no medical therapy required), **Moderate** (symptom did interfere with daily activities, no or minimal medical therapy required), **Severe** (symptom interrupted daily activities, medical therapy required, hospitalization possible) or **Very Severe** (symptom caused extreme limitations in daily activity that required medical therapy and hospitalization).

If **symptom still ongoing**, document symptom start date. Let subject know that Study MD may be contacting them for further information. Ask subject if the symptom at its worst is **Mild** (symptom does not interfere with daily activities), no medical therapy required), **Moderate** (symptom does interfere with daily activities, no or minimal medical therapy required), **Severe** (symptom is interrupting daily activities, medical therapy is required, hospitalization possible) or **Very Severe** (symptom is causing extreme limitations in daily activity that required medical therapy and hospitalization). Should subject report the symptom as **Severe or Very Severe** page the Study MD immediately. Let subject know the Study MD has been paged and will contact them shortly. Let subject know that if they feel they cannot wait until the Study MD contacts them they should contact their PCP or go directly to the nearest Emergency Room.

f. Abdominal Cramps or Pain -

If **NO:** go on to next question

If **YES:** ask when symptom started and if/when symptom stopped.

If **symptom stopped**, document date. Let subject know that Study MD may be contacting them for further information. Ask subject if the symptom at its worst was **Mild** (symptom did not interfere with daily activities), no medical therapy required), **Moderate** (symptom did interfere with daily activities, no or minimal medical therapy required), **Severe** (symptom interrupted daily activities, medical therapy required, hospitalization possible) or **Very Severe** (symptom caused extreme limitations in daily activity that required medical therapy and hospitalization).

If **symptom still ongoing**, document symptom start date. Let subject know that Study MD may be contacting them for further information. Ask subject if the symptom at its worst is **Mild** (symptom does not interfere with daily activities), no medical therapy required), **Moderate** (symptom does interfere with daily activities, no or minimal medical therapy required), **Severe** (symptom is interrupting daily activities, medical therapy is required, hospitalization possible) or **Very Severe** (symptom is causing extreme limitations in daily activity that required medical therapy and hospitalization). Should subject report the symptom as **Severe or Very Severe** page the Study MD immediately. Let subject know the Study MD has been paged and will contact them shortly. Let subject know that if they feel they cannot wait until the Study MD

contacts them they should contact their PCP or go directly to the nearest Emergency Room.

g. Nausea - If **NO:** go on to next question

If **YES:** ask when symptom started and if/when symptom stopped.

If **symptom stopped**, document date. Let subject know that Study MD may be contacting them for further information. Ask subject if the symptom at its worst was **Mild** (symptom did not interfere with daily activities), no medical therapy required), **Moderate** (symptom did interfere with daily activities, no or minimal medical therapy required), **Severe** (symptom interrupted daily activities, medical therapy required, hospitalization possible) or **Very Severe** (symptom caused extreme limitations in daily activity that required medical therapy and hospitalization).

If **symptom still ongoing**, document symptom start date. Let subject know that Study MD may be contacting them for further information. Ask subject if the symptom at its worst is **Mild** (symptom does not interfere with daily activities), no medical therapy required), **Moderate** (symptom does interfere with daily activities, no or minimal medical therapy required), **Severe** (symptom is interrupting daily activities, medical therapy is required, hospitalization possible) or **Very Severe** (symptom is causing extreme limitations in daily activity that required medical therapy and hospitalization). Should subject report the symptom as **Severe or Very Severe** page the Study MD immediately. Let subject know the Study MD has been paged and will contact them shortly. Let subject know that if they feel they cannot wait until the Study MD contacts them they should contact their PCP or go directly to the nearest Emergency Room.

h. Vomiting - If **NO:** go on to next question

If **YES:** ask when symptom started and if/when symptom stopped.

If **symptom stopped**, document date. Let subject know that Study MD may be contacting them for further information. Ask subject if the symptom at its worst was **Mild** (symptom did not interfere with daily activities), no medical therapy required), **Moderate** (symptom did interfere with daily activities, no or minimal medical therapy required), **Severe** (symptom interrupted daily activities, medical therapy required, hospitalization possible) or **Very Severe** (symptom caused extreme limitations in daily activity that required medical therapy and hospitalization).

If **symptom still ongoing**, document symptom start date. Let subject know that Study MD may be contacting them for further information. Ask subject if the symptom at its worst is **Mild** (symptom does not interfere with daily activities), no medical therapy required), **Moderate** (symptom does interfere with daily activities, no or minimal medical therapy required), **Severe** (symptom is interrupting daily activities, medical therapy is required, hospitalization possible) or **Very Severe** (symptom is causing extreme limitations in daily activity that required medical therapy and hospitalization). Should subject report the symptom as **Severe or Very Severe** page the Study MD immediately. Let subject know the Study MD has been paged and will contact them shortly. Let

subject know that if they feel they cannot wait until the Study MD contacts them they should contact their PCP or go directly to the nearest Emergency Room.

i. Loss of Appetite -

If **NO:** go on to next question

If **YES:** ask when symptom started and if/when symptom stopped.

If **symptom stopped**, document date. Let subject know that Study MD may be contacting them for further information. Ask subject if the symptom at its worst was **Mild** (symptom did not interfere with daily activities), no medical therapy required), **Moderate** (symptom did interfere with daily activities, no or minimal medical therapy required), **Severe** (symptom interrupted daily activities, medical therapy required, hospitalization possible) or **Very Severe** (symptom caused extreme limitations in daily activity that required medical therapy and hospitalization).

If **symptom still ongoing**, document symptom start date. Let subject know that Study MD may be contacting them for further information. Ask subject if the symptom at its worst is **Mild** (symptom does not interfere with daily activities), no medical therapy required), **Moderate** (symptom does interfere with daily activities, no or minimal medical therapy required), **Severe** (symptom is interrupting daily activities, medical therapy is required, hospitalization possible) or **Very Severe** (symptom is causing extreme limitations in daily activity that required medical therapy and hospitalization). Should subject report the symptom as **Severe or Very Severe** page the Study MD immediately. Let subject know the Study MD has been paged and will contact them shortly. Let subject know that if they feel they cannot wait until the Study MD contacts them they should contact their PCP or go directly to the nearest Emergency Room.

j. Abnormal Taste -

If **NO:** go on to next question

If **YES:** ask when symptom started and if/when symptom stopped.

If **symptom stopped**, document date. Let subject know that Study MD may be contacting them for further information. Ask subject if the symptom at its worst was **Mild** (symptom did not interfere with daily activities), no medical therapy required), **Moderate** (symptom did interfere with daily activities, no or minimal medical therapy required), **Severe** (symptom interrupted daily activities, medical therapy required, hospitalization possible) or **Very Severe** (symptom caused extreme limitations in daily activity that required medical therapy and hospitalization).

If **symptom still ongoing**, document symptom start date. Let subject know that Study MD may be contacting them for further information. Ask subject if the symptom at its worst is **Mild** (symptom does not interfere with daily activities), no medical therapy required), **Moderate** (symptom does interfere with daily activities, no or minimal medical therapy required), **Severe** (symptom is interrupting daily activities, medical therapy is required, hospitalization possible) or **Very Severe** (symptom is causing extreme limitations in daily activity that required medical

therapy and hospitalization). Should subject report the symptom as **Severe or Very Severe** page the Study MD immediately. Let subject know the Study MD has been paged and will contact them shortly. Let subject know that if they feel they cannot wait until the Study MD contacts them they should contact their PCP or go directly to the nearest Emergency Room.

k. Heartburn -

If **NO:** go on to next question

If **YES:** ask when symptom started and if/when symptom stopped.

If **symptom stopped**, document date. Let subject know that Study MD may be contacting them for further information. Ask subject if the symptom at its worst was **Mild** (symptom did not interfere with daily activities), no medical therapy required), **Moderate** (symptom did interfere with daily activities, no or minimal medical therapy required), **Severe** (symptom interrupted daily activities, medical therapy required, hospitalization possible) or **Very Severe** (symptom caused extreme limitations in daily activity that required medical therapy and hospitalization).

If **symptom still ongoing**, document symptom start date. Let subject know that Study MD may be contacting them for further information. Ask subject if the symptom at its worst is **Mild** (symptom does not interfere with daily activities), no medical therapy required), **Moderate** (symptom does interfere with daily activities, no or minimal medical therapy required), **Severe** (symptom is interrupting daily activities, medical therapy is required, hospitalization possible) or **Very Severe** (symptom is causing extreme limitations in daily activity that required medical therapy and hospitalization). Should subject report the symptom as **Severe or Very Severe** page the Study MD immediately. Let subject know the Study MD has been paged and will contact them shortly. Let subject know that if they feel they cannot wait until the Study MD contacts them they should contact their PCP or go directly to the nearest Emergency Room.

l. Constipation -

If **NO:** go on to next question

If **YES:** ask when symptom started and if/when symptom stopped.

If **symptom stopped**, document date. Let subject know that Study MD may be contacting them for further information. Ask subject if the symptom at its worst was **Mild** (symptom did not interfere with daily activities), no medical therapy required), **Moderate** (symptom did interfere with daily activities, no or minimal medical therapy required), **Severe** (symptom interrupted daily activities, medical therapy required, hospitalization possible) or **Very Severe** (symptom caused extreme limitations in daily activity that required medical therapy and hospitalization).

If **symptom still ongoing**, document symptom start date. Let subject know that Study MD may be contacting them for further information. Ask subject if the symptom at its worst is **Mild** (symptom does not interfere with daily activities), no medical therapy required), **Moderate** (symptom does interfere with daily activities, no or minimal medical therapy

required), **Severe** (symptom is interrupting daily activities, medical therapy is required, hospitalization possible) or **Very Severe** (symptom is causing extreme limitations in daily activity that required medical therapy and hospitalization). Should subject report the symptom as **Severe or Very Severe** page the Study MD immediately. Let subject know the Study MD has been paged and will contact them shortly. Let subject know that if they feel they cannot wait until the Study MD contacts them they should contact their PCP or go directly to the nearest Emergency Room.

m. Skin Rash -

If **NO:** go on to next question

If **YES:** ask when symptom started and if/when symptom stopped.

If **symptom stopped**, document date. Let subject know that Study MD may be contacting them for further information. Ask subject if the symptom at its worst was **Mild** (symptom did not interfere with daily activities), no medical therapy required), **Moderate** (symptom did interfere with daily activities, no or minimal medical therapy required), **Severe** (symptom interrupted daily activities, medical therapy required, hospitalization possible) or **Very Severe** (symptom caused extreme limitations in daily activity that required medical therapy and hospitalization).

If **symptom still ongoing**, document symptom start date. Let subject know that Study MD may be contacting them for further information. Ask subject if the symptom at its worst is **Mild** (symptom does not interfere with daily activities), no medical therapy required), **Moderate** (symptom does interfere with daily activities, no or minimal medical therapy required), **Severe** (symptom is interrupting daily activities, medical therapy is required, hospitalization possible) or **Very Severe** (symptom is causing extreme limitations in daily activity that required medical therapy and hospitalization). Should subject report the symptom as **Severe or Very Severe** page the Study MD immediately. Let subject know the Study MD has been paged and will contact them shortly. Let subject know that if they feel they cannot wait until the Study MD contacts them they should contact their PCP or go directly to the nearest Emergency Room.

n. Other - If **NO:** go on to next question

If **YES:** ask when symptom started and if/when symptom stopped.

If **symptom stopped**, document date. Let subject know that Study MD may be contacting them for further information. Ask subject if the symptom at its worst was **Mild** (symptom did not interfere with daily activities), no medical therapy required), **Moderate** (symptom did interfere with daily activities, no or minimal medical therapy required), **Severe** (symptom interrupted daily activities, medical therapy required, hospitalization possible) or **Very Severe** (symptom caused extreme limitations in daily activity that required medical therapy and hospitalization).

If **symptom still ongoing**, document symptom start date. Let subject know that Study MD may be contacting them for further information. Ask subject if the symptom at its worst is **Mild** (symptom does not interfere

with daily activities), no medical therapy required), **Moderate** (symptom does interfere with daily activities, no or minimal medical therapy required), **Severe** (symptom is interrupting daily activities, medical therapy is required, hospitalization possible) or **Very Severe** (symptom is causing extreme limitations in daily activity that required medical therapy and hospitalization). Should subject report the symptom as **Severe or Very Severe** page the Study MD immediately. Let subject know the Study MD has been paged and will contact them shortly. Let subject know that if they feel they cannot wait until the Study MD contacts them they should contact their PCP or go directly to the nearest Emergency Room.

Do you have any questions or concerns about the study?
If **NO** thank subject and remind them of next study visit/call and remind subject of contact information for study related questions.
If **YES** probe further….once done remind them of next study visit/call and remind subject of contact information for study related questions.
